# Metabolic and Immune Activation Effects of Treatment Interruption in Chronic HIV-1 Infection: Implications for Cardiovascular Risk

**DOI:** 10.1371/journal.pone.0002021

**Published:** 2008-04-23

**Authors:** Pablo Tebas, William Keith Henry, Roy Matining, Deborah Weng-Cherng, John Schmitz, Hernan Valdez, Nasreen Jahed, Laurie Myers, William G. Powderly, David Katzenstein

**Affiliations:** 1 University of Pennsylvania, Philadelphia, Pennsylvania, United States of America; 2 Hennepin County Medical Center, University of Minnesota, Minneapolis, Minnesota, United States of America; 3 Harvard School of Public Health, Boston, Massachusetts, United States of America; 4 University of North Carolina, Chapel Hill, North Carolina, United States of America; 5 Case Western Reserve University, Cleveland, Ohio, United States of America; 6 Social and Scientific Systems Inc, Silver Spring, Maryland, United States of America; 7 Frontier Science & Technology Research Foundation, Inc (FSTRF), Amherst, New York, United States of America; 8 University College Dublin, Dublin, Ireland; 9 Stanford University, Stanford, California, United States of America; University of New South Wales, Australia

## Abstract

**Background:**

Concern about costs and antiretroviral therapy (ART)-associated toxicities led to the consideration of CD4 driven strategies for the management of HIV. That approach was evaluated in the SMART trial that reported an unexpected increase of cardiovascular events after treatment interruption (TI). Our goal was to evaluate fasting metabolic changes associated with interruption of antiretroviral therapy and relate them to changes of immune activation markers and cardiovascular risk.

**Methodology:**

ACTG 5102 enrolled 47 HIV-1-infected subjects on stable ART, with <200 HIV RNA copies/mL and CD4 cell count ≥500 cells/µL. Subjects were randomly assigned to continue ART for 18 weeks with or without 3 cycles of interleukin-2 (IL-2) (cycle = 4.5 million IU sc BID x 5 days every 8 weeks). After 18 weeks ART was discontinued in all subjects until the CD4 cell count dropped below 350 cells/µL. Glucose and lipid parameters were evaluated every 8 weeks initially and at weeks 2, 4, 8 and every 8 weeks after TI. Immune activation was evaluated by flow-cytometry and soluble TNFR2 levels.

**Principal Findings:**

By week 8 of TI, levels of total cholesterol (TC) (median (Q1, Q3) (−0.73 (−1.19, −0.18) mmol/L, p<0.0001), LDL, HDL cholesterol (−0.36(−0.73,−0.03)mmol/L, p = 0.0007 and −0.05(−0.26,0.03), p = 0.0033, respectively) and triglycerides decreased (−0.40 (−0.84, 0.07) mmol/L, p = 0.005). However the TC/HDL ratio remained unchanged (−0.09 (−1.2, 0.5), p = 0.2). Glucose and insulin levels did not change (p = 0.6 and 0.8, respectively). After TI there was marked increase in immune activation (CD8+/HLA-DR+/CD38+ cells, 34% (13, 43), p<0.0001) and soluble TNFR2 (1089 ng/L (−189, 1655), p = 0.0008) coinciding with the rebound of HIV viremia.

**Conclusions:**

Our data suggests that interrupting antiretroviral therapy does not reduce cardiovascular disease (CVD) risk, as the improvements in lipid parameters are modest and overshadowed by the decreased HDL levels. Increased immune cell activation and systemic inflammatory responses associated with recrudescent HIV viremia may provide a more cogent explanation for the increased cardiovascular risk associated with treatment interruption and HIV infection.

**Trial Registration:**

ClinicalTrials.gov NCT00015704

## Introduction

The continuous use of antiretroviral therapy (ART) has been associated with a series of metabolic complications [1] that have been linked to increased cardiovascular risk. [2], [3] Treatment associated toxicities together with the costs, both economic and in quality of life terms associated with ART led to the consideration of CD4 driven strategies for the management of HIV infection. [4], [5], [6] Frequently cited arguments in support of those strategies were the potential beneficial metabolic effects of interrupting drug treatment and the possible reduction of drug-related toxicities. However, the strategy is not without risks, including recrudescent HIV viremia and HIV disease progression. The metabolic effects and overall cardiovascular risk of treatment interruption have not been well defined.

An unexpected result of the recently discontinued SMART trial, the largest study that evaluated CD4 driven therapy, was an increase in frequency of cardiovascular events in the drug conservation arm. [7]

Although hyperlipidemia is clearly been linked to the development of atherosclerosis, only recently has the critical role of inflammatory mechanisms in the initiation, progression and acute decompensation of the atheromatous plaque been appreciated. [8], [9], [10]. A wide range of acute and chronic infections have been associated with persistent inflammation which have been hypothesized to accelerate atherosclerosis [11], [12]. Untreated HIV infection is characterized by increased levels of proinflamatory cytokines TNF-alpha[13] and IL-6 and by increased expression of adhesion molecules VCAM-1, ICAM-1 and von Willebrand factor, factors identified to be important in the pathogenesis of atherosclerosis[14]. Fully suppressive ART reverses these abnormalities [14] , and improves endothelial function, independently of the regimen selected. [15]

Antiretroviral treatment interruption (TI) in chronically suppressed individuals represents a unique situation because it links the acute metabolic changes associated with the discontinuation of ART with acute changes in the systemic degree of inflammation associated with reinitiating HIV replication. Individuals who discontinue ART will predictably go from a state of relatively low systemic inflammation (fully suppressed HIV viral replication) to a state of high inflammation, coinciding with the increase in HIV viremia that tends to occur approximately 2 weeks after discontinuation of therapy. [16], [17]

The goal of this analysis was to evaluate the metabolic changes associated with the discontinuation of ART and the changes in immune activation markers to identify changes in cardiovascular risk in this situation. We used the setting of a prospective randomized trial, ACTG 5102, [17] that evaluated the utility of interleukin-2 in delaying the re-initiation of ART as part of a CD4 driven strategy in the management of HIV infected individuals.

## Methods

### Subjects

This study is a sub-study of ACTG study 5102. The protocol for this trial and supporting CONSORT checklist are available as supporting information; see Checklist S1 and Protocol S1. Details summarizing ACTG A5102 have been recently published. [17] ACTG 5102 enrolled HIV-1-infected adults receiving stable ART, who were virologically fully suppressed (<200 HIV RNA copies/mm^3^) for at least three months at the time of enrollment and had a CD4 cell count ≥500 cells/mm^3^. The study was approved by the IRB of all participant institutions and all subjects provided written informed consent.

### Intervention

**Figure 1:**
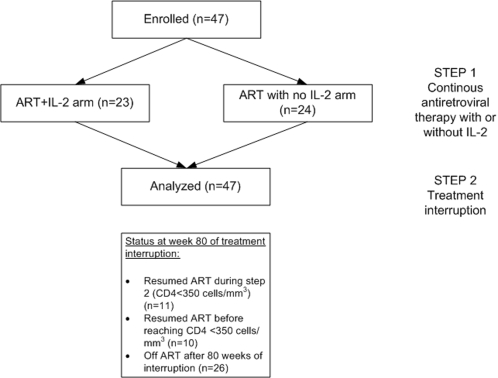
Consort diagram. All patients were included in the analysis and censored after the re-initiation of antiretroviral therapy during Step 2.

47 subjects were then randomly assigned to continue ART for 18 weeks (Step 1) with (ARM A) or without (ARM B) 3 cycles of IL-2 (cycle = 4.5 million IU sc BID x 5 days every 8 weeks). After 18 weeks ART was discontinued in all subjects (Step 2). Subjects were followed monthly with monitoring of their CD4+ T-cell counts and plasma HIV-1 RNA levels. ART was restarted when the CD4+ cells reached a confirmed level of <350 cells/µL. At that point ART was restarted for 6 months and if the CD4+ cells again reached ≥500 cells/µL, the cycle was repeated. In this analysis we report only the results of the first treatment interruption. Figure 1 shows a consort diagram of this study.

### Medications

Recombinant human interleukin-2 (Proleukin®) was provided by Chiron Corporation. ART were provided by prescription through the subject's physician. No changes of antiretroviral regimen were allowed during Step 1. There were protocol defined recommendations for the management of IL-2 toxicities.

### Laboratory

CD4+ and CD8+ T-cell counts were measured using standard flow cytometry techniques at the same local ACTG-certified flow laboratory throughout the study (every 4 weeks during while on ART and at weeks 0,2,4,8, and every 4 weeks thereafter on the treatment interruption phase). HIV viral load was determined at weeks 0, 8 and 16 of Step 1 and weeks 2, 4, 6, 8, 16 and every 8 weeks thereafter in Step 2 using the UltraSensitive Roche Amplicor HIV-1 Monitor™ assay in a central laboratory. Fasting metabolic testing (glucose, insulin, total, calculated LDL and HDL cholesterol and triglycerides) were analyzed at the same time points (weeks 0, 2, 4, 6, 8, 16 and every 8 weeks) at each participating site laboratory. The baseline for Step 1 analysis were the values at randomization into the study. Baseline for changes after treatment interruption were the values obtained immediately before treatment interruption (week 0 of step 2). The number and percentage of CD8+ cells expressing activation markers (CD38+/HLA-DR+) were measured at entry and at week 16 of Step 1 while the participant was receiving ART and at weeks 2, 4, and 8 of Step 2 (TI) using 3 color flow cytometry following ACTG consensus methodology^16^. Serum TNF Receptor alpha 2 was measured before TI and 24 weeks after discontinuation of ART. The metabolic effects of IL-2 had not been described. If there were no changes in the metabolic parameters during the first phase of the study, we planned to combine both arms for the purpose of this analysis.

### Statistical analysis

Summary statistics for continuous variables were presented as median (first quartile, third quartile). Comparison between treatment arms was carried out using a Wilcoxon Rank Sum Test for ordinal valued outcomes, and a Fisher's Exact Test or Pearson's Chi-square Test for categorical factors. Within each arm, a Wilcoxon Signed Rank Test was used to assess differences over time. Spearman's rank correlation was used to measure the relationship between immunologic and metabolic markers. The level of significance used was 0.05. These results are not adjusted for multiple testing and are therefore exploratory. Laboratory results from participants were censored after reinitiating antiretroviral treatment.

## Results

The results of the primary analysis of the study have been recently published. [17] In summary, three cycles of interleukin-2 given during the first 18 weeks of the study, delayed the initial decay of CD4 T cells after TI, but this benefit was lost by 72 weeks. Twenty-one of the study subjects were on a protease inhibitor based regimen.

Baseline and week 16 changes from baseline in fasting total cholesterol, triglycerides, HDL cholesterol and non-HDL cholesterol, fasting glucose and insulin were comparable between the study arms (Table 1). Although there were statistically marginally significant differences observed with respect to week 16 cholesterol and non HDL cholesterol (p = 0.04 and p = 0.03, not corrected for multiple comparisons), within each arm there were no significant changes from baseline. Because of these results the metabolic parameters from subjects in both arms after interruption were combined.

**Table 1:** Summary of changes in lipid and glucose parameters during Step 1 of the trial, when participants were receiving ART with or without IL2.

		ART+IL-2		ART alone		
		Median	(IQR)	Median	IQR	p value
**Cholesterol**	W 0	5.6	(5.4–6.1)	5.3	(4.6–5.7)	0.19
(mmol/L)	W 16	5.8	(5.3–6.5)	5.0	(4.6–5.9)	0.04
**Triglycerides**	W 0	2.4	(1.9–3.3)	1.8	(1.4–2.2)	0.17
(mmol/L)	W 16	2.3	(1.5–3.4)	1.7	(1.3–2.5)	0.29
**HDL**	W 0	1.0	(1.0–1.2)	1.1	(0.9–1.3)	0.45
(mmol/L)	W 16	1.1	(1.0–1.2)	1.0	(0.8–1.3)	0.73
**Non-HDL**	W 0	4.6	(4.2–5.0)	4.1	(3.4–4.5)	0.07
(mmol/L)	W 16	4.8	(4.2–5.6)	3.9	(3.5–4.8)	0.03
**Glucose**	W 0	5.1	(4.6–5.2)	4.9	(4.4–5.2)	0.70
(mmol/L)	W 16	5.0	(4.6–5.5)	5.0	(4.6–5.4)	0.93
**Insulin**	W 0	71	(49–104)	65	(35–90)	0.38
(pmol/L)	W 16	57	(42–110)	61	(35–79)	0.50

The p value represents comparisons between groups (Wilcoxon rank sum). There were no significant differences in the change from baseline within or between groups, so both groups were merged for the Step 2 analysis.

**Figure 2:**
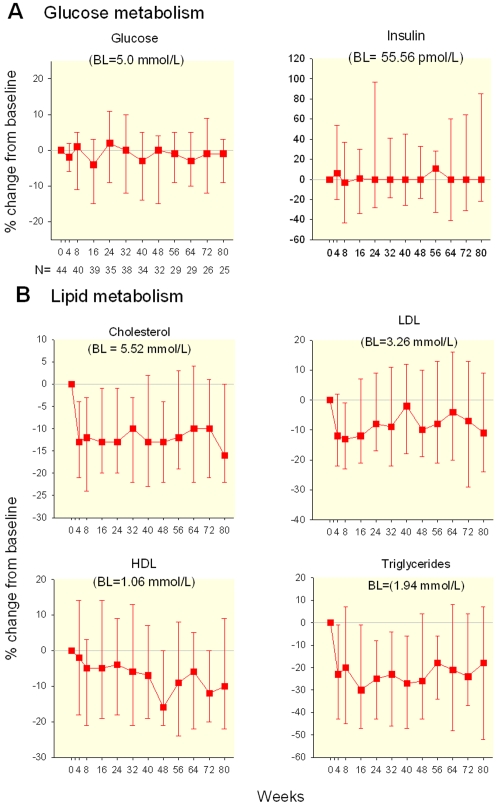
Glucose and Lipid Metabolism During Prolonged TI (median % change from baseline and IQR).

The baseline values for subsequent analysis are the ones immediately before treatment interruption. After the discontinuation of ART there was a rapid decrease of the concentrations of total cholesterol. By week 8 of TI, levels of total cholesterol (TC) decreased (median(Q1, Q3) = −0.73 (−1.19, −0.18) mmol/L, p<0.0001) as well as triglycerides (−0.40 (−0.84, 0.07) mmol/L, p = 0.005). LDL and HDL cholesterol also decreased (−0.36(−0.73,−0.03)mmol/L, p = 0.0007 and −0.05(−0.26,0.03), p = 0.0033, respectively) (Figure 2). These decreases persisted for the duration of the follow up. All of these changes were evident before the return of detectable HIV plasma RNA, defined by HIV-1 RNA >500 copies/mL. Rebounding HIV-1 RNA values >500 copies/mL were identified at 2 weeks in 20 of 45, after 4 weeks in 35 of 45, and for 45 of 45 by week 8 of treatment interruption. This suggests that lipid changes were mediated directly by the discontinuation of ART and not by HIV replication itself. After 48 weeks off therapy there was a continuing decrease in HDL levels and a continuing increase in triglyceride levels.

**Figure 3:**
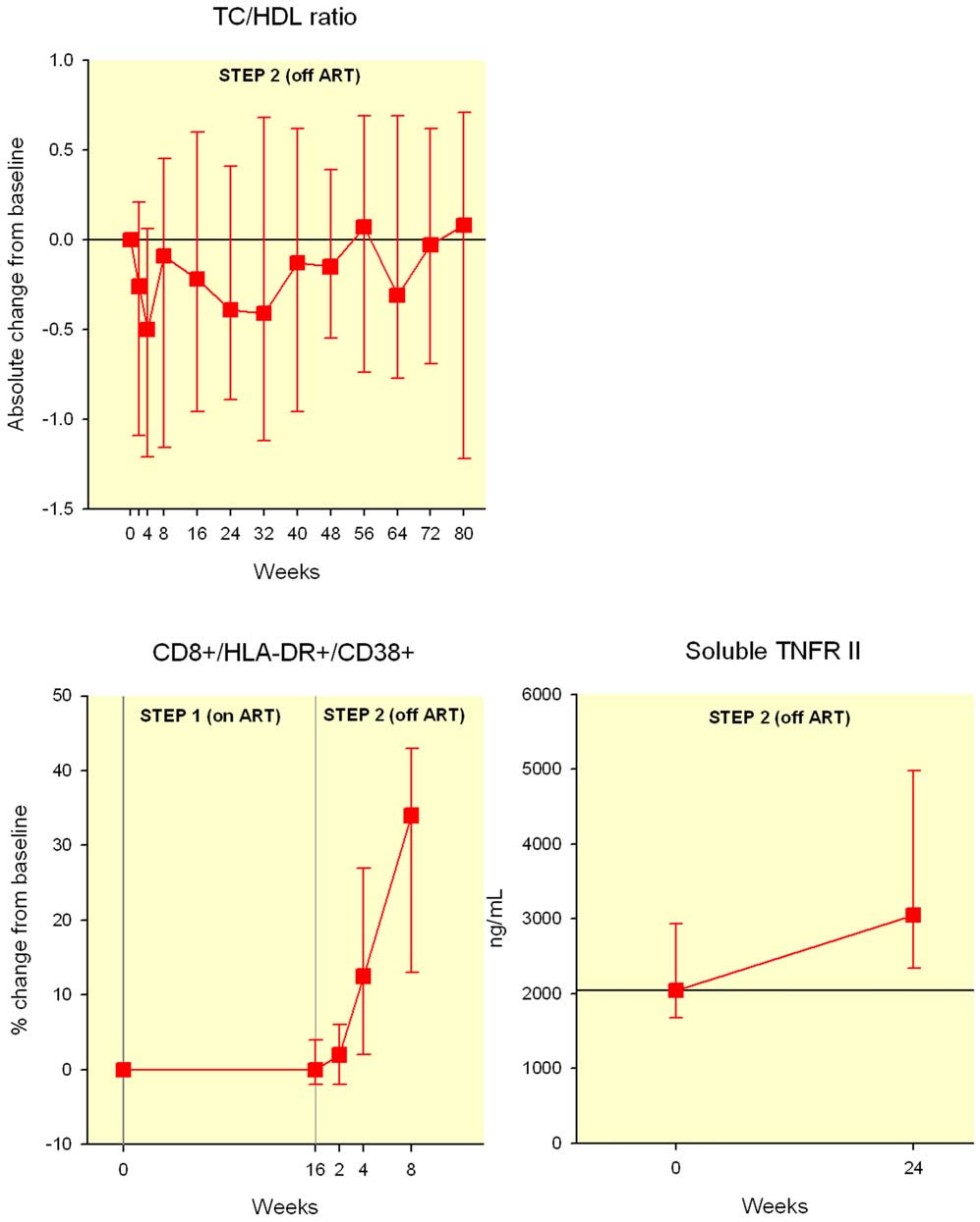
Total cholesterol to HDL ratio changes overtime after treatment interruption (median change and IQR). Percentage change of CD8+/HLA-DR+/CD38+ cells after TI. (median and IQR) Absolute change in soluble serum TNR II levels (median and IQR) Individuals were censored after reinitiating antiretroviral therapy, thus the decreasing N in the figures.

The total cholesterol to HDL cholesterol ratio decreased slightly during the first 4 weeks (−0.5,IQR −1.2–0.06, p = 0.001) after TI but returned to baseline by week 8 (−0.09 (IQR −1.2, 0.5), p = 0.2) and remained unchanged afterwards due to the marked decreases in HDL cholesterol (Figure 3A).

After interruption the percentage of individuals with HDL cholesterol less than 40 mg/mL (1.04 mmol/L) went from 53% before discontinuation to 76% after 48 weeks. After the discontinuation of ART there were no significant changes in either the fasting glucose or insulin levels.

During Step 1 (continuous ART), there was no significant difference within the arms in the change from baseline in the percentage of CD8+ T-cells expressing activation markers (HLA-DR and CD38). The median baseline absolute number of CD8+/HLA DR+ cells were 220 in the IL-2 arm and 204 cells per mm^3^ in the no-IL2 arm, at the end of step 1 they were 267 and 231 per mm^3^ respectively, p = 0.55). There was no significant difference within the arms in the change from baseline in the percentage of CD8+ T-cells expressing activation markers (HLA-DR and CD38). Nevertheless, during Step 1 subjects in the IL-2 group (median change −1%, IQR: −3 to 0%) tended to experience a decrease in the percent of activated CD8+ T-cells while those not receiving IL-2 tended to experience an increase (median change 1%, IQR: −1 to 5%; p = 0.03, Wilcoxon test for the difference between arms).

As expected, after the discontinuation of treatment there was a significant increase in cellular and soluble activation markers in all participants. The increase in CD8+ T-cell activation was faster among individuals that received ART alone during Step 1. These patients experienced a significant increase in activation at week 2 of Step 2 (median increase 3%, vs 0% p = 0.01), while those not receiving IL-2 did not experience a significant increase in activation until week 4 (median increase 20%, p = 0.0002). By eight weeks after ART discontinuation the median increase in activated CD8+ cells was similar between groups (median increase 32% and 42% for IL-2 and non-IL-2 recipients, p = 0.17). After 8 weeks of TI the percentage of activated CD8 cells in the whole group (HLA-DR+ and CD38+ positive) had increased a median of 34% (IQR 13–43%, Wilcoxon signed rank p<0.0001). The levels of TNF receptor alfa II increased approximately by 30% after 24 weeks after the discontinuation of ART (1089 ng/L (−189, 1655), p = 0.0008) (Figure 3).

There were no significant correlations between the changes in lipid or glucose parameters and the changes in immune activation markers or viral markers during the first weeks of follow up (data not shown).

## Discussion

Our study shows the metabolic effects of discontinuing ART. They generally appear to be the reverse pattern of the sequence of changes observed after the initiation of ART. [18] The lack of significant changes in glucose and insulin suggests that the effect of ART on those parameters is limited. That observation is in sharp contrast with the prompt changes in lipid levels seen upon TI. The rapid changes of lipid levels indicates that these effects are due directly to the medications, and most likely are not mediated by changes in body composition which require a lengthier period of time. It is also unlikely that these initial lipoprotein changes are related to HIV replication, as they occurred before the return of HIV viremia.

Over the long term, although the decrease in total and LDL cholesterol might be associated with a decreased cardiovascular risk, those benefits may be offset by the marked decrease in HDL cholesterol observed. Total/HDL cholesterol ratio may more accurately capture the overall cardiac risk as compared to other individual lipoprotein measurements [19]. In fact the total cholesterol to HDL ratio, although improved during the first four weeks after interruption, returned to baseline by 8 weeks. In addition, the initial benefits in triglycerides levels tended to become less apparent as the duration of the TI increased. Our interpretation of these late lipid changes observed after 24–48 weeks of TI is that they represent the adverse metabolic effects of unabated HIV replication rather than any residual effect of ART. These changes are similar to the lipid effects seen in acute and chronic HIV infection in the absence of treatment[20]. The lipid abnormalities could be cytokine driven [21] or mediated directly by HIV replication [22], [23] or both. The net effect of the observed lipid changes would predict little if any improvement in the cardiovascular risk after the discontinuation of ART.

Following treatment interruption there is a prompt and obvious increase in cellular activation, which could negate any potential cardiovascular benefits of TI. The simultaneous combination of these two phenomena (modest decreases in lipids with no net cardiovascular benefit and significant pro-inflammatory changes) could partially explain the increase in cardiovascular events observed in the SMART trial and other studies after the discontinuation of ART. [7], [24] Many patients on ART have been in a state of pro-atherogenic dyslipidemia for years that could have lead to the development of plaques. The sudden change to a more pro-inflammatory state induced by the abrupt return of HIV replication could result in an increased platelet adhesion and inflammatory cell migration into unstable plaque. Many studies have shown that both such local and systemic inflammation plays a central role in the development of atherosclerosis [9].

Our study may also provide some insight explaining two apparently discordant large data sets regarding cardiovascular risk and HIV infection: the one from the Veterans Administration study conducted by Bozzete et al. [25]and the DAD cohort study. [2], [3] In the first study, there was an apparent decreased incidence of cardiovascular events among patients with HIV infection starting therapy in the mid to late 90's. These could be interpreted as improvement in cardiovascular outcomes secondary to the decrease cellular activation associated with the initiation of ART, as the majority of the patients in that cohort were initiating potent ART during the observation period. On the other hand, in the DAD cohort more than 80% of the patients at the time of enrollment (1999–2001) were already receiving potent ART for a median of almost 2 years, with most individuals having undetectable HIV-1 RNA viral loads and a state of low cellular activation. In this state, traditional cardiovascular risk factors including hyperlipidemia become more important and may be responsible for the modest rise in cardiovascular events observed with increased duration of treatment.

Our study has limitations that are important to point out; first it is a pilot study has a relatively small sample size, especially when compared to the more than 5,000 participants in the SMART trial. However our sample has enough power to identify the immediate effects of treatment discontinuation, both in lipid parameters and immune activation, our stated goals. Second the evaluation of immune activation is relatively crude and completed in a number of limited time points; however the results are consistent with what is known about ongoing HIV viremia. The lost to follow up rate was comparable to antiretroviral treatment trials of similar duration, with only 10 participants dropping out of the study (21%).

Our results suggest that TI would not reduce CVD risk, as any improvements in lipid parameters are modest and overshadowed by the decreased HDL levels and the increased immune cell activation due to systemic inflammatory responses associated with returning HIV viremia. These effects could put the patients at a greater cardiovascular risk than when they were receiving antiretroviral treatment. Decreasing immune activation after TI and potentially among patients with ongoing viral replication could mitigate the cardiovascular morbidity and mortality associated with HIV viremia. Prospective trials of anti-inflammatory interventions to mitigate the risk of acute cardiovascular events in cases where treatment must be interrupted are warranted. Treatment interruptions provide a setting in which the relationship between viremia, immune activation and humoral inflammatory markers can be accurately assessed.

## Supporting Information

Checklist S1 CONSORT Checklist
(0.12 MB DOC)

Protocol S1 Trial Protocol
(0.99 MB DOC)

## References

[1] Grinspoon S, Carr A (2005). Cardiovascular risk and body-fat abnormalities in HIV-infected adults.. N Engl J Med.

[2] Friis-Moller N, Sabin CA, Weber R, d'Arminio Monforte A, El-Sadr WM (2003). Combination antiretroviral therapy and the risk of myocardial infarction.. N Engl J Med.

[3] Friis-Moller N, Reiss P, Sabin CA, Weber R, Monforte A (2007). Class of antiretroviral drugs and the risk of myocardial infarction.. N Engl J Med.

[4] Dybul M, Nies-Kraske E, Daucher M, Hertogs K, Hallahan CW (2003). Long-cycle structured intermittent versus continuous highly active antiretroviral therapy for the treatment of chronic infection with human immunodeficiency virus: effects on drug toxicity and on immunologic and virologic parameters.. J Infect Dis.

[5] Dybul M, Nies-Kraske E, Dewar R, Maldarelli F, Hallahan CW (2004). A proof-of-concept study of short-cycle intermittent antiretroviral therapy with a once-daily regimen of didanosine, lamivudine, and efavirenz for the treatment of chronic HIV infection.. J Infect Dis.

[6] Havlir DV (2002). Structured intermittent treatment for HIV disease: Necessary concession or premature compromise?. Proc Natl Acad Sci U S A.

[7] El-Sadr W, Neaton J (2006). Episodic CD4-Guided Use of ART Is Inferior to Continuous Therapy: Results of the SMART Study. 13th Conference on Retrovirus and Opportunistic Infections..

[8] Libby P (2002). Inflammation in atherosclerosis.. Nature.

[9] Libby P, Ridker PM, Maseri A (2002). Inflammation and atherosclerosis.. Circulation.

[10] Hansson GK, Libby P (2006). The immune response in atherosclerosis: a double-edged sword.. Nature Reviews Immunology.

[11] Libby P, Egan D, Skarlatos S (1997). Roles of infectious agents in atherosclerosis and restenosis: an assessment of the evidence and need for future research.. Circulation.

[12] Danesh J, Collins R, Peto R (1997). Chronic infections and coronary heart disease: is there a link?. Lancet.

[13] Hober D, Haque A, Wattre P, Beaucaire G, Mouton Y (1989). Production of tumour necrosis factor-alpha (TNF-alpha) and interleukin-1 (IL-1) in patients with AIDS. Enhanced level of TNF-alpha is related to a higher cytotoxic activity.. Clin Exp Immunol.

[14] Wolf K, Tsakiris DA, Weber R, Erb P, Battegay M (2002). Antiretroviral therapy reduces markers of endothelial and coagulation activation in patients infected with human immunodeficiency virus type 1.. J Infect Dis.

[15] Stein JH, Cotter BR, Parker RA (2005). Antiretroviral therapy improves endothelial function in individuals with human immunodeficiency virus infection: a prospective, randomized multicenter trial (adult AIDS clinical trials group study A5152s) (abstract).. Circulation;.

[16] Davey RT, Bhat N, Yoder C, Chun TW, Metcalf JA (1999). HIV-1 and T cell dynamics after interruption of highly active antiretroviral therapy (HAART) in patients with a history of sustained viral suppression.. Proc Natl Acad Sci U S A.

[17] Henry K, Katzenstein D, Cherng DW, Valdez H, Powderly W (2006). A pilot study evaluating time to CD4 T-cell count <350 cells/mm(3) after treatment interruption following antiretroviral therapy +/− interleukin 2: results of ACTG A5102.. J Acquir Immune Defic Syndr.

[18] Dube MP, Parker RA, Tebas P, Grinspoon SK, Zackin RA (2005). Glucose metabolism, lipid, and body fat changes in antiretroviral-naive subjects randomized to nelfinavir or efavirenz plus dual nucleosides.. Aids.

[19] Stampfer MJ, Sacks FM, Salvini S, Willett WC, Hennekens CH (1991). A Prospective-Study of Cholesterol, Apolipoproteins, and the Risk of Myocardial-Infarction.. New England Journal of Medicine.

[20] Riddler SA, Smit E, Cole SR, Li R, Chmiel JS (2003). Impact of HIV infection and HAART on serum lipids in men.. Jama.

[21] Yudkin JS, Kumari M, Humphries SE, Mohamed-Ali V (2000). Inflammation, obesity, stress and coronary heart disease: is interleukin-6 the link?. Atherosclerosis.

[22] Mujawar Z, Rose H, Morrow MP, Pushkarsky T, Dubrovsky L (2006). Human immunodeficiency virus impairs reverse cholesterol transport from macrophages.. PLoS Biol.

[23] Carr A, Ory D (2006). Does HIV cause cardiovascular disease?. PLoS Med.

[24] Skiest D, Havlir D, Coombs R, Adams E, Cain P (2006). Predictors of HIV Disease Progression in Patients Who Stop ART with CD4 Cell Counts >350 cells/mm3. 13th Conference on Retrovirus and Opportunistic Infections..

[25] Bozzette SA, Ake CF, Tam HK, Chang SW, Louis TA (2003). Cardiovascular and cerebrovascular events in patients treated for human immunodeficiency virus infection.. N Engl J Med.

