# Supplementary material for: Metabolic and Immune Activation Effects of Treatment Interruption in Chronic HIV-1 Infection: Implications for Cardiovascular Risk
Source: PLoS One. 2008 Apr 23;3(4):e2021. doi: 10.1371/journal.pone.0002021 (PMC2292263; doi:10.1371/journal.pone.0002021)
Supplement: Protocol S1 — Trial Protocol (0.99 MB DOC) [file pone.0002021.s002.doc]

**A5102**

**AN OPEN-LABEL, PILOT STUDY UTILIZING CD4+ T-CELL COUNTS < 350 CELLS/MM3 AS THE THRESHOLD FOR RESTARTING THERAPY WITH POTENT ANTIRETROVIRAL THERAPY +/- INTERLEUKIN-2 TO DETERMINE THE EFFECT OF PULSE THERAPY ON THE CHARACTERISTICS OF TREATMENT INTERRUPTIONS**

**A Multicenter Trial of the Adult AIDS Clinical Trials Group (AACTG)**

**Sponsored by:**

**The National Institute of Allergy and Infectious Diseases**

**Pharmaceutical Support Provided by:**

**Chiron Corporation**

**BB IND # 7250**

**The AACTG Immunology Research Agenda Committee: Jeffrey M. Jacobson, M.D., Chair**

**Protocol Chair: W. Keith Henry, M.D.**

**Protocol Vice Chairs: Pablo Tebas, M.D.**

**William Powderly, M.D.**

**DAIDS Clinical Representative:** **Lawrence Fox, M.D., Ph.D.**

**Clinical Trials Specialists: Nasreen Jahed, M.P.H.**

**Michelle Blanchard Vargas, M.A.**

**FINAL Version 2.0**

**March 5, 2004**

TABLE OF CONTENTS

Page

SITES PARTICIPATING IN A5102 [5](#__RefHeading___Toc61340670)

SITES PARTICIPATING IN A5109s [5](#__RefHeading___Toc61340671)

PROTOCOL TEAM ROSTER [6](#__RefHeading___Toc61340672)

STUDY MANAGEMENT [10](#__RefHeading___Toc61340673)

SCHEMA [12](#__RefHeading___Toc61340674)

SUBSTUDY SCHEMA [17](#__RefHeading___Toc61340675)

1.0 STUDY OBJECTIVES [18](#__RefHeading___Toc61340676)

1.1 Primary Objective [18](#__RefHeading___Toc61340677)

1.2 Secondary Objectives [18](#__RefHeading___Toc61340678)

1.3 A5109s Substudy Primary Objective [19](#__RefHeading___Toc61340679)

1.4 A5109s Substudy Secondary Objectives [19](#__RefHeading___Toc61340680)

2.0 INTRODUCTION [20](#__RefHeading___Toc61340681)

2.1 Background [20](#__RefHeading___Toc61340682)

2.2 Rationale [23](#__RefHeading___Toc61340683)

2.3 Substudy Background and Rationale [30](#__RefHeading___Toc61340684)

3.0 STUDY DESIGN [31](#__RefHeading___Toc61340685)

4.0 SELECTION AND ENROLLMENT OF SUBJECTS [33](#__RefHeading___Toc61340686)

4.1 Inclusion Criteria – Protocol Entry/Step 1 [33](#__RefHeading___Toc61340687)

4.2 Exclusion Criteria [36](#__RefHeading___Toc61340688)

4.3 Inclusion Criteria – Steps 2 through 8 [38](#__RefHeading___Toc61340689)

4.4 Inclusion Criteria – A5109s [40](#__RefHeading___Toc61340690)

4.5 Study Enrollment Procedures [40](#__RefHeading___Toc61340691)

4.6 Substudy Enrollment Procedures [41](#__RefHeading___Toc61340692)

4.7 Coenrollment Guidelines [41](#__RefHeading___Toc61340693)

5.0 TREATMENT [41](#__RefHeading___Toc61340694)

5.1 Regimens, Duration, and Administration [41](#__RefHeading___Toc61340695)

5.2 IL-2 Product Formulation [44](#__RefHeading___Toc61340696)

5.3 Product Supply, Distribution, and Pharmacy [45](#__RefHeading___Toc61340697)

5.4 Concomitant Medications [45](#__RefHeading___Toc61340698)

5.5 Adherence Assessment [47](#__RefHeading___Toc61340699)

6.0 CLINICAL AND LABORATORY EVALUATIONS [47](#__RefHeading___Toc61340700)

6.1 Schedule of Events [48](#__RefHeading___Toc61340701)

6.2 Substudy Schedule of Events [55](#__RefHeading___Toc61340702)

6.3 Definitions for Schedule of Events – Timing of Evaluations [55](#__RefHeading___Toc61340703)

6.4 Special Instructions and Definitions of Evaluations [57](#__RefHeading___Toc61340704)

6.5 Off-Drug Requirements [66](#__RefHeading___Toc61340705)

7.0 TOXICITY MANAGEMENT [67](#__RefHeading___Toc61340706)

7.1 Symptom Specific Management [67](#__RefHeading___Toc61340707)

7.2 Antiretroviral Drugs [69](#__RefHeading___Toc61340708)

7.3 IL-2 Dose Modifications [70](#__RefHeading___Toc61340709)

8.0 CRITERIA FOR STUDY AND TREATMENT DISCONTINUATION [72](#__RefHeading___Toc61340710)

8.1 Premature and Permanent Study Discontinuation [72](#__RefHeading___Toc61340711)

8.2 Premature and Permanent IL-2 Discontinuation (Arm A Only) [73](#__RefHeading___Toc61340712)

9.0 STATISTICAL CONSIDERATIONS [73](#__RefHeading___Toc61340713)

9.1 General Design Issues [73](#__RefHeading___Toc61340714)

9.2 Endpoints [74](#__RefHeading___Toc61340715)

9.3 Substudy Endpoints [75](#__RefHeading___Toc61340716)

9.4 Sample Size and Accrual [76](#__RefHeading___Toc61340717)

9.5 Substudy Sample Size and Accrual [77](#__RefHeading___Toc61340718)

9.6 Analysis [77](#__RefHeading___Toc61340719)

10.0 COLLECTION AND MONITORING AND ADVERSE EXPERIENCE REPORTING [82](#__RefHeading___Toc61340720)

10.1 Records to Be Kept [82](#__RefHeading___Toc61340721)

10.2 Role of Data Management [82](#__RefHeading___Toc61340722)

10.3 Clinical Site Monitoring and Record Availability [82](#__RefHeading___Toc61340723)

10.4 Serious Adverse Experience (SAE) Reporting [83](#__RefHeading___Toc61340724)

11.0 HUMAN SUBJECTS [83](#__RefHeading___Toc61340725)

11.1 Institutional Review Board (IRB) Review and Informed Consent [83](#__RefHeading___Toc61340726)

11.2 Subject Confidentiality [83](#__RefHeading___Toc61340727)

11.3 Study Discontinuation [83](#__RefHeading___Toc61340728)

12.0 PUBLICATION OF RESEARCH FINDINGS [84](#__RefHeading___Toc61340729)

13.0 BIOHAZARD CONTAINMENT [84](#__RefHeading___Toc61340730)

14.0 REFERENCES [85](#__RefHeading___Toc61340731)

APPENDIX I: IMMUNOLOGIC TESTING [94](#__RefHeading___Toc61340732)

APPENDIX II: SPECIMEN PROCESSING OF PLASMA FOR HIV-1 RNA AND FOR FUTURE VIROLOGIC DETERMINATIONS [97](#__RefHeading___Toc61340733)

APPENDIX III: VIROLOGIC AND RESISTANCE TESTING [98](#__RefHeading___Toc61340734)

APPENDIX IV: VIRAL CULTURE USING THE QUANTITATIVE PBMC MICRO CULTURE ASSAY [101](#__RefHeading___Toc61340735)

APPENDIX V: SPECIMEN PROCESSING FOR PBMCs, PLASMA, SERUM AND URINE [102](#__RefHeading___Toc61340736)

APPENDIX VI: A5109s ASSAYS [104](#__RefHeading___Toc61340737)

APPENDIX VII: SAMPLE INFORMED CONSENT

# SITES PARTICIPATING IN A5102

A5102: An Open-Label, Pilot Study Utilizing CD4+ T-Cell Counts < 350 Cells/mm3 As The Threshold For Restarting Therapy With Potent Antiretroviral Therapy +/- Interleukin-2 To Determine The Effect Of Pulse Therapy On The Characteristics Of Treatment Interruptions

A5102 is a multicenter trial open to all sites.

# SITES PARTICIPATING IN A5109s

A5109s: The Effect of Viral Replication on Antigen-Specific Proliferation Versus Cytokine Production in Subjects Undergoing Pulse Therapy: A Substudy of A5102

A5109s is a limited site substudy. The following sites and their subunits have been approved to participate:

Case Western Reserve University

**Duke University Medical Center**

**Northwestern University**

University of North Carolina at Chapel Hill

# PROTOCOL TEAM ROSTER

Protocol Chair

W. Keith Henry, M.D.

**HIV Program**

**Hennepin County Medical Center**

University of Minnesota

**701 Park Ave mailstop # 834**

**Minneapolis**, MN 55**415**

Phone: (6**12**) **873-7516**

Fax: (6**12**) **630-8290**

E-mail: [**keithh6680@aol.com**](mailto:keithh6680@aol.com)

Vice Chairs

Pablo Tebas, M.D.

Washington University School of Medicine

4511 Forestpark, Suite 304

St. Louis, MO 63108

Phone: (314) 454-0058

Fax: (314) 361-5231

E-mail: [ptebas@im.wustl.edu](mailto:ptebas@imgate.wustl.edu)

William Powderly, M.D.

Division of Infectious Disease

Washington University School of Medicine

660 South Euclid, Box 8051

St. Louis, MO 63110

Phone: (314) 454-8214

Fax: (314) 454-5392

E-mail: [wpowderl@imgate.wustl.edu](mailto:wpowderl@imgate.wustl.edu)

DAIDS Clinical Representative

Lawrence Fox, M.D., Ph.D.

HIVRB/TRP/DAIDS/NIAID/NIH

**Room 5104**

**6700-B Rockledge Drive**, MSC 7624 MSC

Bethesda, MD 20892-7624

**Phone: (301) 402-0129**

Fax: (301) 435-9282

E-mail: [**lfox**@niaid.nih.gov](mailto:lfox@niaid.nih.gov)

Clinical Trials Specialist**s**

Nasreen Jahed, M.P.H.

**AACTG Operations Center**

**8757 Georgia Avenue, 12th Floor**

**Silver Spring, MD 20910**

**Phone: (301) 628-3374**

**Fax: (301) 628-3302**

E-mail: [njahed@s-3.com](mailto:njahed@s-3.com)

Michelle Blanchard Vargas, M.A.

AACTG Operations Center

**8757 Georgia Avenue, 12th Floor**

**Silver Spring, MD 20910**

**Phone: (301) 628-3364**

**Fax: (301) 628-3302**

E-mail: [mblanchardvargas@s-3.com](mailto:mblanchardvargas@s-3.com)

Statistician

Deborah X. Weng Cherng, M.S.

Senior Biostatistician – Immunology

Department of Biostatistics

Statistical and Data Analysis Center

FXB Building, Room 533A

651 Huntington Avenue

Boston, MA 02115

Phone: (617) 432-1113

Fax: (617) 432-2832

E-mail: [weng.xiaohui@fstrf.org](mailto:weng.xiaohui@fstrf.org)

Data Manager

Laurie S. Myers, M.S., M.T. (ASCP)

Frontier Science and Technology

Research Foundation, Inc.

4033 Maple Road

Amherst, NY 14226-1056

Phone: (716) 834-0900 x338

Fax: (716) 834-8432

E-mail: [myers.laurie@fstrf.org](mailto:myers.laurie@fstrf.org)

DAIDS Pharmacist

Elaine Ferguson, R.Ph., M.S.

PAB, DAIDS, NIAID, NIH

6700-B Rockledge Drive

Room 5239, MSC 7620

Bethesda, MD 20892-7620

Phone: (301) 435-3742

Fax: (301) 402-1506

E-mail: [eferguson@niaid.nih.gov](mailto:eferguson@niaid.nih.gov)

Immunologist

John L. Schmitz, Ph.D.

Department of Pathology and Laboratory Medicine

University of North Carolina at Chapel Hill

East Wing, Room 1035

101 Manning Drive

Chapel Hill, NC 27514

Phone: (919) 966-8453

Fax: (919) 966-0486

E-mail: [jschmitz@unch.unc.edu](mailto:jschmitz@unch.unc.edu)

Virologist

David Katzenstein, M.D.

Division of Infectious Disease

Stanford University Medical Center

300 Pasteur Drive, S156

Stanford, CA 94305-5107

Phone: (650) 725-8304

Fax: (650) 725-2395

E-mail: [davidkk@Leland.Stanford.edu](mailto:davidkk@Leland.Stanford.edu)

Field Representative

Ann Conrad, R.N.

**MetroHealth Medical Center**

**2500 MetroHealth Drive**

**CCP-2001**

**Cleveland, Ohio 44109**

Phone: **(216) 778-5489**

Fax: **(216) 778-3019**

E-mail: [**aconrad@metrohealth.org**](mailto:conrad.ann@clevelandactu.org)

Laboratory Technologist

Carol T. Schnizlein-Bick, Ph.D.

Department of Medicine/Infectious Diseases

Indiana University School of Medicine

1001 West 10th Street, OPW 430

Indianapolis, IN 46202-2879

Phone: (317) 630-6971

Fax: (317) 630-7522

Email: [cschnizl@iupui.edu](mailto:cschnizl@iupui.edu)

CCG Representative

Jo Anne Bennett, R.N., Ph.D.

CAB Liaison

Mount Sinai Medical Center

One Jane Street, 6A

New York, NY 10014

Phone: (212) 442-3381

Fax: (212) 442-3535

E-mail: [jbennett@dohlan.cn.ci.nyc.ny.us](mailto:jbennett@dohlan.cn.ci.nyc.ny.us)

HIV RAC Representative

John Alexander Bartlett, M.D.

Division of Infectious Diseases

Duke University Medical Center

Clinic 2J, Hospital South

Trent Drive, Box 3284

Durham, NC 27710

Phone: (919) 684-6416

Fax: (919) 681-8474

E-mail: [jab5@acpub.duke.edu](mailto:jab5@acpub.duke.edu)

Pharmaceutical Representative

Anne-Marie Duliege, M.D., M.S.

Senior Medical Director

Clinical Development

Chiron Corporation

4560 Horton Street, MS U-140

Emeryville, CA 94608

Phone: (510) 923-2715

Fax: (510) 923-3450

E-mail: [anne-marie_duliege@cc.chiron.com](mailto:anne-marie_duliege@cc.chiron.com)

Laboratory Data Coordinator

**Jennifer Nowak**

Laboratory Data Coordinator

Frontier Science and Technology

Research Foundation, Inc.

4033 Maple Road

Amherst, NY 14226-1056

Phone: **716-834-0900 x324**

Fax: (716) 834-8432

E-mail: [**nowak.jennifer@fstrf.org**](mailto:schiffhauer.joanne@fstrf.org)

Investigators

David W. Haas, M.D.

Infectious Disease

Vanderbilt University Medical Center

345 24th Avenue North, Suite 105

Nashville, TN 37203

Phone: (615) 467-0154

Fax: (615) 467-0159

E-mail: [david.w.haas@vanderbilt.edu](mailto:david.w.haas@vanderbilt.edu)

Michael Lederman, M.D.

Division of Infectious Disease

Department of Medicine

Case Western Reserve University

Room 301B Foley Building

2061 Cornell Road

Cleveland, OH 44106-5083

Phone: (216) 844-8786

Fax: (216) 844-5523

E-mail: [lederman.michael@clevelandactu.org](mailto:lederman.michael@clevelandactu.org)

**Ronald Reisler, MD, MPH**

**Institute of Human Virology**

**University of Maryland Medical Center**

**725 West Lombard Street**

**Room N556**

**Baltimore, MD 21201**

**Phone: (410) 706-4637**

**Fax: (410) 706-1992**

**email:** [**reisler@umbi.umd.edu**](mailto:reisler@umbi.umd.edu)

Investigators (Cont’d)

Hernan Valdez, M.D.

**Associate Director Virology**

**Boehringer-Ingelheim**

**Pharmaceuticals Inc.**

**900 Ridgebury Road**

**P.O. Box 368**

**Ridgebury, CT 06877**

**Phone: (203) 778-7909**

**Fax: (203) 798-5433**

**E-mail:**

[**hvaldez@rdg.boehringer-ingelheim.com**](mailto:hvaldez@rdg.boehringer-ingelheim.com)

# STUDY MANAGEMENT

All questions concerning this protocol should be sent via e-mail to [actg.teamA5102@fstrf.org](mailto:actg.teamA5102@fstrf.org). The appropriate team member will respond via e-mail with a “cc” to [actg.teamA5102@fstrf.org](mailto:actg.teamA5102@fstrf.org). A response should generally be received within 24 hours (Monday-Friday).

Protocol e-mail group: Sites registering to this study should contact the Computer Support Group at the Data Management Center via e-mail ([actg.user.support@fstrf.org](mailto:actg.user.support@fstrf.org)) to have the relevant personnel at the site added to the actg.protA5102 e-mail group as soon as possible. Inclusion in the protocol e-mail group will ensure that sites receive important information about the study during its implementation and conduct.

FOR QUESTIONS RELATED TO CLINICAL MEDICAL MANAGEMENT, INCLUDING ENTRY CRITERIA, TOXICITY MANAGEMENT, CONCOMITANT MEDICATIONS, AND CO-ENROLLMENT, THE PROTOCOL CHAIR/PROTOCOL VICE CHAIRS WILL RESPOND:

- Send an e-mail message to [actg.teamA5102@fstrf.org](mailto:actg.teamA5102@fstrf.org) (ATTN: W. Keith Henry, M.D., Pablo Tebas, M.D., and William Powderly, M.D.).
- Include the protocol #, PID, SID, and a brief relevant history.

**FOR QUESTIONS SPECIFICALLY RELATED TO IMMUNOLOGIC OR VIROLOGIC LABORATORY TESTS, THE PROTOCOL IMMUNOLOGIST OR VIROLOGIST WILL RESPOND:**

- Send an e-mail message to [actg.teamA5102@fstrf.org](mailto:actg.teamA5102@fstrf.org) (ATTN: David Katzenstein, M.D. (Virology) and/or John Schmitz, Ph.D. (Immunology).

**FOR NON CLINICAL QUESTIONS ABOUT INCLUSION/EXCLUSION CRITERIA, THE SCHEDULE OF EVENTS, CASE REPORT FORMS, RANDOMIZATION/REGISTRATION, TRANSFERS, DELINQUENCIES, AND OTHER DATA MANAGEMENT ISSUES, THE DATA MANAGER WILL RESPOND:**

- Send an e-mail message to [actg.teamA5102@fstrf.org](mailto:actg.teamA5102@fstrf.org) (ATTN: Laurie Myers).
- Include the protocol #, PID, SID, and a detailed description of the question.

**FOR RANDOMIZATION QUESTIONS OR PROBLEMS AND SID LISTS, THE SDAC/DMC PROGRAMMERS WILL RESPOND:**

- Call the SDAC/DMC Randomization Desk at (716) 834-0900, x301 or e-mail sdac.random.desk.

**FOR COMPUTER AND SCREEN PROBLEMS, THE SDAC/DMC PROGRAMMERS WILL RESPOND:**

- Send an e-mail message to [actg.user.support@fstrf.org](mailto:actg.user.support@fstrf.org).

**FOR PROTOCOL QUESTIONS, THE CLINICAL TRIALS SPECIALISTS WILL RESPOND:**

- Send an e-mail message to [actg.teamA5102@fstrf.org](mailto:actg.teamA5102@fstrf.org) (ATTN: **Nasreen Jahed and** Michelle Blanchard Vargas).

FOR REQUESTS FOR COPIES OF THE PROTOCOL:

- Hard copies: Send an e-mail message to [ACTG.OPS@fstrf.org](mailto:ACTG.OPS@fstrf.org) (ATTN: Diane Delgado).
- Electronic copies can be downloaded from the Members area of the AACTG Web site ([http://aactg.s-3.com](http://aactg.s-3.com/)).

FOR PROTOCOL REGISTRATION:

- Send an e-mail message to [**PROTOCOL@tech-res.com**](mailto:PROTOCOL@tech-res.com)**, or**
- **Call (301) 897-1707.**

FOR QUESTIONS OR PROBLEMS REGARDING STUDY DRUG, DOSE, SUPPLIES, RECORDS, AND RETURNS:

- Call Elaine Ferguson, Protocol Pharmacist, at (301) 496-8213.

FOR IND #:

Contact the DAIDS Regulatory Compliance Center

- **Send an e-mail message to** [**Regulatory@tech-res.com**](mailto:Regulatory@tech-res.com)**, or**
- Call **(301) 897-1706**.

TO ORDER STUDY DRUG:

- Call the NIAID Clinical Research Products Management Center at (301) 294-0741.

FOR SERIOUS ADVERSE EXPERIENCES (SAE) QUESTIONS:

Contact the DAIDS Regulatory Compliance Center Safety Office

- Send an e-mail message to [**SAE@tech-res.com**](http://rcc.tech-res-intl.com/), or
- Call 1-800-537-9979.

Any phone calls must be documented by e-mail to [actg.teamA5102@fstrf.org](mailto:actg.teamA5102@fstrf.org). This will be the site’s responsibility.

Additional information concerning management of AACTG studies can be found on the AACTG Web page.

# SCHEMA

A5102: AN OPEN-LABEL, PILOT STUDY UTILIZING CD4+ T-CELL COUNTS < 350 CELLS/MM3 AS THE THRESHOLD FOR RESTARTING THERAPY WITH POTENT ANTIRETROVIRAL THERAPY +/- INTERLEUKIN-2 TO DETERMINE THE EFFECT OF PULSE THERAPY ON THE CHARACTERISTICS OF TREATMENT INTERRUPTIONS

DESIGN A5102 is a pilot, multicenter, multistep, open-label study randomizing subjects to +/- interleukin-2 (IL-2). The study is designed to evaluate the intercept and slope of CD4+ T-cell counts during Step 2 (treatment interruption). Potent antiretroviral therapy (ART) will be initiated and interrupted, based on CD4+ T-cell counts, as an alternative approach to the chronic management of HIV infection.

SAMPLE SIZE

and POPULATION 80 subjects (40 per arm)

Male and female HIV-1 infected subjects who are at least 18 years of age and who have been on a stable, potent ART regimen for **at least** 3 months **prior to entry**. **Subjects must have a** plasma HIV-1 RNA level < 400 copies/mL **for > 6 months prior to the screening visit to include the** screening RNA level, **a plasma HIV-1 RNA** level < 200 copies/mL at preentry and a CD4+ T-cell count ≥ 500 cells/mm3 at screening. Subjects must not have a prior history of IL-2 use.

DURATION This study will last approximately 196 weeks (4 years).

STRATIFICATION Subjects will be stratified at randomization based on their lifetime documented CD4+ cell count nadir, when available. There will be no sample size limitation for each stratum. The three strata are:

- Stratum 1: Nadir ≤ 200 CD4+ cells/mm3
- Stratum 2: Nadir > 200 CD4+ cells/mm3
- Stratum 3: No documented nadir available

REGIMEN Subjects entering the study will be randomly assigned to either of the following arms:

- Arm A: Pulses of potent ART + cycles of IL-2

There is a maximum of nine cycles of IL-2 for Arm A:

Step 1: IL-2 cycles 1, 2, and 3

Step 4: IL-2 cycles 4, 5, and 6

Step 7: IL-2 cycles 7, 8, and 9

- Arm B: Pulses of potent ART alone

Subjects must provide their own potent ART for the duration of the study. Potent ART is currently defined as three or more antiretroviral agents or dual protease inhibitors. No restrictions will be placed on the potent antiretroviral drug combinations. However, it is strongly recommended that they involve FDA approved medications used in accordance with the most recent Department of Health and Human Services’ (DHHS) treatment guidelines on potent ART available on the Web at [**http://www.aidsinfo.nih.gov**](http://www.aidsinfo.nih.gov/).

IL-2 will be administered as rhIL-2, 4.5 MIU by subcutaneous injection twice a day for 5 days every 8 weeks for three cycles during Steps 1, 4, and 7 for subjects who are randomized to Arm A.

This study has **eight** steps. After randomization to Step 1, subjects must register to each successive step **(Step 2 through Step 7)** within 6 weeks after completing the evaluations on the last visit of the previous step. **There is no time limitation for subjects to register to Step 8.** For Steps 2 and 5, treatment interruption is defined as the withdrawal of both potent ART and IL-2 therapy.

Step 1: Potent ART + Cycles of IL-2 Versus Potent ART Alone

Subjects will begin Step 1 if their CD4+ T-cell count is ≥ 500 cells/mm3 at screening, and if their plasma HIV-1 RNA is < 400 copies/mL at screening and < 200 copies/ mL at preentry. Subjects will be randomized to either potent ART and IL-2 therapy or potent ART alone. Subjects in Arm A will receive potent ART for approximately 17 weeks in addition to three cycles of IL-2 while subjects in Arm B will receive potent ART alone.

Step 2: Treatment Interruption (No IL-2 and No Potent ART)

Subjects will be eligible for Step 2 if their CD4+ T-cell count is ≥ 500 cells/mm3 at the latest of either 1) week 17 of Step 1 or 2) at the visit immediately following IL-2 cycle #3 (in the event of a delayed cycle) or 3) at the visit in which it is determined that IL-2 cycle #3 will be skipped. **Subjects who fail** to maintain a CD4+ T-cell count ≥ 500 cells/mm3 at the conclusion of Step 1 will **either register to Step 8 or be** taken off study. Upon registration to Step 2, subjects in Arm A will stop both potent ART and IL-2 therapy, and subjects in Arm B will stop potent ART. All subjects will have their CD4+ T cell count measured every 4 weeks until step completion. **Subjects who resume potent ART while on Step 2 can either register to Step 8 or be taken off study.**

Step 3: Potent ART Alone

Subjects will be eligible to register to Step 3 when the CD4+ T-cell count is < 350 cells/mm3 on two successive determinations during Step 2. In the event of a missed visit, the two successive CD4 draws cannot be > 8 weeks apart. Once subjects register to Step 3, only potent ART will be restarted. Subjects will remain on potent ART for 24 weeks.

Step 4: Potent ART + Cycles of IL-2 Versus Potent ART Alone

Subjects will be eligible to register to Step 4 if the HIV-1 RNA level is < 200 copies/mL at +24 weeks after starting Step 3. Only the RNA value at week +24 of Step 3 will be used to determine eligibility for Step 4 even if the subject has an HIV-1 RNA value < 200 copies/mL at an earlier time during Step 3. If the subject’s RNA level is > 200 copies/mL at week +24 of Step 3, the subject **can either register to Step 8 or** be taken off study. During Step 4, subjects will continue to receive potent ART with or without IL-2 for approximately another 17 weeks according to their original treatment assignment.

Step 5: Treatment Interruption (No IL-2 and No Potent ART)

Subjects will be eligible for Step 5 if their CD4+ T-cell count is ≥ 500 cells/mm3 at the latest of either 1) week +17 of Step 4 or 2) at the visit immediately following IL-2 cycle #6 (in the event of a delayed cycle) or 3) at the visit in which it is determined that IL-2 cycle #6 will be skipped. **If subjects fail** to achieve a CD4+ T-cell count ≥ 500 cells/mm3 at the conclusion of Step 4**,** the subject **can either register to Step 8 or be** taken off study. Upon registration to Step 5, subjects in Arm A will stop both potent ART and IL-2 therapy, and subjects in Arm B will stop potent ART. All subjects will have their CD4+ T-cell count measured every 4 weeks until step completion. **Subjects who resume potent ART while on Step 5 can either register to Step 8 or be taken off study.**

Step 6: Potent ART Alone

Subjects will be eligible to register to Step 6 when the CD4+ T-cell count is < 350 cells/mm3 on two successive determinations during Step 5. In the event of a missed visit, the two successive CD4 draws cannot be > 8 weeks apart. Once subjects register to Step 6, only potent ART will be restarted. Subjects will remain on potent ART for 24 weeks.

Step 7: Potent ART + Cycles of IL-2 Versus Potent ART Alone

Subjects will be eligible to register to Step 7 if their HIV-1 RNA level is < 200 copies/mL at +24 weeks after starting Step 6. Only the HIV-1 RNA value at week +24 of Step 6 will be used to determine eligibility to Step 7 even if the subject has an RNA value < 200 copies/mL at an earlier time during Step 6. If the subject’s

RNA level is > 200 copies/mL at week +24 of Step 6, the subject **can either register to Step 8 or** will be taken off study. During Step 7, subjects will continue to receive potent ART with or without IL-2 for approximately another 17 weeks according to their original treatment assignment. **Subjects who complete Step 7 will not continue on to Step 8 but rather will complete the study termination evaluations as outlined in section 6.1.**

**Step 8: Observation**

Subjects will be eligible to register to Step 8 if they either have failed the eligibility criteria to the next sequential step (Steps 1-7), do not want to follow the study schema or they are advised by the primary care physician not to follow the study schema and they agree to being followed. During Step 8, subjects will be required to have laboratory and clinical evaluations performed every 6 months until the study is closed. The study will close when the last subject has completed following the protocol schema (Step 1 through Step 7).

| STEP 1  Yes: Continue to Step 2  No: Go to **Step 8 or** study termination  (≥ 17 weeks) | ARM A (n = 40) | ARM B (n = 40) | |
| --- | --- | --- | --- |
|  |  |  |  |
|  | POTENT ART + IL-2 | POTENT ART ALONE | |
|  | Is CD4+ T-cell count ≥ 500 cells/mm3? | | |
|  |  |  |  |
|  |  |  |  |
|  |  |  |  |
| STEP 2  When CD4+ T-cell count is < 350 cells/mm3 x 2 consecutive visits, go to Step 3.  **If potent ART is resumed prematurely, go to Step 8 or study termination**  (Mean projected duration is 64 weeks with an estimated range of 32-96 weeks) | TREATMENT  INTERRUPTION | TREATMENT INTERRUPTION | |
|  |  |  |  |
|  |  |  |  |
| STEP 3  Yes: Continue to Step 4  No: Go to Step 8 or study termination  (24 weeks) | POTENT ART ALONE | POTENT ART ALONE | |
|  | At week +24, is plasma HIV-1 RNA < 200 copies/mL? | | |
|  |  |  |  |
|  |  |  |  |
|  |  |  |  |
| STEP 4  Yes: Continue to Step 5  No: Go to **Step 8 or** study termination  (≥ 17 weeks) | POTENT ART + IL-2 | POTENT ART ALONE | |
|  | Is CD4+ T-cell count ≥ 500 cells/mm3? | | |
|  |  |  |  |
| STEP 5  When CD4+ T-cell count is < 350 cells/mm3 x 2 consecutive visits, go to Step 6  **If potent ART is resumed prematurely, go to Step 8 or study termination**  (Mean projected duration is 64 weeks with an estimated range of 32-96 weeks) | TREATMENT  INTERRUPTION | TREATMENT  INTERRUPTION | |
|  |  |  |  |
|  |  |  |  |
| STEP 6  Yes: Continue to Step 7  No: Go to **Step 8 or** study termination  (24 weeks) | POTENT ART ALONE | POTENT ART ALONE | |
|  | At +24 weeks, is plasma HIV-1 RNA < 200 copies/mL? | | |
|  |  |  |  |
|  |  |  |  |
|  |  |  |  |
| STEP 7  (≥ 17 weeks) | POTENT ART + IL-2 | | POTENT ART ALONE |
| **STUDY ENDS** | | | |
| **STEP 8**  **(until study is closed)** | **Observation for subjects who fail to meet step inclusion criteria or follow study schema** | | |

# SUBSTUDY SCHEMA

A5109s: The Effect of Viral Replication on Antigen-Specific Proliferation Versus Cytokine Production in Subjects Undergoing Pulse Therapy: A Substudy of A5102

CHAIR John L. Schmitz, Ph.D.

DESIGN A5109s is a limited-center substudy designed to ascertain whether viral replication impairs lymphocyte proliferation in vivo in a subset of A5102 subjects.

SAMPLE SIZE

and POPULATION 16 subjects, approximately 8 from each arm of A5102.

Male and female HIV-1 infected subjects who are also enrolled in A5102.

DURATION This study will last 8 weeks, starting and ending during Step 2 (treatment interruption) of A5102. Subjects will register to the substudy immediately after registering to Step 2 of the main study.

REGIMEN Not applicable. Subjects will have additional immunology laboratory testing only.

## 1.0 STUDY OBJECTIVES

### 1.1 Primary Objective

1.1.1 To assess the CD4+ T-cell count at the end of Step 1 (IL-2 versus no IL-2 Step) and the slope of the decline in CD4+ T-cell count during Step 2 (treatment interruption).

### 1.2 Secondary Objectives

1.2.1 To analyze the safety/tolerability of the therapeutic approach utilized in this study.

1.2.2 To evaluate the duration of the first and second treatment interruption (length of Steps 2 and 5 – treatment interruption) for the entire study cohort.

1.2.3 To evaluate the rate of re-suppression of plasma HIV-1 RNA level to below the limit of detection (< 200 copies/mL) after potent ART is restarted following treatment interruption (Steps 3/4 and Steps 6/7).

1.2.4 To compare the duration of the first treatment interruption (Step 2) following the use of IL-2 versus no IL-2.

1.2.5 To evaluate the level of immune reconstitution achieved in Steps 3 and 4 and in Steps 6 and 7.

1.2.6 To analyze predictors of CD4+ decline in Arms A and B during Step 2 (treatment interruption).

1.2.7 To analyze the slope and magnitude of the increase in plasma HIV-1 RNA levels in Arms A and B during Step 2 (treatment interruption).

1.2.8 To compare the slopes of the changes in CD4+ T-cell counts and plasma HIV-1 RNA levels between subsequent treatment induction and interruption steps (Steps 3 and 4 versus Steps 6 and 7 and Step 2 versus Step 5 respectively) for subjects who continue to Steps 4 -7.

1.2.9 To compare lipid metabolism during potent ART periods (Steps 1, 3, 4, 6, and 7) versus the treatment interruption periods (Steps 2 and 5).

1.2.10 To compare glucose and insulin metabolism during potent ART periods (Steps 1, 3, 4, 6, and 7) versus the treatment interruption periods (Steps 2 and 5).

1.2.11 To compare the proportion of time on potent ART by month for Arm A versus Arm B.

1.2.12 To analyze the effects of treatment interruption on the proportion of naive and memory CD4+ cells.

1.2.13 To characterize replicative capacity, fitness, and evidence of antiretroviral drug resistance of virus isolates obtained from subjects when they have discontinued antiretroviral drugs.

**1.2.14 To determine if viral fitness near the initiation of treatment interruption correlates with CD4+ trajectory over the course of Step 2.**

**1.2.15 To determine if viral fitness shifts from the beginning to the end of Step 2.**

**1.2.16 To determine if there is evolution of viral fitness across interruption steps (e.g., the beginning of Step 2 versus the beginning of Step 4).**

**1.2.17 To assess the influence of immune activation on CD4+ T cell decline.**

### 1.3 A5109s Substudy Primary Objective

1.3.1 To ascertain whether viral replication impairs lymphocyte proliferation in vivo.

### 1.4 A5109s Substudy Secondary Objectives

- - 1. To ascertain whether antigen-specific cells are able to produce interferon‑γ (IFN‑γ).
    2. To ascertain whether plasma levels of proinflammatory cytokines correlate with the magnitude of lymphocyte proliferative impairment.
    3. To evaluate the change in the proportion of cells in S phase of cell cycle from the substudy entry evaluation to week 8 of Step 2.

## 2.0 INTRODUCTION

### 2.1 Background

Since the introduction of potent ART, we have seen both a dramatic decrease in the morbidity and mortality of subjects with advanced HIV(1-2) and a reconstitution of their immune systems. Infections that were previously impossible or difficult to treat, such as cryptosporidiosis, azole-resistant thrush, cytomegalovirus retinitis, *Mycobacterium avium* intracellular infections, and progressive multifocal leucoencephalopathy, have changed their naturally relentless courses. Multiple studies have demonstrated that if subjects experience a significant increase in CD4+ cells as a result of potent ART, the incidence of opportunistic infections (Ois) is extremely low, even in the absence of systemic prophylaxis (3-10). This finding has been true even in subjects with advanced disease and low CD4+ cell counts. These results have led to the current recommendation of stopping primary and secondary prophylaxis for most of the Ois in subjects with evidence of immune reconstitution. Therefore, the single best indicator of the capacity of the immune system to prevent the development of Ois is the number or percentage of CD4+ cells.

Despite these clinical improvements, there are also problems due to ART. First, in contrast to results observed in clinical trials, more than 50% of patients in large urban clinics who begin potent ART regimens also experience virologic failure over the first 2 years of treatment (11). Second, all components of potent ART regimens have major acute and long-term toxicities that are only partially understood (12-20). Among these toxicities are metabolic problems including diabetes mellitus, hyperlipidemia, lipodystrophy, lipoatrophy, and dyslipidemia. The mechanism(s) by which ART might cause these abnormalities is unknown. Finally, antiviral agents are difficult to take, require tight schedules and dietary restrictions, and are expensive. Nevertheless, current guidelines (21,22) suggest a very aggressive management of HIV-infected individuals, although the long-term clinical and virologic implications of this approach are unknown.

A more recent approach to reconstituting the diminished immune system caused by HIV is the use of potent ART in conjunction with IL-2. IL-2 is a cytokine secreted by activated T lymphocytes that regulates the proliferation and differentiation of lymphocytes, including CD4+ and CD8+ T cells. Althoughtreatment with IL-2 cancause transient increases inHIV-1 RNA levels,clinical studies with IL-2have revealed no long-termadverse effect upon theviral load. Indeed, when administered inthe setting of maximalviral suppression induced by potent ART, IL-2 therapy might help purgethe host’s latent viralreservoir through activation ofresting lymphocytes harboring provirus (23).Several studies have shown that the administration of intermittent IL-2 therapy resulted in sustained increases in the number of CD4+ cells, primarily in subjects with baseline CD4+ cell counts greater than 200 cells/mm3. Theseeffects generally exceed thoseachievable with even themost potent three-drug combinationsusing protease inhibitors insubjects with < 500 CD4+cells/mm3.

In one study by Kovacs et al. (24), 25 HIV-infected subjects were treated with IL-2 administered as a continuous infusion at a dosage of 6 to 18 million IU/day (MIU/day) for 5 days every 8 weeks during a period of 7 to 25 months. All subjects also received at least one approved antiviral agent. In 6 of the 10 subjects who had baseline CD4+ cell counts higher than 200 cells/mm3, IL-2 therapy was associated with a decline in the percentage of CD8+ lymphocytes expressing HLA-DR, an increase in the percentage of CD4+ lymphocytes that were positive for the p55 chain of the IL-2 receptor, and at least a 50% increase in the number of CD4+ cells. CD4+ cell count changes ranged from -81 to +2211 cells/mm3. The four subjects with baseline CD4+ cell counts > 200 cells/mm3 had transient but consistent increases in the plasma HIV-1 RNA levels at the end of each infusion. IL-2 therapy was associated with increased viral activation, few immunologic improvements, and substantial toxic effects in the 15 subjects who had CD4+ cell counts < 200 cells/mm3 (24).

In another study by Kovacs et al. (25), 60 HIV-infected subjects with baseline CD4+ cell counts > 200 cells/mm3 were randomly assigned to receive either IL-2 plus ART or ART alone. IL-2 was administered every 2 months for six cycles of 5 days each, starting at a dosage of 18 MIU/day. In those treated with IL-2, the mean CD4+ cell count increased from 428 +/- 25 cells/mm3 at baseline to 916 +/- 128 cells /mm3 at month 12, whereas, in the control group, the mean CD4+ cell count decreased from 406 +/- 29 cells/mm3 to 349 +/- 41 cells/mm3 (P < 0.001). There were no significant differences between the groups in terms of serial measurements of plasma HIV-1 RNA or p24 antigen concentration. Constitutional symptoms (fever, malaise, and fatigue) and asymptomatic hyperbilirubinemia were the chief dose-limiting toxic effects of IL-2 therapy (25).

Modest changes have also been seen with subcutaneously administered polyethylene glycosylated-rhIL-2 (PEG-rhIL-2) given on an intermittent schedule every 8 weeks (26). With a median subcutaneous (SQ) PEG-rhIL-2 dose of 8.5 MIU/day for 1-3 days every 8 weeks, after six cycles, a mean CD4+ cell increase of 18% was noted. Major dose-limiting toxicities seen with this outpatient regimen included flu-like symptoms, fluid retention, nausea, and diarrhea. In this study, subjects were also randomized to rhIL-2 by continuous intravenous infusion (CIV) or to antiviral therapy alone. Subjects receiving CIV rhIL-2 at a median dose of 12 MIU/day for 5 days every 8 weeks had a median CD4+ increase of 53% after six cycles, while the subjects on only antiretrovirals had a median CD4+ cell decrease of 16%. No significant changes in viral load were seen in any of these groups (26). In another study, subjects with HIV infection and CD4+ cell count > 250 cells/mm3 received rhIL-2 at a dose of either 2 or 4 MIU/m2 BID for 5 days (27). CD4+ cell counts increased an average 1.4-fold with this regimen with good tolerance and minimal toxicities consisting of fatigue, myalgias, nausea, diarrhea, and fever.

In another study, 49 HIV-positive subjects who were onstable ART and had baseline CD4+ cell counts≥ 500/mm3 were randomized to receive 5-day cycles of either low-dose(1.5 MIUBID) orhigh-dose (7.5 MIU BID) SQ IL-2 every 4or 8 weeks. High-dose recipients experienced meanslopes of +116.1 cells/monthand +2.7%/month in CD4+cells and percents, respectively,whereas low-dose recipients displayedmean slopes of +26.7and +1.3% in thesame parameters. At month6, high-dose recipients achieveda 94.8% increase inmean CD4+ cells overbaseline compared with a19.0% increase in low-doserecipients. While high-dose recipientsencountered more constitutional sideeffects, these were generallynot dose limiting. High-dose SQ IL-2 therapy in outpatients withearly HIV-1 infection waswell tolerated and induceddramatic, sustained rises inCD4+ cells (28).

Recently, Davey et al. reported on a trial comparing CD4+ T-cell responses and HIV-1 RNA levels among subjects receiving ART +/- IL-2 (29). At 1 year, the group receiving IL-2 achieved a mean increase in CD4+ T-cell count of 112% versus 18% in the control group. In the IL-2 group, 54% of the subjects experienced serious side effects at the starting dose of 7.5 MIU SQ BID.

Several presentations from the XIII International AIDS Conference in Durban, South Africa, reported on early results utilizing the strategic treatment interruption approach (30). Results from those small pilot trials suggest that HIV replication can be highly suppressed over consecutive courses following short treatment interruptions and that CD4+ T-cell counts can be maintained with perhaps some positive effect on HIV-specific immunity.

In the absence of treatment, HIV replication does not generally lead to development of new mutations linked to drug resistance. To address that concern, A5102 will look at the genotypes of viral isolates obtained at the end of drug interruptions and then among those subjects who fail to achieve high-level viral suppression upon restarting therapy. There is no viral threshold for restarting therapy, since it is expected that an effective potent ART regimen will be available. Therapy had previously been effective and new agents also will be available. Response to effective potent ART with regimens including protease inhibitors or efavirenz is good even if the viral level is > 100,000 copies/mL. Data presented in Durban (31) suggest that durable response to potent ART is more a function of CD4+ cell count than viral load and that response is likely to be good if the CD4+ T-cell count is > 200 cells/mm3. The CD4+ T-cell count for subjects restarting therapy in A5102 will be near 350 cells/mm3.

Since the primary endpoint for this study is the slope of the CD4+ T-cell count, restarting potent ART on the basis of the HIV-1 RNA level would confound our ability to assess the primary endpoint. We will have interim safety reviews of the study as well as a primary analysis planned at **6** months after all subjects have entered Step 2 (treatment interruption) so that if subjects are faring poorly from a clinical, CD4+ cell count, or virologic perspective, there will be an opportunity to revise or stop the study. Concern has been raised, for those subjects taking a nonnucleoside reverse transcriptase inhibitor (NNRTI) containing potent ART regimen, about the possibility of an NNRTI resistance emerging once therapy is stopped due to long plasma half-life and potential for rapid mutations. Subjects taking an NNRTI as part of their potent ART regimens during Step 1 or 3/4 will be instructed to stop their NNRTI 24-48 hours before beginning their treatment interruption.

### 2.2 Rationale

The current paradigm in treating HIV infection involves the early use of complex and costly combinations of antiretroviral medications (20,21). Although use of ART has resulted in significant clinical benefit, there is concern that the benefit may be waning and that alternative long-term therapeutic strategies need to be considered (32,33). **The most recent antiretroviral treatment recommendations issued in the spring/summer of 2002 have become more conservative with a greater focus on the CD4+ T-cell count. A threshold of < 350 cells/mm3 is now identified as the level below which antiretroviral therapy should be started in the asymptomatic patient which is consistent with the treatment strategy utilized in A5102 (34,35).**

A possible alternative approach to the management of HIV infection would be to administer antiviral therapy to HIV infected individuals only intermittently, when the CD4+ cell count drops below a critical threshold. This plan is based on recent pilot studies, which have demonstrated that, in some individuals, repeated interruptions of potent ART can result in a diminution of virologic rebound with subsequent treatment interruptions (**36,37**). The preliminary observations suggest that there may be some enhancement of the recovery of HIV-specific immunity, which is felt to be essential for the long-term control of HIV infection in the absence of potent ART (**38**).

Using this approach, subjects would continue to take potent ART for a specific period of time with the goal of completely suppressing viral replication and increasing the CD4+ cell count to a target value. ART would be interrupted again to repeat the cycle. The overall goal is to maintain the CD4+ cell count above the level that would put the individual at risk of Ois while minimizing the use of therapy. The rate of decay of CD4+ cell counts while off therapy varies individually; therefore, each subject will require different periods of episodic treatment. This kind of approach would theoretically decrease the incidence of long-term toxicities as well as virologic resistance.

In this pilot study we propose to evaluate intermittent potent ART, started and interrupted based exclusively on CD4+ cell counts, with or without IL-2, as an alternative approach to the chronic treatment of HIV infection. IL-2 will be given at 4.5 MIU SQ BID for 5 days for three cycles during Steps 1, 4, and 7. According to a literature review, this dose of IL-2 has been well tolerated and resulted in a significant increase in levels of CD4+ T-cells.

The study team hypothesizes that IL-2 given with potent ART will result in a longer off-therapy period for the subject than potent ART alone. It is further presumed that this strategy will not lead to an increase in the frequency of drug resistance and will reduce the toxicity associated with ART since it will decrease the amount of exposure to the drugs.

The A5102 team carefully considered numerous study designs to address the objectives of the study. Consideration was given to having a continued potent ART control arm. Since this study is a pilot study examining how long subjects could be safely off therapy utilizing a CD4+ T-cell count therapy threshold, it was decided that a potent ART control arm would slow accrual and contribute little to achieving the goals of the primary objective (CD4+ T-cell count after Step 1, potent ART +/- IL-2 and the slope of the CD4 + T-cell decline in the two study arms during the treatment interruption in Step 2). Given the increasing complexity of drug-related adverse events and the generally poor outcome in salvage studies, obtaining information about the safety and metabolic effects of a prolonged treatment interruption will provide important data to the AACTG to plan future studies possibly utilizing similar strategies.

The IL-2 arm is included to look at the CD4+ T-cell benefit at the end of Steps 1, 4, and 7 and the durability of any CD4+ T-cell increases by analyzing the CD4+ T-cell slope during the treatment interruptions (Steps 2 and 5). Data from either or both study arms can be used to design future studies. The time of the primary analysis (**6** months after the last subject is enrolled in Step 2) will allow for timely access to the initial key data for the purposes of planning future AACTG studies. The two-part design (primary analysis followed by a decision to continue the study cohorts as planned after review by both the study team and an independent safety review committee) allows the team to obtain early key information while preserving the opportunity to examine long-term outcomes.

**Immunology Rationale**

The goal of the immunologic studies in A5102 is to identify markers that predict the rate of decline in CD4+ T-lymphocyte number after cessation of ART. Host immunologic and genetic factors influence the rate of viral replication and disease progression in HIV-infected individuals. HIV-specific immune responses (CD4+ and CD8+) are felt important in the control of viral replication (**39,40**). As such, the presence of these HIV-specific responses might be expected to influence the rate of viral rebound and CD4+ T-lymphocyte decline in subjects after withdrawal of ART. To address this issue, we propose to collect and viably cryopreserve peripheral blood mononuclear cells (PBMCs) from all subjects enrolled in A5102. In future studies, to be proposed pending the findings of this study, HIV-specific immune responses will be evaluated in a case-control fashion comparing subjects with rapid versus slow CD4+ T-lymphocyte declines. The hypothesis to be tested is that higher levels of HIV responsiveness will be associated with slower CD4+ T-lymphocyte decline after cessation of ART. The specific assays may include one or more of the following: lymphocyte proliferation assay (LPA), cytotoxic T lymphocyte (CTL) assay, intracellular cytokine staining, ELISPOT, or major histocompatibility complex (MHC) tetramer assays. The assays to be performed will be determined at the time of proposal submission.

HIV infection results in heightened immune activation as evidenced by increases in cellular markers, such as CD38 antigen expression on CD8 T-lymphocytes, and levels of various cytokines and soluble markers. Several of these markers, including CD38 expression on CD8+ cells (**41**), β2 microglobulin, neopterin, and sTNFRII (33,**38**), have been shown to predict progression to AIDS. Recently, it has been demonstrated in a small number of subjects that increases in markers of immune activation occurred prior to increases in viral loads and the inflection point of CD4+ T-cell decline in subjects who progressed to AIDS (**42,43**). Soluble TNFRII levels were the earliest indicator of increased viral load, decline in CD4+ T cells and disease progression. A5102 will assess the ability of soluble and cellular markers of immune activation to predict the rate of decline in CD4+ T-lymphocyte counts after cessation of therapy. We propose to determine the expression of CD38 on CD8 T-lymphocytes in all subjects enrolled in this study using the core immunophenotyping panel. Additionally, plasma will be obtained and frozen for batch analysis of sTNFRII levels in subjects.

**A5102 provides a cohort of subjects in which proposed mechanisms of immune activation driven CD4+ T cell decline can be assessed. Evidence is mounting that the mechanism underlying the association of immune activation and disease progression (CD4+ T-cell death) is activation driven T-cell turnover and cell death (44,45). Immune activation, as discussed above, is a hallmark of HIV infection. More recently, several studies investigating T-cell dynamics in HIV-1 infected patients have demonstrated increased levels of lymphocyte turnover by in vivo labeling studies (46). Not only was HIV infection associated with an increased proportion of labeled cells, but also a rapid decline in label upon cessation of administration of labeling agent suggesting an increased rate of cell death. In studies by Kovacs (47) it was shown that in vivo labeling was lower in subjects receiving HAART but that the decay rates of cells were similar in treated and untreated patients suggesting that cell death was independent of HIV.**

**Several other studies support the contention that immune activation driven cell turnover is largely responsible for CD4+ T cell decline. SIV infection of sooty mangabeys and African green monkeys does not result in progressive depletion of CD4+ T cells despite high levels of viral replication. Interestingly, these animals do not show significant immune activation or cell turnover (48,49). When untreated HIV-1 infected patients were compared to patients failing a HAART regimen who continue to have increasing CD4+ T cell counts (discordant responders), CD4+ turnover was higher, regardless of the level of viremia, in the untreated patients (50). For the same level of CD4+ depletion, HIV-1 and HIV-2 infected patients have similar levels of activated and cycling T cells despite large differences in viremia (51). Hazenberg, et al. demonstrated that increasing proportions of dividing CD4+ and CD8+ T cells as well as activation markers on CD4+ T cells were independent predictors of progression to AIDS, including loss of naïve CD4+ and CD8+ T cells in 102 subjects from the Amsterdam Cohort Studies on HIV-1 infection and AIDS (52).**

**We hypothesize that the level of immune activation at registration to Step 2 will correlate with the rate of CD4+ T-cell decline in subjects stopping antiretroviral therapy. Increased rates of CD4+ cell death will also be reflected in increased T-cell turnover (Ki-67).**

**We propose to determine baseline (registration to Step 2) and off therapy (Step 2 week 24 and at the time CD4+count <350 cells/mm3 on two consecutive visits) levels of immune activation, including both cellular as well as a soluble markers of activation. We will utilize cryopreserved PBMCs to assess CD38/CD70 expression on CD4+ and CD8+ T cells to assess the ability of activated T cells to predict CD4+ decline (52-54). It has been shown that uncontrolled HIV replication is associated with upregulation of adhesion molecule expression (55). We will assess soluble ICAM-1 levels to ascertain whether the magnitude of adhesion molecule upregulation is associated with CD4+ decline and whether this marker adds information regarding prediction of CD4+ decline to cell-associated markers of immune activation. CD4+ and CD8+ proliferation, hypothesized to be driven by immune activation will be assessed by Ki-67 labeling utilizing cryopreserved PBMC’s (56,57) from the same time points as samples for immune activation studies.**

IL-2 therapy is associated with increases in absolute CD4+ T-lymphocyte counts. T-lymphocyte subset analysis has demonstrated that by 12 to 24 weeks after administration of IL-2, naïve CD4+ T-lymphocytes (CD45RA+) show significant increases (**58,59**). While this is felt to bode well in terms of immune reconstitution, it has been demonstrated in ACTG 315 that subjects experiencing higher increases in naïve CD4+ T-lymphocytes were more likely to experience loss of viral suppression while on therapy. It was hypothesized that the increased naïve T-lymphocytes in these subjects provided more targets for viral infection and replication and thus increased viral loads. If this is true, one might expect that subjects with greater increases in naïve CD4+ T-lymphocyte counts would also experience a more rapid decline in CD4+ T-lymphocyte numbers after cessation of ART. We will address this question by determining the association between the number of naïve (CD45RA+/CD62L+) CD4+ T-lymphocytes in subjects at baseline and/or at the time of cessation of therapy and the rate of decline in CD4+ T-lymphocyte counts after cessation of therapy. Phenotypic analysis will be carried out with the core immunophenotyping panel at baseline, at the time of cessation of ART, and periodically after withdrawal of therapy.

Host genetic factors have also been identified that appear to influence the rate of disease progression in HIV-infected individuals. Among the most studied of the genetic factors are the CCR5 chemokine receptor deletion (**60**) and promoter polymorphisms (**61**). Chemokine receptor polymorphisms have been determined to affect the rate of decline in CD4+ T-lymphocyte counts (**62,63**). It has been hypothesized that genetic polymorphisms may affect the expression of the chemokine receptor CCR5 on CD4+ T-lymphocytes (**61,64**). We hypothesize that the level of expression of CCR5 might be used to predict the rate of decline in CD4+ T-lymphocyte number. We will assess the percentage of CCR5 expression CD4+ T-lymphocytes and the quantitative expression of the molecule on the surface of these cells at the time of discontinuation of ART to determine the association between these variables and CD4+ cell decline.

**Virology Rationale**

**Proviral DNA in circulating PBMCs is believed to archive integrated provirus, resulting from infection of lymphoid tissues during acute and early HIV-1 infection (23). HIV-1 proviral DNA persists in PBMCs (thought to be largely memory CD4+ cells) despite the prolonged administration of HAART (65-67) and suppression of HIV-1 RNA replication (65-67). Attempts to calculate the total body burden of provirally infected cells, their half-life and trafficking, and the evolution of viral resistance has been studied in the context of patients receiving prolonged antiretroviral therapy (66-69) and in a small number of patients receiving IL-2 (70).**

**Methods to quantify proviral DNA include the use of a research only quantitative PCR assay (71) as well as a number of Taq-man amplification systems (72,73). HIV proviral DNA expressed as copies per million cells prior to antiretroviral therapy is correlated with HIV-1 RNA and inversely related to CD4+ cell number (65,74-76). In a recent retrospective study from the ACTG 175 team, Tierney demonstrated that baseline, proviral DNA copies were independently associated with an increased risk of death and disease progression (77) using the Roche research only assay. We hypothesize that, in the context of A5102, proviral quantification in chronically infected suppressed individuals at baseline, as a measure of latent infection may predict the rate and magnitude of return of HIV viremia after treatment interruption as well as the rate of CD4+ cell decline. The randomized comparison of IL-2 treatment in ACTG A5102 further affords the opportunity to identify whether there is a decrease in proviral DNA levels, associated with IL-2 treatment (versus control) and if there are associations between week 0 (randomization) and week 16 (end of Step 1) changes in proviral DNA associated with PI versus NNRTI treatments or the administration of IL-2 during virologic suppression.**

**Three whole virus competitive fitness assays are proposed for A5102. The first will compare 10 first passage viral isolates at Step 2, week 2 from A5102 subjects with favorable Step 2 CD4+ cell trajectories to 10 from subjects with rapidly declining Step 2 CD4+ cell trajectories to determine if the two subgroups have different fitness indices. (Step 2 CD4+ cell trajectories will be obtained by the protocol statistician.) Fitness, along with other viral and immunologic determinants, may be one factor which affects CD4+ cell count stability in a structured treatment interruption (STI). A whole virus assay that can rank inter-isolate fitness is desirable in this treatment interruption setting because fitness may be primarily determined by non-RT/protease viral characteristics in the absence of selective drug pressure. Week 2 was chosen because these isolates are already collected and stored per the virology section of the A5102 protocol. Hence this project could be done fairly soon.**

**The second whole virus competitive fitness assay will compare Step 2, week 2 fitness versus Step 3 entry fitness (baseline treatment resumption, last time point of Step 2) on isolates from 10 to 40 of the A5102 subjects to see if fitness indices evolve over time off therapy (on STI) and if fitness may be a useful future predictor (in other studies) to include in “when to re-start” determinations. Although there is no selective drug pressure in Step 2, viral fitness may still change over the course of the treatment interruption as archived virus repopulates and as non-pharmacologic pressures shape the individual’s virus pool. A whole virus fitness assay which accounts for all viral determinants of fitness may be able to demonstrate such evolution. In addition, fitness is not currently considered in determining when to resume therapy in CD4+ cell count driven STI protocols. It has been proposed to be a possible independent predictor of disease course, however, and therefore needs a pilot evaluation of whether it might be a useful parameter to consider when deciding on treatment resumption. This project will require subjects to reach Step 3 and will also require growing isolates from PBMCs collected at the later time point. Hence project 2 will have a longer time-frame.**

**The third assay will compare Step 2, week 2 fitness versus Step 5, entry fitness on isolates from 10 to 40 A5102 subjects to determine if fitness evolves from the first to the second STI. Comparison of various viral parameters including fitness is needed over serial STIs, to determine if viral characteristics evolve favorably or unfavorably. Since A5102 subjects will have to enter Step 5, this project is quite far in the future.**

**Metabolic Rationale**

**A significant proportion of HIV positive individuals on antiretroviral therapy can experience a myriad of metabolic complications, including but not limited to peripheral lipoatrophy, gain in visceral fat, hyperlipidemia, and insulin resistance. The relative contribution of therapy and HIV-1 infection itself is still under investigation. Increasingly, concerns about metabolic and other adverse effects related to long-term antiretroviral therapy influence the approach to treatment of HIV-1 infection. The recently revised Department of Health and Human Services treatment guidelines reflect that concern. ACTG 5102 provides a unique opportunity to evaluate the effects of the discontinuation of antiretroviral therapy in HIV-1 infected individuals with well-controlled viral replication. If antiretroviral therapy is indeed the main culprit of the metabolic complications associated with treatment, then prolonged discontinuation of antiretroviral therapy would be expected to lead to reversal or an improvement of metabolic abnormalities, unless theses changes are completely irreversible. Because HIV replication will be unabated, the effects of HIV-1 infection itself will be maintained. The lead-in period of ACTG 5102 also gives us the opportunity to investigate the effects of IL-2 on metabolic parameters. There are no data in the literature that evaluate the effects of IL-2 in lipid parameters. This information is critical if IL-2 is going to be used as an immunomodulator in HIV-1 infected patients.**

**In ACTG 5102 we will study the direct effects of stopping antiretroviral therapy on lipid and glucose metabolism (glucose, insulin, LDL cholesterol, triglycerides and HDL cholesterol).**

**We also propose to study the effects of treatment discontinuation on C-reactive protein levels. C-reactive protein is a marker of inflammationthat has been shown in several prospective, nested case–controlstudies to be associated with an increased risk of myocardialinfarction, stroke, sudden death from cardiaccauses,and peripheral arterial disease. In a recent publication in the New England Journal of Medicine, C-reactive protein levelwas a stronger predictor of cardiovascular events than the LDLcholesterol level and it was felt that C-reactive protein adds prognostic information tothat conveyed by the Framingham risk score. The results of ACTG 372 suggest that patients with fully controlled viral replication receiving indinavir containing antiretroviral therapy have high levels of C-reactive protein. In fact initiation of antiretroviral therapy was associated with a dramatic increase in C-reactive protein levels in that study (K. Henry, unpublished observation). Recently, antiretroviral therapy has been associated in large cohort studies with a slight increase in cardiovascular risk. Our hypothesis is that discontinuation of antiretroviral therapy will lead to a decrease in C-reactive protein and an overall decrease in the cardiovascular risk of these individuals.**

**We would like to measure the above mentioned marker at 5 time points during A5102.**

- **At entry and at week 16 during Step 1 (subjects on antiretroviral therapy with or without IL-2). These measurements will allow us to evaluate the impact of IL-2, an area where no data are available at the present time.**
- **At week 4, 8, and 24 after the discontinuation of ARV therapy. Using 3 time points will help characterize the kinetics of the change of the metabolic marker after discontinuation of antiretroviral therapy, will increase the power of the study (because the use of longitudinal analysis and repeated measures), and will help to clarify the role of HIV itself in the development of these abnormalities. Metabolic changes observed during the first 4 weeks after discontinuation (when the duration of viral relapse is fairly minimal) could be attributed to the pharmacologic effects of the drug treatment. Changes still present after 6 months of discontinuing the antiretroviral regimen, when the new “HIV viral set point” off antiretroviral therapy has been reached again, would more likely be related to HIV-1 infection itself.**

**In spite of the lack of good prospective trials, the use of “CD4+ driven” therapy has become a popular strategy among clinicians. Understanding better the metabolic implications of this strategy is a clear priority that fits perfectly in the ACTG agenda. A5102 provides a unique and timely opportunity to investigate the effects of discontinuing antiretroviral therapy and the role of drugs and HIV itself in the development of the metabolic problems associated with HIV-1 infection. The metabolic studies of A5102 will be a significant and important contribution to the literature.**

### 2.3 Substudy Background and Rationale

HIV-1 infection causes a progressive loss and dysfunction of circulating CD4+ T-lymphocytes. In vitro, HIV products such as Tat or gp-120 can induce apoptosis and lymphocyte dysfunction (**78,79**). Many mechanisms have been postulated for the induction of lymphocyte dysfunction; these include sensitization of T-cells to apoptosis (**78-80**), entry into cell cycle and inhibition of IL-2 production (**81**), and interference with antigen-presenting cell function (**82**). Some of these effects may be due to potentiation of proinflammatory cytokines such as tumor necrosis factor (TNF-α).

Recently it has been shown that HIV-1 replication causes deterioration in lymphocyte proliferative responses to HIV and non-HIV antigens even without significant CD4+ cell depletion (**83**). Despite this inability to proliferate, antigen-specific cells were present and were able to produce interferon-γ (IFN-γ). Most subjects are expected to develop lymphocyte proliferative responses to these antigens (**84**). Since subjects in A5102 will interrupt therapy with high circulating CD4+ lymphocyte counts, we will be able to ascertain whether antigen-specific lymphocyte proliferative responses are impaired by high-level viral replication and whether these cells are still able to produce IFN-γ. We hypothesize that HIV replication will cause an impairment of HIV and non HIV-specific lymphocyte proliferation. We also want to know whether these defects in proliferation are associated with increased expression of surface markers of activation and with lymphocyte entry into cell cycle. Furthermore, we hypothesize that proinflammatory cytokines may be intermediaries between viral replication and lymphocyte dysfunction. To test this hypothesis, we will ascertain whether the magnitude of impairment in lymphocyte proliferation is associated with plasma levels of proinflammatory cytokines.

## 3.0 STUDY DESIGN

A5102 is an open-label, pilot, multicenter, multistep study randomizing subjects to +/- IL-2. The study is designed to evaluate the intercept and slope of CD4+ T-cell counts during Step 2 in HIV-infected individuals who have been treated with stable potent ART for at least 3 months prior to enrollment and have CD4+ T-cell counts ≥ 500 cells/mm3 and plasma HIV-1 RNA < 400 copies/mL at screening and have plasma HIV-1 RNA < 200 copies/mL at preentry. Potent ART will be initiated and interrupted, based on CD4+ T-cell counts, as an alternative approach to the chronic management of HIV infection.

IL-2 will be administered as rhIL-2, 4.5 MIU SQ BID for 5 days every 8 weeks for three cycles during Steps 1, 4, and 7 for subjects who are on Arm A.

Subjects must provide their own potent ART for the duration of the study. Potent ART is currently defined as three or more antiretroviral agents or dual protease inhibitors. No restrictions will be placed on the potent antiretroviral drug combinations. However, it is strongly recommended that they involve FDA-approved medications used in accordance with the most recent Department of Health and Human Services (DHHS) treatment recommendations on potent ART as outlined in [**http://www.aidsinfo.nih.gov**](http://www.aidsinfo.nih.gov/).

This study has **eight** steps. After randomization to Step 1, subjects must register to each successive step **(Step 2 through Step 7)** within 6 weeks after completing the evaluations on the last visit of the previous step. **There is no time limitation for subjects to register to Step 8.** For Steps 2 and 5, treatment interruption is defined as the withdrawal of both potent ART and IL-2 therapy.

Step 1: Potent ART + Cycles of IL-2 Versus Potent ART Alone

Subjects will begin Step 1 if their CD4+ T-cell count is ≥ 500 cells/mm3 during screening, and if their plasma HIV-1 RNA is < 400 copies/mL during screening and < 200 copies/ mL at preentry. Subjects will be randomized to either potent ART and IL-2 therapy or potent ART alone. Subjects in Arm A will receive potent ART for approximately 17 weeks in addition to three cycles of IL-2 while subjects in Arm B will receive potent ART alone.

Step 2: Treatment Interruption (No IL-2 and No Potent ART)

Subjects will be eligible for Step 2 if their CD4+ T-cell count is ≥ 500 cells/mm3 at the latest of either 1) week 17 of Step 1 or 2) at the visit immediately following IL-2 cycle #3 (in the event of a delayed cycle) or 3) at the visit in which it is determined that IL-2 cycle #3 will be skipped. **Subject who f**ail to maintain a CD4+ T-cell count ≥ 500 cells/mm3 at the conclusion of Step 1 will **either register to Step 8 or be** taken off study. Upon registration to Step 2, subjects in Arm A will stop both potent ART and IL-2 therapy, and subjects in Arm B will stop potent ART. All subjects will have their CD4+ T-cell count measured every 4 weeks until step completion. **Subjects who resume potent ART while on Step 2 can either register to Step 8 or be taken off study.**

Step 3: Potent ART Alone

Subjects will be eligible to register to Step 3 when the CD4+ T-cell count is < 350 cells/mm3 on two successive determinations during Step 2. In the event of a missed visit, the two successive CD4+ draws cannot be > 8 weeks apart. Once subjects register to Step 3, only potent ART will be restarted. Subjects will remain on potent ART for 24 weeks.

Step 4: Potent ART + Cycles of IL-2 Versus Potent ART Alone

Subjects will be eligible to register to Step 4 if the HIV-1 RNA level is < 200 copies/mL at +24 weeks after starting Step 3. Only the RNA value at week +24 of Step 3 will be used to determine eligibility in Step 4, even if the subject has an HIV-1 RNA value < 200 copies/mL at an earlier time during Step 3. If the subject’s RNA level is > 200 copies/mL at week +24 of Step 3, the subject **can either register to Step 8 or** be taken off study. During Step 4, subjects will continue to receive potent ART with or without IL-2 for approximately another 17 weeks according to their original treatment assignment.

Step 5: Treatment Interruption (No IL-2 and No Potent ART)

Subjects will be eligible for Step 5 if their CD4+ T-cell count is ≥ 500 cells/mm3 at the latest of either 1) week +17 of Step 4 or 2) at the visit immediately following IL-2 cycle #6 (in the event of a delayed cycle) or 3) at the visit in which it is determined that IL-2 cycle #6 will be skipped. **If subjects fail** to achieve a CD4+ T-cell count ≥ 500 cells/mm3 at the conclusion of Step 4 **the subject can either be registered to Step 8 or be** taken off study. Upon registration to Step 5, subjects in Arm A will stop both potent ART and IL-2 therapy, and subjects in Arm B will stop potent ART. All subjects will have their CD4+ T-cell count measured every 4 weeks until step completion. **Subjects who resume potent ART while on Step 5 can either register to Step 8 or be taken off study.**

Step 6: Potent ART Alone

Subjects will be eligible to register to Step 6 when the CD4+ T-cell count is < 350 cells/mm3 on two successive determinations during Step 5. In the event of a missed visit, the two successive CD4+ draws cannot be > 8 weeks apart. Once subjects register to Step 6, only potent ART will be restarted. Subjects will remain on potent ART for 24 weeks.

Step 7: Potent ART + Cycles of IL-2 Versus Potent ART Alone

Subjects will be eligible to register to Step 7 if their HIV-1 RNA level is < 200 copies/mL at +24 weeks after starting Step 6. Only the RNA value at week +24 of Step 6 will be used to determine eligibility to Step 7, even if the subject has an RNA value < 200 copies/mL at an earlier time during Step 6. If the subject’s HIV-1 RNA level is > 200 copies/mL at week +24 of Step 6, the subject will be taken off study. During Step 7, subjects will continue to receive potent ART with or without IL-2 for approximately another 17 weeks according to their original treatment assignment. **Subjects who complete Step 7 will not continue on to Step 8 but rather will complete the study termination evaluations as outlined in section 6.1.**

**Step 8: Observation**

**Subjects will be eligible to register to Step 8 if they either have failed the eligibility criteria to the next sequential step, do not want to follow the study schema or they are advised by the primary care physician not to follow the study schema and the**y **agree to being followed.** **During Step 8, subjects will be required to have laboratory and clinical evaluations performed every 6 months until the study is closed. The study will close when the last subject has completed following the protocol schema (Step 1 through Step 7).**

## 4.0 SELECTION AND ENROLLMENT OF SUBJECTS

### 4.1 Inclusion Criteria – Protocol Entry/Step 1

4.1.1 HIV-1 infection, as documented by any licensed ELISA test kit and confirmed by Western blot at any time prior to study entry. A positive HIV-1 culture, HIV-1 antigen, plasma HIV-1 RNA, or a second antibody test by a method other than ELISA is acceptable as an alternative confirmatory test.

**4.1.2** Subjects must **not have changed their potent antiretroviral regimen during the three months** prior to entry.

NOTE: Potent ART is currently defined as three or more antiretroviral agents or dual protease inhibitors. No restrictions will be placed on the potent antiretroviral drug combinations. However, it is strongly recommended that they involve FDA-approved medications used in accordance with the most recent DHHS treatment recommendations on potent ART available on the Web at [**http://www.aidsinfo.nih.gov**](http://www.aidsinfo.nih.gov/)**.**

**4.1.3 Eligible subjects must be on a potent antiretroviral regimen with a viral load < 400 copies/mL for > 6 months prior to study entry with reasonable future treatment options remaining (as assessed by the site principle investigator).**

4.1.4 CD4+ T-cell count ≥ 500 cells/mm3 obtained within 90 days prior to study entry (screening visit) at any **laboratory that has a CLIA certification or its equivalent.**

NOTE: Subjects with a CD4+ T cell count < 500 cells/mm3 at preentry or entry are still eligible for study entry as long as the screening visit value is ≥ 500 cells/mm3. Subjects will be stratified based on nadir CD4+ T-cell counts.

4.1.5 HIV-1 RNA < 400 copies/mL within 90 days (screening visit) prior to study entry by the UltraSensitive Amplicor HIV-1 Monitor™ assay as documented by laboratory report from a **CLIA-**certified laboratory.

4.1.6 HIV-1 RNA < 200 copies/mL within 30 days (preentry) prior to study entry by the UltraSensitive Roche Amplicor HIV-1 Monitor™ assay as documented by a laboratory report from a **CLIA**-certified laboratory.

- - 1. Laboratory values obtained within 30 days prior to study entry:
- Absolute neutrophil count (ANC) ≥ 750/mm3.
- Hemoglobin ≥ 8.5 g/dL.
- Platelet count ≥ 50,000/mm3.
- Creatinine ≤ 1.5 x upper limit of normal (ULN).
- AST (SGOT), ALT (SGPT), and alkaline phosphatase ≤ 5 x ULN.
- Total bilirubin ≤ 2.5 x ULN unless elevation is due to elevated indirect bilirubin in a subject receiving indinavir and in the absence of other evidence of significant liver disease.
- Serum amylase ≤ 1.5 x ULN (if serum amylase is > 1.5 x ULN, pancreatic amylase or lipase must be ≤ 1.5 x ULN).

4.1.8 All women of reproductive potential (have not reached menopause nor undergone hysterectomy, oophorectomy, or tubal ligation) must have a negative serum or urine β-HCG pregnancy test performed within 30 days before initiating study-specified medication(s) and it must be repeated at day 0.

Female subjects who are not of reproductive potential (have reached menopause or undergone hysterectomy, oophorectomy, or tubal ligation) or whose male partner has undergone successful vasectomy with resultant azoospermia or has azoospermia for any other reason, are eligible without requiring the use of contraception. Documentation of menopause, sterilization (hysterectomy, oophorectomy, tubal ligation, or vasectomy) and azoospermia by patient-reported history is acceptable.

All subjects must not participate in a conception process (i.e. active attempt to become pregnant or to impregnate, sperm donation, in vitro fertilization), and if participating in sexual activity that could lead to pregnancy, the female study volunteer/male partner must use a form of contraception as specified in the note below while receiving protocol-specified medication(s) and for one month after stopping the medication(s).

NOTE: Some protocol-required medications interact with hormonal-based methods. Therefore, hormonal-based methods alone are not sufficient. At least one of the following methods MUST be used appropriately (with or without a hormonal-based method) unless documentation of menopause, sterilization, or azoospermia is present:

- Condoms (male or female) with or without a spermicidal agent
- Diaphragm or cervical cap with spermicide
- IUD

4.1.9 Karnofsky performance score ≥ 90 within 30 days prior to study entry. (Refer to the Protocol Management Handbook for a copy of the Karnofsky Performance Scale).

4.1.10 Men and women ≥ 18 years of age.

4.1.11 Ability and willingness of subject or legal guardian/representative to give written informed consent.

4.1.12 Willingness to participate in the study for up to 4 years or longer.

4.1.13 Willingness of subject’s primary care provider to have the subject in the study and to comply with DHHS guidelines for potent ART.

### 4.2 Exclusion Criteria

- - 1. Any active or past **significant** AIDS-**related** illness with the exception of minimal (less than 10 lesions) cutaneous Kaposi’s sarcoma**, pulmonary TB, or bacterial pneumonia.**

NOTE: Subjects with a history of a CD4+ T-cell count < 200 cells/mL but who have not had an AIDS-defining illness are still eligible.

- - 1. Use of any known immunomodulatory therapy within 4 weeks prior to study entry including but not limited to drugs such as systemic corticosteroids, interferons, interleukins, thalidomide, granulocyte-macrophage colony-stimulating factor, dinitrochlorobenzene, inosiplex; polyribonucleoside; ditiocarb, IV gammaglobulin, or human growth hormone.
    2. Use of hydroxyurea within 3 months prior to study entry.
    3. Any prior systemic treatment with rhIL-2.
    4. Treatment for active cardiac disease with any of the following medications: antianginal agents such as nitrates, calcium channel blockers, and beta-blockers, or antiarrhythmic agents such as digitalis and afterload reducers within 30 days prior to study entry.
    5. Significant cardiac insufficiency (New York Heart Association Class > 2). The classification guidelines can be found at [**http://www.americanheart.org/presenter.jhtml?identifier=1712**](http://www.americanheart.org/presenter.jhtml?identifier=1712)

NOTE: Subjects with isolated hypertension or elevated plasma lipids but no cardiac disease will be eligible for this study. In addition, subjects using calcium channel blockers or beta-blockers for isolated hypertension who do not have active cardiac disease are still eligible.

- - 1. Any malignancy requiring systemic or local toxic chemotherapy. Local radiation will be allowed.
    2. Pregnancy or breast-feeding.
    3. Thyroid disease that has not been controlled with medication for ≥ 3 months prior to study entry.

NOTE: Subjects must continue stable thyroid medication throughout their participation in this study.

- - 1. History of autoimmune disease, including inflammatory bowel disease, psoriasis, and optic neuritis.
    2. Uncontrolled diabetes mellitus (defined as fasting blood glucose > 126 mg/dL or random blood glucose levels > 200 mg/dL on at least two occasions within 6 months prior to study entry).
    3. Medical history of a major organ transplantation.
    4. Active alcohol or substance abuse or psychiatric illness, which in the opinion of the investigator will interfere with adherence to study requirements.
    5. Clinically significant neurological disorder either diagnosed or occurring within 1 year prior to study entry that in the opinion of the principal investigator would affect the subject’s study compliance or safety.
    6. Use of astemizole (Hismanal), midazolam (Versed), **or** triazolam (Halcion) within 2 weeks prior to study entry.
    7. Use of systemic corticosteroids for ≥ 4 weeks within 3 months prior to study entry.
    8. Clinically significant respiratory or gastrointestinal disease that, in the opinion of the principal investigator, might affect subject safety.

### 4.3 Inclusion Criteria – Steps 2 through **8**

- - 1. Step 2 (Treatment Interruption)

4.3.1.1 CD4+ T-cell count ≥ 500 cells/mm3 obtained at the latest of either 1) week 17 of Step 1 or 2) at the visit immediately following IL-2 cycle #3 (in the event of a delayed cycle) or 3) at the visit in which it is determined that IL-2 cycle #3 will be skipped.

4.3.1.2 Registration to Step 2 must occur within 6 weeks after the CD4+ T-cell sample is collected per section 4.3.1.1.

4.3.1.3 Negative pregnancy test.

4.3.2 Step 3 (Potent ART Alone)

4.3.2.1 CD4+ T-cell count < 350 cells/mm**3** on two consecutive visits during Step 2.

NOTE: In the event of a missed visit, the two successive CD4 draws cannot be > 8 weeks apart.

4.3.2.2 Registration to Step 3 must occur within 6 weeks after the second CD4+ T-cell count is drawn per section 4.3.2.1.

4.3.2.3 Negative pregnancy test.

4.3.3 Step 4 (Potent ART +/- IL-2)

4.3.3.1 Plasma HIV-1 RNA is < 200 copies/mL at the Step 3 week +24 visit as analyzed by the UltraSensitive Roche Amplicor assay.

4.3.3.2 Registration to Step 4 must occur within 6 weeks after the plasma HIV-1 RNA is collected for Step 3 week +24.

4.3.3.3 Negative pregnancy test.

4.3.4 Step 5 (Treatment interruption)

4.3.4.1 CD4+ T-cell count ≥ 500 cells/mm**3** obtained at the latest of either 1) week +17 of Step 4 or 2) at the visit immediately following IL-2 cycle #6 (in the event of a delayed cycle) or 3) at the visit in which it is determined that IL-2 cycle #6 will be skipped.

4.3.4.2 Registration to Step 5 must occur within 6 weeks after the CD4+ T-cell sample is collected per section 4.3.4.1.

4.3.4.3 Negative pregnancy test.

4.3.5 Step 6 (Potent ART Alone)

4.3.5.1 CD4+ T-cell count < 350 cells/mm**3** on two consecutive visits during Step 5.

NOTE: In the event of a missed visit, the two successive CD4+ draws cannot be > 8 weeks apart.

4.3.5.2 Registration to Step 6 must occur within 6 weeks after the second CD4+ T-cell count is drawn per section 4.3.5.1.

4.3.5.3 Negative pregnancy test.

4.3.6 Step 7 (Potent ART +/- IL-2)

4.3.6.1 Plasma HIV-1 RNA < 200 copies/mL at the Step 6 week +24 visit as analyzed by the UltraSensitive Roche Amplicor assay.

4.3.6.2 Registration to Step 7 must occur within 6 weeks after the plasma HIV-1 RNA is collected for Step 6 Week +24.

4.3.6.3 Negative pregnancy test.

- - 1. **Step 8 (Observation)**

**Subjects are eligible to register to Step 8 if at least one of the following conditions has been met:**

**4.3.7.1 Subject has failed the eligibility criteria to the next sequential step**

**OR**

**4.3.7.2 Subject does not want to follow the study schema**

**OR**

**4.3.7.3 Subject is advised by the primary care physician not to follow the study schema and the subject agrees.**

### 4.4 Inclusion Criteria – A5109s

- - 1. Subjects must have completed Step 1 of A5102.
    2. Subjects must register to A5109s immediately after registering to Step 2 of A5102.

### 4.5 Study Enrollment Procedures

4.5.1 Prior to implementation of this protocol, sites must have the protocol and consent form approved by their local institutional review board (IRB). Sites must be registered with and approved by the Regulatory **Compliance** Center Site Registration Desk. Site registration must occur before any subjects can be enrolled in this study.

Once a candidate for study entry has been identified, details will be carefully discussed with the subject. The subject will be asked to read and sign the consent form that was approved by both the ACTU’s IRB and the ACTG Site Registration Desk.

4.5.2 The Division of AIDS has concluded that this protocol does NOT meet Federal requirements governing prisoner participation in clinical trials and should NOT be considered by local IRBs for the recruitment of prisoners.

4.5.3 Enrollment for A5102 will be a multi step process. Initially, all subjects will be randomized at entry to Step 1 according to standard procedures with the Data Management Center. Study treatment must be initiated within 7 days of randomization to Step 1 unless indicated otherwise. Subjects who meet the eligibility criteria for each subsequent step, as outlined in section 4.3, will register in chronological order to steps 2 through 7 according to standard procedures with the Data Management Center. When a subject is registered to a subsequent step, a new SID number will be assigned. **Subjects who are eligible to enter Step 8 (see section 4.3.7) will register according to standard procedures with the Data Management Center.**

### 4.6 Substudy Enrollment Procedures

4.6.1 Enrollment will be limited to the following sites **and their subunits**: Case Western Reserve University, **Duke University Medical Center,**

**Northwestern University,** and University of North Carolina at Chapel Hill.

4.6.2 Subjects will register to the substudy immediately after registering to Step 2 of the main study.

### 4.7 Coenrollment Guidelines

Coenrollment into ACTG studies or non-ACTG studies will be considered on a case by case basis. For specific questions and approval regarding coenrollment, contact the protocol chair or vice chairs via e-mail.

## 5.0 TREATMENT

### 5.1 Regimens, Duration, and Administration

5.1.1 Regimens and Duration

| STEP | DURATION  (weeks) | ARM A | ARM B |
| --- | --- | --- | --- |
| Step 1 | ≥ 17 | Potent ART + IL-2 | Potent ART |
| Step 2 | 64** | Treatment Interruption | Treatment Interruption |
| Step 3 | 24 | Potent ART | Potent ART |
| Step 4 | ≥ 17 | Potent ART + IL-2 | Potent ART |
| Step 5 | 64** | Treatment Interruption | Treatment Interruption |
| Step 6 | 24 | Potent ART | Potent ART |
| Step 7 | ≥ 17 | Potent ART + IL-2 | Potent ART |
| **Step 8*** | **Until study**  **is closed** | **Observation** | **Observation** |

**Mean projected time is 64 weeks with an estimated range of 32-96 weeks

***For subjects who fail to meet step inclusion criteria or to follow the schema.**

After entry to Step 1, refer to the Study Design in section 3.0 for CD4+ T-cell counts or RNA levels required for registration to subsequent steps. However, s**ubjects who complete Step 7 will not continue on to Step 8 but rather will complete the study termination evaluations as outlined in section 6.1.** Antiretrovirals will not be provided through the study. For acceptable antiretroviral regimens and information on staggered stopping of regimens containing efavirenz or nevirapine, see section 5.4.1, Required Medications.

Only Arm A subjects will receive IL-2. Cycles of IL-2: 4.5 MIU will be injected subcutaneously twice daily for 5 consecutive days (= 1 cycle), every 8 weeks, for three cycles during Steps 1, 4, and 7. For dose modifications, see section 7.3.

There is a maximum of nine cycles of IL-2. For delaying or skipping cycles, see section 7.3. **At the discretion of the site PI, the timing of the IL-2 cycles can be adjusted +/- 2 weeks to accommodate extenuating circumstances. Subjects in Arm A (IL-2) who discontinue IL-2 injections for any reason can continue off treatment/on study with the approval of the protocol chair (see 8.2) and follow the same evaluations as scheduled for the Arm B subjects.**

IL-2 Cycle 1: Step 1, entry

IL-2 Cycle 2: Step 1, week 8

IL-2 Cycle 3: Step 1, week 16

IL-2 Cycle 4: Step 4, Registration to Step 4

IL-2 Cycle 5: Step 4, week +8

IL-2 Cycle 6: Step 4, week + 16

IL-2 Cycle 7: Step 7, Registration to Step 7

IL-2 Cycle 8: Step 7, week +8

IL-2 Cycle 9: Step 7, week +16

5.1.2 IL-2 Preparation

It is planned that rh-IL-2 4.5 MIU (vial labeled 0.44 mg/vial) vials will be provided for this study; however, in the case that 4.5 MIU vials are not available, 18 MIU (vial labeled 1.3 mg/vial) vials will be provided.

During reconstitution the SWFI should be directed at the side of the vial to avoid foaming and the contents of the vial should be gently swirled. The vial should not be shaken. To determine the volume of preservative free SWFI to be added see the Reconstitution Chart below.

| Dose of  rhIL-2 | Reconstitution Chart  FOR ALL VIAL SIZES – VALUES ROUNDED* | | |
| --- | --- | --- | --- |
|  | FOR 18 MIU (1.3 mg/vial)  Reconstitute lyophilized rhIL-2 with 1.2 mL SWFI that does not contain a preservative | FOR 4.5 MIU (0.44 mg/vial)  Reconstitute lyophilized rhIL-2  with 0.4 mL SWFI that does not contain a preservative | |
|  | Withdraw the corresponding volume of reconstituted rhIL-2 into a 1-mL syringe to obtain the desired dose. Injection volumes requiring a 0.05-mL increment are best achieved with a 0.5-mL syringe. | | |
| 1.5 MIU | 0.1 mL | | 0.1 mL |
| 3.0 MIU | 0.15 mL | | 0.15 mL |
| 4.5 MIU | 0.25 mL | | 0.25 mL |
| 6.0 MIU | 0.35 mL | | 0.35 mL *** |
| 7.5 MIU** | 0.4 mL | | 0.4 mL *** |

- - 1. The values presented have been rounded to the nearest **0**.05 mL and 0.5 MIU. This adjustment is not expected to result in dosage alterations of any clinical

consequence.

** 7.5 MIU is the maximum allowed dose.

*** Use two vials.

The resultant solution should be a clear, colorless to slightly yellow liquid. After reconstitution, store in a refrigerator at 2-8° C **(36-46°F)**. Do not freeze.

Preparation of IL-2 by qualified health care professionals under controlled and validated aseptic condition using a laminar flow hood: When reconstituted as directed, prepared syringes should be stored in the refrigerator at 2-8°C (36-46°F) and used within 14 days. Syringes that are dispensed should be labeled with a 14 day expiration. When preparing a dose in a syringe and administering the dose, consideration should be given to the volume of solution that may remain in the needle and the hub of the needle after the dose is administered. The pharmacy and clinic staff are encouraged to work together to administer the dose specified in the protocol.

Preparation of IL-2 by subjects and/or their caregivers: From a microbial standpoint, the product should be used immediately. The product contains no preservatives. Subjects should be instructed to use the number of vial(s) needed to prepare just one dose and to discard the remaining solution.

5.1.4 IL-2 Administration

Sites will provide training to subjects and/or their caregivers on the IL-2 self-administration technique. Subject education material on IL-2 administration and injection sites can be found on the A5102 protocol-specific Web page of the AACTG Web site. The first dose is to be administered in clinic with a 30-minute post-injection observation period. Subsequent doses may be administered at home by the subject or his/her caregiver once the injection techniques have been mastered. Subjects unable to master the self-injection technique may arrange to visit the site twice daily for injections. Caregivers and/or subjects should be instructed in the proper handling and disposal of syringes following treatment injections.

Syringes that have been refrigerated should be brought to room temperature 15 minutes prior to injection. The product should be inspected visually for particulate matter or discoloration. The solution should be clear, colorless to slightly yellow.

IL-2 is to be administered subcutaneously. Injection sites should be rotated. Cold compresses should be placed at the injection site 5 minutes before and after the injection to reduce swelling and discomfort. Subjects should be instructed to massage the injection site after IL-2 administration. Acetaminophen or ibuprofen may be taken as needed (see section 7.0, Toxicity Management).

### 5.2 IL-2 Product Formulation

Recombinant human interleukin-2 (IL-2, rhIL-2, rIL-2, Proleukin, and aldesleukin) 4.5 MIU (vial labeled 0.44mg/vial) vial is supplied as a sterile, white to off-white, lyophilized cake in single use 3 cc vials. When reconstituted with 0.4 mL SWFI, preservative free, each 0.25 mL contains 4.5 MIU rhIL-2, 12.5 mg mannitol and approximately 45 μg sodium dodecyl sulphate, buffered with approximately 42 μg sodium phosphate monobasic and 223 μg sodium phosphate dibasic monohydrate to a pH of 7.5 (range 7.2-7.8). If 4.5 MIU vials are not available, 18 MIU (vial labeled 1.3 mg/vial) vials will be provided. 18 MIU will be supplied as a sterile, white to off-white, lyophilized cake in single use 5 cc vials. When reconstituted with 1.2 ml SWFI, Preservative free, each 1 ml contains 18 MIU, 50 mg mannitol and approximately 180 μg sodium dodecyl sulphate, buffered with approximately 170 μg sodium phosphate monobasic and 890 μg sodium phosphate dibasic monohydrate to a pH of 7.5 (range 7.2-7.8). Store vials of lyophilized rhIL-2 in a refrigerator at 2-8°C (36-46°F). This product contains no preservative.

### 5.3 Product Supply, Distribution, and Pharmacy

5.3.1 Study Product Acquisition

IL-2 will be available through the NIAID Clinical Research Products Management Center. The ACTU pharmacist can obtain the study agent for this protocol by following the instructions in the manual, *Pharmacy Guidelines and Instructions for* ***D****AIDS Clinical Trials* ***Networks****,* in the section **Study Product Control.**

5.3.2 Study Supplies

Recombinant human interleukin-2 (Proleukin) will be provided by Chiron Corporation.

Antiretroviral medications will not be supplied through this study. See section 5.4.1 for a description of acceptable potent antiretroviral regimens for use in this study.

Supplies used for preparation and administration of IL-2 and disposal containers will not be provided through this study.

5.3.3 Investigational Agent Accountability

The ACTU pharmacist is required to maintain complete records of all study products received from the NIAID Clinical Research Products Management Center and subsequently dispensed. All unused study products must be returned to the NIAID Clinical Research Products Management Center after the study is completed or terminated. The procedures to be followed are provided in the manual, *Pharmacy Guidelines and Instructions for* ***D****AIDS Clinical Trials* ***Networks****,* in the section **Study Product Control.**

### 5.4 Concomitant Medications

5.4.1 Required Medications

Antiretroviral medications: Subjects must provide their own antiretroviral drugs. No restrictions will be placed on the potent antiretroviral drug combinations. However, it is strongly recommended that they involve FDA-approved medications used in accordance with the most recent DHHS treatment recommendations on potent ART available on the Web at

[**http://www.aidsinfo.nih.gov**](http://www.aidsinfo.nih.gov/)**.**

At the registration visits for Steps 1, 3, 4, 6, and 7, subjects will be instructed to bring in their potent ART medications for review by the clinic staff. Subjects will not be required to use the same potent ART throughout the study. However, any changes in potent ART must be recorded with the reason for the change in the source document and CRF.

Subjects on treatment regimens containing either nevirapine or efavirenz should stop these drugs 24-48 hours before stopping the other medications in the regimen at the end of both Steps 1 and 4.

5.4.2 Prohibited Medications for Arms A and B

- Antianginal medications
- Nitrates
- Calcium channel blockers. Examples of calcium channel blockers include: nifedipine (Adalat® /Procardia®), verapamil (Calan®/Isoptin®), diltiazem (Cardiazem®/Dilacor ®/Tiazac ™), amlodipine (Norvasc®/Rotrel®) and bepridil (Vascor®).
- Beta-blockers

NOTE: Calcium channel blockers or beta-blockers for isolated hypertension in subjects who do not have active cardiac disease is allowed.

- Antiarrhythmic agents
- Systemic or local toxic chemotherapy (local radiation is allowed).
- Astemizole (Hismanal)
- Midazolam (Versed)
- Triazolam (Halcion)
- Other cytokines except for erythropoietin
- Immunomodulatory therapy
- Systemic corticosteroids administered for ≥ 4 weeks
- Hydroxyurea

5.4.3 Precautionary Medications

- Iodinated contrast dye for subjects assigned to Arm A.

If a contrast exam with iodine is planned, it should be discussed with the study chair prior to initiation. Study participation or continuation on A5102 will be determined on a case-by-case basis.

### 5.5 Adherence Assessment

Subjects will be provided IL-2 adherence diaries, which will be reviewed by the site clinical staff at the next visit following the completion of each IL-2 cycle.

## 6.0 CLINICAL AND LABORATORY EVALUATIONS

Section 6.1 outlines the time points for the main study evaluations.

Section 6.2 outlines the time points for the substudy evaluations. The substudy evaluations occur only during Step 2 of the main study.

Sections 6.3 and 6.4 define the evaluations, provide timelines, and include special considerations or instructions for evaluations listed in the schedule of events.

### 6.1 Schedule of Events

| Evaluations | Screen | Preentry | Step 1  (Potent ART versus Potent ART + IL-2) | | | | | | | Register to  Step 2 | Step 2  (Treatment interruption)  (Refer to section 6.2 for substudy instructions) | | | |
| --- | --- | --- | --- | --- | --- | --- | --- | --- | --- | --- | --- | --- | --- | --- |
|  | Within 90 days prior to entry | Within 30 days prior to entry | Entry  Day 0 | Week 1 | Week 4 | Week 8 | Week 12 | Week 16 | Week 17 |  | Wk +2 | Wk +4 | Wk +8 | Wk +12 |
| HIV-1 ELISA Documentation | X |  |  |  |  |  |  |  |  |  |  |  |  |  |
| Medical/Medication History |  | X | X | X | X | X | X | X | X | X | X | X | X | X |
| Clinical Assessment |  | X | X | X | X | X | X | X | X | X | X | X | X | X |
| Targeted Physical Exam |  | X | X |  |  |  |  |  |  | X |  |  |  |  |
| Study Treatment Counseling |  |  | X |  |  |  |  |  |  | X |  |  |  |  |
| Hematology | X | X | X | X | X | X | X | X | X | X | X | X | X | X |
| Chemistry/Liver Function Tests |  | X | X | X |  | X |  | X |  | X |  |  | X |  |
| Fasting Metabolic Tests **(real time)** |  |  | X |  |  | X |  | X |  | X | X | X | X |  |
| **C-Reactive Protein (batched)a** |  |  | **X** |  |  |  |  | **X** |  |  |  | **X** | **X** |  |
| Modified Chemistry Panel (Arm A **only**) |  |  | X | X |  | X |  | X |  |  |  |  |  |  |
| HepC Ab and HepBsAg |  |  | X |  |  |  |  |  |  |  |  |  |  |  |
| TSH (Arm A only) |  |  | X | X | X | X | X | X | X |  |  |  |  |  |
| Pregnancy Testing |  | X | X |  |  |  |  |  |  | X |  |  |  |  |
| CD4/CD8 | X | X | X |  | X | X | X | X | X | X | X | X | X | X |
| Advanced Flow Panel |  |  | X |  |  |  |  | X |  |  | X | X | X |  |
| CCR5 Expression |  |  | X |  |  |  |  | X |  |  |  |  |  |  |
| **Soluble Markers of Activation (on stored specimen) a** |  |  |  |  |  |  |  |  |  | **X** |  |  |  |  |
| **Flow Cytometric Activation, Cell Turnover, and Apoptosis (on stored specimen) a** |  |  |  |  |  |  |  |  |  | **X** |  |  |  |  |
| Plasma HIV-1 RNA (real time) | X | X | X |  |  | X |  | X |  |  | X | X | X |  |
| Virus Cell Culture |  |  |  |  |  |  |  |  |  |  | X |  |  |  |
| **Resistance Testing (on stored specimen) a** |  |  |  |  |  |  |  |  |  |  | **X** | **X** | **X** |  |
| **Baseline DNA Quantification (on Case Western stored specimen only) a** |  |  | **X** |  |  | **X** |  | **X** |  |  |  |  |  |  |
| **Fitness Testing (on stored specimen) a** |  |  |  |  |  |  |  |  |  |  | **X** |  |  |  |
| Fasting Frozen Plasma, PBMCs, Serum and Urine **Collection and** Storage |  |  | X |  |  | X |  | X |  | X | X | X | X |  |
| Diary Distribution/Collection (Arm A only) |  |  | X | X |  | X | X | X | X |  |  |  |  |  |
| IL-2 Cycles (Arm A only) |  |  | X |  |  | X |  | X |  |  |  |  |  |  |
| Stop Potent ART |  |  |  |  |  |  |  |  |  | X |  |  |  |  |

**a No extra blood draw required.**

| Evaluation | Step 2 (Treatment Interruption)  After +24, Continue Q4 and Q8 weeks until  CD4+ < 350 cells/mm3 | | | | | | Register to Step Three | Step 3 (Potent ART Alone) | | | | | |
| --- | --- | --- | --- | --- | --- | --- | --- | --- | --- | --- | --- | --- | --- |
|  | Wk +16 | Wk +20 | Wk +24 | Q 4 Wks | Q 8 Wks | **End of Step 2** |  | Wk +4 | Wk +8 | Wk +12 | Wk +16 | Wk +20 | Wk +24 |
| Medical/Medication History | X | X | X | X |  |  | X | X | X | X | X | X | X |
| Clinical Assessment | X | X | X | X |  |  | X | X | X | X | X | X | X |
| Targeted Physical Exam |  |  |  |  |  |  | X |  |  |  |  |  |  |
| Study Treatment Counseling |  |  |  |  |  |  | X |  |  |  |  |  |  |
| Hematology | X | X | X | X |  |  | X | X | X | X | X | X | X |
| Chemistry/Liver Function Tests | X |  | X |  | X |  | X |  | X |  | X |  | X |
| Fasting Metabolic Tests **(real time)** | X |  | X |  | X |  | X |  | X |  | X |  | X |
| **C-Reactive Protein (batched) a** |  |  | **X** |  |  |  |  |  |  |  |  |  |  |
| Pregnancy Testing |  |  |  |  |  |  | X |  |  |  |  |  |  |
| CD4/CD8 | X | X | X | X |  |  | X | X | X | X | X | X | X |
| Advanced Flow Panel |  |  |  |  |  |  |  |  |  |  |  |  |  |
| CCR5 expression |  |  |  |  |  |  |  |  |  |  |  |  |  |
| **Soluble Markers of Activation**  **(on stored specimen) a** |  |  | **X** |  |  | **Xb** |  |  |  |  |  |  |  |
| **Flow Cytometry (on stored specimen) a** |  |  | **X** |  |  | **Xb** |  |  |  |  |  |  |  |
| Plasma HIV-1 RNA (real time) | X |  | X |  | X |  | X | X | X | X | X | X | X |
| **Stored Plasma for Virology a** |  |  |  |  |  |  | **X** |  | **X** |  | **X** |  | **X** |
| Virus Cell Culture |  |  |  |  |  |  |  |  |  |  |  |  |  |
| **Resistance Testing (on stored specimen) a** | **X** |  | **X** |  |  |  |  |  |  |  |  |  |  |
| **Baseline DNA Quantification (Case Western stored specimen only) a** |  |  |  |  |  |  |  |  |  |  |  |  |  |
| **Fitness Testing (on stored specimen) a** |  |  |  |  | **X** |  | **X** |  |  |  |  |  |  |
| Fasting Frozen Plasma, PBMCs, Serum and Urine **Collection and** Storage | X |  | X |  | X |  | X |  | X |  | X |  | X |
| Restart Potent ART |  |  |  |  |  |  | X |  |  |  |  |  |  |

**a No extra blood draw required.**

**b Measurement to be performed at the end of Step 2 when subject is determined to have CD4+ < 350 cells/mm3 on two consecutive visits.**

| Evaluation | | Register to  Step Four | | Step 4  (Potent ART versus Potent ART + IL-2) | | | | | | | | | | | | Register to Step Five | | Step 5 (Treatment Interruption)  After +24, Continue Q4 and Q8 until CD4+ < 350 cells/mm3 | | | | | | | | | | | | | | | | | |
| --- | --- | --- | --- | --- | --- | --- | --- | --- | --- | --- | --- | --- | --- | --- | --- | --- | --- | --- | --- | --- | --- | --- | --- | --- | --- | --- | --- | --- | --- | --- | --- | --- | --- | --- | --- |
|  | |  |  | Wk +1 | | Wk +4 | | Wk +8 | | Wk +12 | | Wk +16 | | Wk +17 | |  |  | Wk +2 | | Wk +4 | | Wk +8 | | Wk +12 | | Wk +16 | | Wk +20 | | Wk +24 | | Q 4 Wks | | Q 8 Wks | |
| Medical/Medication History | X | | X | | X | | X | | X | | X | | X | | X | | X | | X | | X | | X | | X | | X | | X | | X | |  | |  |
| Clinical Assessment | X | | X | | X | | X | | X | | X | | X | | X | | X | | X | | X | | X | | X | | X | | X | | X | |  | |  |
| Targeted Physical Exam | X | |  | |  | |  | |  | |  | |  | | X | |  | |  | |  | |  | |  | |  | |  | |  | |  | |  |
| Study Treatment Counseling | X | |  | |  | |  | |  | |  | |  | | X | |  | |  | |  | |  | |  | |  | |  | |  | |  | |  |
| Hematology | X | | X | | X | | X | | X | | X | | X | | X | | X | | X | | X | | X | | X | | X | | X | | X | |  | |  |
| Chemistry/Liver Function Tests | X | | X | |  | | X | |  | | X | |  | | X | |  | | X | | X | |  | | X | |  | | X | |  | | X | |  |
| Fasting Metabolic Tests  **(real time)** | X | |  | |  | | X | |  | | X | |  | | X | | X | | X | | X | |  | | X | |  | | X | |  | | X | |  |
| Modified Chemistry Panel  (Arm A only) | X | | X | |  | | X | |  | | X | |  | |  | |  | |  | |  | |  | |  | |  | |  | |  | |  | |  |
| TSH (Arm A only) | X | | X | | X | | X | | X | | X | | X | |  | |  | |  | |  | |  | |  | |  | |  | |  | |  | |  |
| Pregnancy Testing | X | |  | |  | |  | |  | |  | |  | | X | |  | |  | |  | |  | |  | |  | |  | |  | |  | |  |
| CD4/CD8 | X | |  | | X | | X | | X | | X | | X | | X | | X | | X | | X | | X | | X | | X | | X | | X | |  | |  |
| Advanced Flow Panel | X | |  | |  | |  | |  | | X | |  | |  | |  | |  | |  | |  | |  | |  | |  | |  | |  | |  |
| CCR5 Expression |  | |  | |  | |  | |  | | X | |  | |  | |  | |  | |  | |  | |  | |  | |  | |  | |  | |  |
| Plasma HIV-1 RNA (real time) | X | |  | |  | | X | |  | | X | |  | |  | | X | | X | | X | |  | | X | |  | | X | |  | | X | |  |
| **Stored Plasma for Virology a** | **X** | |  | |  | | **X** | |  | | **X** | |  | | **X** | | **X** | | **X** | | **X** | |  | | **X** | |  | | **X** | |  | | **X** | |  |
| Virus Cell Culture |  | |  | |  | |  | |  | |  | |  | |  | | X | |  | |  | |  | |  | |  | |  | |  | |  | |  |
| **Fitness Testing (on stored specimen) a** |  | |  | |  | |  | |  | |  | |  | | **X** | |  | |  | |  | |  | |  | |  | |  | |  | |  | |  |
| Fasting Frozen Plasma, PBMC, Serum and Urine **Collection and** Storage | X | |  | |  | | X | |  | | X | |  | | X | | X | | X | | X | |  | | X | |  | | X | |  | | X | |  |
| Diary Distribution / Collection  (Arm A only) | X | | X | |  | | X | | X | | X | | X | |  | |  | |  | |  | |  | |  | |  | |  | |  | |  | |  |
| IL-2 Cycles (Arm A only)**c** | X | |  | |  | | X | |  | | X | |  | |  | |  | |  | |  | |  | |  | |  | |  | |  | |  | |  |
| Stop Potent ART |  | |  | |  | |  | |  | |  | |  | | X | |  | |  | |  | |  | |  | |  | |  | |  | |  | |  |

**a No extra blood draw required.**

| Evaluation | Register to Step Six | Step 6  (Potent ART Alone) | | | | | | Register to Step Seven | Step 7  (Potent ART versus Potent ART + IL-2) | | | | | |
| --- | --- | --- | --- | --- | --- | --- | --- | --- | --- | --- | --- | --- | --- | --- |
|  |  | Wk +4 | Wk +8 | Wk +12 | Wk +16 | Wk +20 | Wk +24 |  | Wk + 1 | Wk +4 | Wk +8 | Wk +12 | Wk +16 | Wk +17 |
| Medical/Medication History | X | X | X | X | X | X | X | X | X | X | X | X | X | X |
| Clinical Assessment | X | X | X | X | X | X | X | X | X | X | X | X | X | X |
| Targeted Physical Exam | X |  |  |  |  |  |  | X |  |  |  |  |  |  |
| Study Treatment Counseling | X |  |  |  |  |  |  | X |  |  |  |  |  |  |
| Hematology | X | X | X | X | X | X | X | X | X | X | X | X | X | X |
| Chemistry/ Liver Function Tests | X |  | X |  | X |  | X | X | X |  | X |  | X |  |
| Fasting Metabolic Tests **(real time)** | X |  | X |  | X |  | X | X |  |  | X |  | X |  |
| Modified Chemistry Panel  (Arm A only) |  |  |  |  |  |  |  | X | X |  | X |  | X |  |
| TSH (Arm A only) |  |  |  |  |  |  |  | X | X | X | X | X | X | X |
| Pregnancy Testing | X |  |  |  |  |  |  | X |  |  |  |  |  |  |
| CD4/CD8 | X | X | X | X | X | X | X | X |  | X | X | X | X | X |
| Plasma HIV-1 RNA (real time) | X | X | X | X | X | X | X | X |  |  | X |  | X |  |
| **Stored Plasma for Virology a** | **X** |  | **X** |  | **X** |  | **X** | **X** |  |  | **X** |  | **X** |  |
| Virus Cell Culture |  |  |  |  |  |  |  |  |  |  |  |  |  |  |
| Fasting Frozen Plasma, PBMC, Serum and Urine **Collection and** Storage | X |  | X |  | X |  | X | X |  |  | X |  | X |  |
| Diary Distribution / Collection  (Arm A only) |  |  |  |  |  |  |  | X | X |  | X | X | X | X |
| IL-2 Cycles (Arm A only) |  |  |  |  |  |  |  | X |  |  | X |  | X |  |
| Restart Potent ART | X |  |  |  |  |  |  |  |  |  |  |  |  |  |

**a No extra blood draw required**

| **Evaluation** | **Step 8**  **(Observation)** | Study Termination or Premature Permanent  Discontinuation  **For Steps 1-7 only** |
| --- | --- | --- |
|  | **Q6 months until study termination** |  |
| **Medical/Medication History** | **X** | X |
| **Clinical Assessment** | **X** | X |
| **Targeted Physical Exam** | **X** | X |
| **Study Treatment Counseling** |  | X |
| **Hematology** |  | X |
| **Chemistry/Liver Function Tests** |  | X |
| **Fasting Metabolic Tests (real time)** |  | X |
| **Modified Chemistry Panel**  **(Arm A only)** |  |  |
| **TSH (Arm A only)** |  |  |
| **Pregnancy Testing** |  |  |
| **CD4/CD8** | **X** |  |
| **Plasma HIV-1 RNA (real time)** | **X** |  |
| **Stored Plasma for Virology a** |  | **X** |
| **Fasting Frozen Plasma, PBMC, Serum and Urine Collection and Storage** |  | X |
| **Diary Distribution /Collection**  **(Arm A only)** |  |  |

**aNo extra blood draw required**

### 6.2 Substudy Schedule of Events

Those subjects participating in the A5109s substudy must complete the following evaluations (see Appendix V**I**).

| Evaluations | A5102  Reg. to Step 2 | A5102  Step 2, week +4 | A5102  Step 2, week +8 |
| --- | --- | --- | --- |
|  | A5109s, week 0 | A5109s, week 4 | A5109s, week 8 |
| Modified Advanced Flow | X | X | X |
| Pro-inflammatory Cytokines | X | X | X |
| Intracellular Cytokines | X | X | X |
| Apoptosis by Propidium Iodide | X | X | X |
| LPA | X | X | X |

### 6.3 Definitions for Schedule of Events – Timing of Evaluations

6.3.1 Screening and Preentry

Screening and preentry evaluations occur prior to the subject taking any study medications, treatments, or interventions.

Screening: Screening evaluations must be completed within 90 days prior to study entry and no less than 48 hours prior to preentry.

Preentry: Preentry evaluations must be completed within 30 days prior to study entry and no less than 48 hours prior to entry.

6.3.2 On-Study Evaluations

Entry evaluations must be completed prior to starting study treatment. Subjects randomized to Arm A must begin treatment within 7 days of randomization. Evaluations must occur after randomization/registration. Study visits must occur on the time points indicated in the schedule of events ± 7 days, unless otherwise specified in the protocol.

**Note: Sites may elect to have an additional blood draw for Arm A subjects during Step 1 at weeks 9 and 17, and during Steps 4 and 7 at weeks +9 and +17 for optional safety chemistry evaluations.**

6.3.3 Steps

The study is divided into **8** steps. Subjects must randomize to Step 1 and then, if inclusion criteria are met, register to each subsequent step **up to Step 7**. **Subjects who complete Step 7 will not continue on to Step 8 but rather will complete the study termination evaluations as outlined in section 6.1.** Inclusion criteria are listed in section 4.0. Subjects will have up to 6 weeks to register to each subsequent step **(Step 2 through Step 7)** once completing the evaluations at the last visit of the previous step. Failure to register **to step 2 through 7** within 6 weeks **of the completion of the previous step will** result in study termination. **There is no time limitation for subjects to register to Step 8.**

Subjects on treatment regimens containing either nevirapine or efavirenz, should stop these drugs 24-48 hours before stopping the other medications in the regimen at the end of both Steps 1 and 4.

6.3.4 IL-2 Cycles

There is a maximum of 9 cycles of IL-2 for Arm A:

Cycle 1: Step 1, entry

Cycle 2: Step 1, week 8

Cycle 3: Step 1, week 16

Cycle 4: Step 4, Registration to Step 4

Cycle 5: Step 4, week +8

Cycle 6: Step 4, Week + 16

Cycle 7: Step 7, Registration to Step 7

Cycle 8: Step 7, week +8

Cycle 9: Step 7, week +16

NOTE: If either cycle 3, 6 or 9 is skipped, the evaluations associated with week 17 or +17 of that step are not required.

**For delaying or skipping cycles, see section 7.3.** **At the discretion of the site PI, the timing of the IL-2 cycles can be adjusted +/- 2 weeks to accommodate extenuating circumstances.**

6.3.5 Evaluations for Randomized Subjects Who Do Not Start Step 1

Subjects who do not start Step 1 will be considered off study and will not be included in the primary analysis. No further evaluations will be required for subjects who do not start Step 1.

**6.3.6 Evaluations for Subjects who Prematurely Discontinue Study Mandated Steps 1 through 7**

Subjects who prematurely discontinue from the study mandated steps before Step 7 may continue to be followed in Step 8 (see section 4.3.7 for criteria) until the last subject has completed following the protocol schema (Step 1 through Step 7). Those who do not enter Step 8 will complete the evaluations listed in the Schedule of Events for Study Termination or Premature Permanent Discontinuation (refer to Section 8.1).

**6.3.7 Evaluations for Subjects who Register to Step 8**

Subjects who register to Step 8 will complete the evaluations listed in the

**Schedule of Events for Step 8 until the last subject has completed following the protocol schema (Step 1 through Step 7).**

6.3.8 Study Termination

Evaluations should occur within 4 weeks from either completion of Step 7 or from reaching any of the criteria listed in section 8.1.

### 6.4 Special Instructions and Definitions of Evaluations

6.4.1 Informed Consent

A signed, dated, IRB-approved consent form is required prior to participation in this study.

6.4.2 Documentation of HIV Infection

HIV-1 infection, as documented by any licensed ELISA test kit and confirmed by Western blot at any time prior to study entry. A positive HIV-1 culture, HIV-1 antigen, plasma HIV-1 RNA, or a second antibody test by a method other than ELISA is acceptable as an alternative confirmatory test.

6.4.3 Medical History

- The medical history must be documented in the source document and CRF and updated at each visit. The medical history should include any previous HIV-related diagnoses and non-HIV-related diagnoses of major organ systems.
- Any allergies to any medications and their formulations must be recorded in the source document and updated at each visit.
- If available, the subject’s prior nadir CD4+ cell count (absolute value and date) must be documented with a copy of the nadir CD4+ cell count report and recorded in the source document and CRF. If a prior CD4+ nadir cell count is not available, this fact must also be documented in the source document and CRF.
- If available, the subject’s last documented viral load prior to initiating antiretroviral treatment after the original detection of HIV-1 infection must be recorded in the source document and the CRF. If two pre-treatment viral loads are available, both must be documented. The type of assay used must also be documented. A copy of the RNA report must be in the source document.
- If available, the subject’s highest viral load prior to initiating antiretroviral treatment after the original detection of HIV-1 infection must be recorded in the source document and the CRF.
- If available, the duration that the subject’s viral load has remained undetectable prior to entry must be documented in the source documents and CRF. Documentation will include the date of the first continuous undetectable viral load while the subject was on therapy that led up to the subject’s entry into A5102.
- If available, the subject’s documented history of hepatitis B surface antigen and/or hepatitis C serology positivity must be recorded in the source document and CRF.

6.4.4 Medication History

Source documentation for medication history must be present for:

- Nonprescription medications taken within 30 days prior to entry and updated at each visit. Include actual or estimated start and stop dates.
- Alternative therapies and dietary supplements taken within 30 days prior to entry. Include actual or estimated start and stop dates and update at each visit.

CRF and source documentation for medication history must be present for:

- Complete HIV treatment history, including start and stop dates of any antiretroviral medication (estimated if the exact dates cannot be obtained), immune-based therapy, or HIV-related vaccines, including blinded study medications.
- Complete treatment history of any prescription medications taken for the treatment or prophylaxis of OIs, including actual or estimated start and stop dates.
- All prescription medications including ARTs and St. John’s Wort taken within 30 days prior to entry into the study and updated at each visit. The source document and CRF must include start dates, stop dates, and dose changes.
- All modifications of antiretroviral drugs and IL-2 taken while the subject is on study including doses, subject-initiated and/or protocol-mandated interruptions, modifications, and permanent discontinuation of treatment will be recorded at each visit. Subject-initiated and protocol-mandated interruptions include both inadvertent and deliberate interruptions of IL-2 and/or ART.

6.4.5 Clinical Assessments

- Height: Obtained and recorded on the CRF at study entry only.
- Weight: Weight should be measured and recorded on the CRFs at the same visits that frozen samples are collected. The schedule is:

Step 1: Entry, weeks 8 and 16

Step 2: Registration to Step 2, weeks +2, +4, +8, +16, +24, +32 and Q 8 weeks (if applicable)

Step 3: Registration to Step 3, weeks +8, +16, +24

Step 4: Registration to Step 4, weeks +8 and +16

Step 5: Registration to Step 5, weeks +2, +4, +8, +16, +24, +32 and Q 8 weeks (if applicable)

Step 6: Registration to Step 6, weeks +8, +16, +24

Step 7: Registration to Step 7, weeks +8 and +16

Study Termination

- Signs and Symptoms: At entry, all signs and symptoms occurring within 30 days prior to study entry must be documented in the subject’s source document and CRF and must be updated at resolution. After entry, all signs and symptoms will continue to be documented in the source document but only those signs and symptoms Grade ≥ 3 that begin after entry must be recorded on CRFs. **However, in addition for Steps 1 through 7, record all signs and symptoms, regardless of grade, consistent with Retroviral Rebound Syndrome, as described under Diagnosis below, on the appropriate CRF.**

Refer to the Division of AIDS Table for Grading Adult Adverse Experiences, which can be found in the Protocol Management Handbook.

- Vital Signs: Temperature, pulse, blood pressure, and respiratory rate will be collected at all visits and recorded on the CRFs.
- Diagnoses: All confirmed and probable diagnoses made at entry and/or since the last visit will be recorded in the source documentation and CRFs, including current status at the time of study visit.

Each diagnosis must include:

1) Date of diagnosis and date of resolution (if known)

2) Method of confirmation of diagnosis or evidence for probable diagnosis.

**In addition, for Steps 1 through 7, retroviral rebound syndrome with date of diagnosis and date of resolution (if applicable) will be recorded in the source documentation and the appropriate CRF.**

**Retroviral rebound syndrome: Case definition must include the following:**

**1) Occurs within 8 weeks after cessation of ART.**

**2) Documented fever (temperature ≥ 38.5°C)**

**3) At least two major criteria**

**4) At least two minor criteria**

**5) No other cause for signs and symptoms identified**

**Major criteria:**

**A. Pharyngitis**

**B. Fatigue**

**C. Morbilliform rash**

**D. Myalgias/arthralgias**

**E. Lymphadenopathy**

**F. Subjective fever as reported by patient (may be intermittent)**

**Minor criteria:**

**G. Headache**

**H. Nausea/vomiting**

**I. Oral ulceration**

**J. Meningismus/aseptic meningitis**

**K. Night sweats**

**L. Thrombocytopenia (platelets <150,000/mm3)**

**M. Leukopenia (WBC <4,000/mm3)**

**N. Genital ulcers**

**O. Malaise**

**P. Abdominal pain**

**Q. Weight loss (loss of >5% of body weight)**

6.4.6 Targeted Physical Examination

A targeted physical examination is to be done at entry into the protocol and at registration to each step and is to be driven by any signs or symptoms previously identified that the subject has experienced. Each examination will also include a Karnofsky performance review. The targeted physical examination will be recorded on the CRF.

6.4.7 Study Treatment Counseling

At the registration visit for each step, the site clinical staff will provide counseling to each subject to review the subject’s treatment arm and step requirements including potent ART or IL-2 interruption or resumption. At each step, the clinical staff will document that the subject understands the procedures and treatment for each step in the subject’s source document. Prior to beginning each IL-2 cycle on Steps 1, 4, and 7, the clinical staff will distribute an IL-2 adherence diary to each subject in Arm A. The clinical staff will then collect and review the diaries upon completion of each IL-2 cycle. The information collected from the diaries will be recorded in the CRF.

6.4.8 Laboratory Evaluations

Prior to and at entry, record all laboratory values on the CRFs. After entry, record all Grade ≥ 3 laboratory values. Any laboratory values that lead to a change in treatment and all fasting metabolic values, regardless of grade, must also be recorded on the CRFs.

Refer to the Division of AIDS Table for Grading Adult Adverse Experiences, which can be found in the Protocol Management Handbook.

- Hematology: Hemoglobin, hematocrit, and WBC count with differential and platelets.
- Chemistry: Creatinine and serum amylase (with pancreatic amylase and/or lipase if amylase is elevated).
- Liver Function Tests: Total bilirubin, AST (SGOT), ALT (SGPT), and alkaline phosphatase.
- **Real Time** Fasting Metabolic Tests: Glucose, insulin, triglycerides, total cholesterol, and cholesterol fractions (LDL/HDL) to be done **in real time** at local sites.

NOTE: Fasting is defined as 8-12 hours since oral intake of anything other than water or medication. Metabolic tests need to be done fasting. Failure to fast will result in the visit being rescheduled within 7 days of the original visit date according to section 6.3.2.

- **Batched C-Reactive Protein Levels on Stored Samples: C-reactive protein levels will be determined from stored samples collected in section 6.4.11.**
- Modified Chemistry Panel (Arm A only): BUN, magnesium, total calcium, and electrolytes (chloride, sodium, potassium, bicarbonate).
- Thyroid Stimulating Hormone (TSH): Arm A only.
- Pregnancy Test (For women with reproductive potential): Serum or urine β-HCG (urine test must have a sensitivity of 25-50 μg). The test is to be repeated any time that pregnancy is suspected while the subject is on study. Subjects with a positive pregnancy test must permanently discontinue from study participation and complete the evaluations for study termination as described in section 6.3.6.
- Hepatitis C Ab and Hepatitis Bs Ag: If no documentation of a previous positive result is known, perform at entry and record on the CRF. If documentation of a previous positive result is available, the test is not required.

6.4.9 Immunologic Studies

- CD4+/CD8+ T-cell Lymphocytes: See section 6.4.3 regarding the nadir CD4+ cell count.

Obtain absolute CD4+/CD8+ cell counts and percentages within 90 days prior to entry (screening visit) and within 30 days prior to entry (preentry visit) from a **CLIA**-certified flow laboratory **or equivalent**. Eligibility for study participation will be determined by the screening CD4+ T-cell count.

CD4+ and CD8+ cell counts and subset percentage evaluations should be performed at **the same CLIA or equivalent and IQA-**certified flow laboratory throughout the study, if possible. Baseline calculation will be the average of the preentry and entry values.

Because of the diurnal variation in CD4+ and CD8+ cell counts, determinations for individual subjects should be obtained consistently in either the morning or the afternoon throughout the study, if possible.

NOTE: Each time a CD4+/CD8+ cell count measurement is obtained, the local laboratory must perform a WBC count and differential from a sample obtained at the same time.

- Advanced Flow Panel: Advanced flow analysis requires a CD4+/CD8+ cell count and WBC count with differential from a sample obtained at the same time. For more information, refer to Appendix I.

NOTE: Advanced Flow at registration to Step 2 is only required for subjects who are also enrolling on Substudy A5109s.

- CCR5 Expression: For detailed information, refer to Appendix I.
- **Soluble Markers of Activation: For detailed information, refer to Appendix I.**
- Flow Cytometric Determination of Activation, Cell Turnover and Apoptosis: For detailed information, refer to Appendix I.

6.4.10 Virologic Studies

- See section 6.4.3 regarding subject history of HIV-1 RNA.
- Plasma HIV-1 RNA, Real Time: Eligibility will be based on the screening and preentry values. Screening and preentry HIV-1 RNA PCR must be performed by a **CLIA**-certified laboratory. HIV-1 RNA PCR at other time points may be performed at any **CLIA and VQA**-certified virology laboratory.

HIV-1 RNA PCR at screening, preentry and at week +24 for Steps 3 and 6 must be performed using the UltraSensitive Roche Amplicor HIV-1 Monitor assay. At other time points, HIV-1 RNA PCR may be performed using either the Standard or UltraSensitive Roche Amplicor HIV-1 Monitor assays. Refer to Appendix II for more information.

- **Stored Plasma for Virology: Frozen specimens will be stored for possible future virologic determinations. For detailed information, refer to Appendix II.**
- Virus Cell Cultures: A 10-mL blood sample will be collected and sent same day to a VSL for virus culture at the week +2 visit in Steps 2 and 5. The blood must be kept at room temperature until processing and should be processed within 30 hours of collection. Planned tests will include an assessment of SI/NSI phenotype and production of a mini-stock using the quantitative PBMC micro culture assay.

For more information on virus cell cultures, refer to Appendix **IV**.

- **Resistance Testing: Will be performed to identify the frequency of resistance mutations in plasma RNA following treatment interruption. Samples will be used from the available specimens collected in section 6.4.11.**

**For more information on resistance testing, refer to Appendix III.**

- **Baseline DNA Quantification: Will be performed to estimate the level of HIV proviral DNA present in PBMCs during suppressive treatment. Samples will be used from the available specimens collected only by Case Western Reserve University according to section 6.4.11.**

**For more information on baseline DNA quantification, refer to Appendix III.**

- **Fitness Testing: Will be performed to determine if viral fitness near the initiation of treatment interruption correlates with CD4 trajectory over the course of Step 2. Samples will be used from the available specimens collected in section 6.4.11.**

**For more information on fitness testing, refer to Appendix III.**

6.4.11 Frozen Specimens: Plasma, PBMCs, Serum, and Urine

All study visits that require plasma to be frozen will be done FASTING. Fasting is defined as 8-12 hours since oral intake of anything other than water or medication.

Frozen specimens will be collected and logged into the LDMS for shipping to the central repository and stored for **planned and** possible future immunologic, virologic, endocrine, and metabolic studies. These specimens include PBMCs, plasma, serum, and urine.

A sample of the stored frozen samples will also be used for substudy testing (see 6.4.12).

**Appendix V lists all of the specimen collections.**

6.4.12 Substudy Assays

For more information on the substudy assays, refer to Appendix V**I**.

- Modified Advanced Flow

Consensus Advanced Flow panel and CD38/HLA-DR/CD4+

- Pro-inflammatory Cytokines
- Intracellular Cytokines

Interferon-γ production by flow cytometry after stimulation: **unstimulated (no stimulant), SEB, CMV, CMV control, p24, p24 control, HIV-1 MN, and HIV-1 MN control**

- Apoptosis by Propidium Iodide
- LPA (Lymphocyte Proliferation Assay)

Lymphocyte proliferative responses after stimulation with the core LPA stimulants (pokeweed mitogen [PWM], CMV, CMV control, and *Candida albicans* skin test antigen [CASTA™]), and HIV antigen.

### 6.5 Off-Drug Requirements

Additional safety monitoring and reporting of serious adverse experiences (SAEs) continues to be required upon completion or discontinuation of study treatment regardless of whether a protocol follow-up period is scheduled to occur. As specified in the current Division of AIDS *Serious Adverse Experience (SAE) Reporting Manual,* adverse experiences occurring during the immediate 8-week period after the last dose of study treatment which meet SAE reporting requirements must be reported to the Regulatory **Compliance Center** **Safety** Office. Additionally, after 8 weeks OFF study treatment, there are four types of events that must be reported to the **Safety** Office if the relationship to the study drug is assessed by the site physician as definitely, possibly, or unable to judge: DEATHS, NEW ONSET CANCERS, CONGENITAL ANOMALIES, AND PERMANENT DISABILITIES.

## 7.0 TOXICITY MANAGEMENT

Consult the most current version of the Adult ACTG Protocol Management Handbook for the DAIDS Toxicity Grading Table. The use of symptomatic medications during treatment with IL-2 is encouraged.

### 7.1 Symptom Specific Management

7.1.1 Flu-like Symptoms

7.1.1.1 Fever/arthralgia/myalgia: The onset of these symptoms usually occurs 2 to 4 hours after IL-2 administration. Administration of acetaminophen or non-steroidal anti-inflammatory agents (NSAIDS) is effective in controlling and preventing these symptoms. **The COX-2 inhibitors rofecoxib (Vioxx at 25‑50 mg/day) or celecoxib (Celebrex at 200 mg q day or 100 mg BID) have also been useful.**

7.1.1.2 Rigors/chills: These symptoms may occur 30 to 60 minutes after IL-2 administration. The agent of choice to improve severe chills or rigors is an opioid such as meperidine hydrochloride. If subjects develop rigors consistently after IL-2 administration, prophylactic administration of meperidine may prevent these reactions.

7.1.2 Hemodynamic Effects

7.1.2.1 Edema: Adequate hydration is necessary to ensure renal perfusion, but over hydration should be avoided. Subjects should be encouraged to drink electrolyte-containing fluids such as sport drinks and soups. If diuretics are to be used, they should be used with caution to avoid decreasing intravascular volume. Subjects should be reassured that they will lose any accumulated fluid after IL-2 administration stops.

7.1.2.2 Hypotension: There are data indicating that hypotension is observed in 6 to 14 percent of HIV infected persons dosed with intermittent courses of SC or CIV IL-2, although it is generally not severe. Organ dysfunction, oliguria, and increases in BUN and creatinine are almost always reversible upon discontinuation of IL-2, but on rare occasions, low doses of dopamine and fluid support may be warranted. Educating the subject to slowly rise from a supine to a sitting position and then to a standing position can prevent orthostatic hypotension.

7.1.3 Gastrointestinal Symptoms

7.1.3.1 Nausea/vomiting: Nausea and vomiting are common and usually occur 2 to 3 days after starting IL-2 administration. They tend to be mild to moderate and dose-dependent. Therapy with phenothiazines or serotonin receptor antagonists on an as-needed basis may prevent or ameliorate the symptoms. Other useful suggestions include telling subjects to eat frequent small meals and avoid spicy food.

7.1.3.2 Diarrhea: Antimotility agents such as loperamide hydrochloride can be helpful in treating IL-2 related diarrhea. Subjects should be encouraged to drink electrolyte-containing fluids should diarrhea develop.

7.1.3.3 Mucositis: Saline rinses may provide relief, and subjects should be encouraged to use a soft-bristled toothbrush. Alcohol-containing mouthwashes should be avoided. Subjects should also avoid hot or spicy foods and acidic fruits and juices. Suspensions containing antihistamines, an anesthetic, and an antacid have been used effectively to alleviate pain and discomfort.

7.1.4 Psychiatric Symptoms

7.1.4.1 Insomnia: Insomnia is a very commonly reported adverse effect of IL-2 administration but usually resolves 2 to 3 days after completing IL-2 administration. Benzodiazepines are effective in treating this side effect. Agents with significant CNS toxicity (as mentioned in section 5.4.2) such as triazolam are not permitted and should not be used to manage insomnia.

7.1.4.2 Anxiety: Subjects who experience anxiety during IL-2 administration may benefit from benzodiazepines and emotional support.

7.1.5 Dermatologic Reactions

7.1.5.1 Dry skin, pruritus, erythema, and sloughing occur commonly during IL-2 administration. No interruption of therapy is usually needed. Antihistamines, water or oil-based lotions, and oatmeal baths may help control the symptoms. Subjects should avoid using drying soaps and topical products containing alcohol and perfume. Subjects may experience the sensation of dry eyes, and artificial tears may provide relief.

7.1.5.2 Subcutaneous nodules: These nodules are transient, tend to occur at sites of IL-2 administration, and disappear weeks to months after therapy. To minimize injection site problems syringes should be brought to room temperature prior to administration of drug and cold compresses should be placed at the injection site 5 minutes before and after IL-2 injection. Subjects should be instructed to massage the injection site after IL-2 administration.

7.1.6 Respiratory Effects

Rhinitis is very commonly seen with IL-2 administration. Decongestants may be used.

### 7.2 Antiretroviral Drugs

Unanticipated and anticipated toxicities of antiretroviral drugs will be graded according to the Division of AIDS Table for Grading Adult Adverse Experiences, which can be found in the Protocol Management Handbook. All Grade 3 or 4 toxicities and any toxicity that results in a temporary or permanent change in an ART must be reported on the CRF.

Anticipated toxicities resulting from components of the antiretroviral drug regimen will be managed by the subject's clinician according to best clinical practice. Subjects will be allowed to change or switch components of their potent ART regimen. However, if potent ART is stopped completely during Steps 1, 3, 4, 6, or 7 due to an ART related toxicity, the subject must permanently discontinue study participation in A5102 and A5109s (if applicable).

### 7.3 IL-2 Dose Modifications

7.3.1 IL-2 Administration Modifications

7.3.1.1 If Grade 3 or 4 adverse events associated with IL-2 occur, IL-2 administration will be held until the toxicity decreases in severity to  Grade 2. However, once the IL-2 cycle is initiated, the dosing period will NOT be extended beyond 5 days (5 consecutive 24-hour periods) even for missed doses. The other clinical evaluations will continue as planned while the dose is being held.

7.3.1.2 Initiation of IL-2 may be delayed for up to 4 weeks for the management of acute intercurrent illnesses, toxicities arising from either ART or IL-2 or other reasons as approved by the protocol chairs. **The timing of the additional cycles of IL-2 can be adjusted +/- 2 weeks at the discretion of the site PI to accommodate extenuating circumstances.** If a cycle is delayed, all evaluations for that visit will also be delayed until the cycle is initiated. Subsequent visit weeks will be postponed by the same number of days that the current visit had been postponed. If the cycle is delayed beyond 4 weeks, the cycle will be skipped and the remainder of the clinical evaluations will be done at that time.

7.3.1.3 Once an IL-2 cycle is initiated, the dosing period will NOT be extended beyond 5 days (5 consecutive 24-hour periods) even for missed doses. Day 1 is the first day that IL-2 is administered. After day 5, regardless of whether or not the subject has completed 10 IL-2 doses for that cycle, the subject should continue on study per the evaluations and time points listed in section 6.0 and should not receive any additional IL-2 injections until the next cycle.

7.3.1.4 If an adverse event is regarded as a dose-limiting toxicity (DLT) in the investigator’s opinion and is a Grade 3 or 4, it will be managed as a DLT and the dose will be held until the toxicity decreases in severity to Grade ≤ 2. However, if the adverse event is regarded as a DLT by the investigator but is not a Grade 3 or 4, the dose will be held until the investigator determines that the severity of the toxicity has decreased enough for the subject to continue.

7.3.2 Dose Reduction of IL-2

7.3.2.1 When IL-2 is reinitiated during the same cycle after an interruption because of a DLT, the dose will be reduced by an increment of 1.5 MIU per injection (3 MIU/day). If the reduced dose results in a second DLT during the same cycle, IL-2 will be temporarily stopped and not reinitiated until the next cycle.

7.3.2.2 When IL-2 is reinitiated after having been temporarily stopped in a previous cycle due to one DLT, it is up to the discretion of the investigator at the site whether the new cycle will utilize the same reduced dose or the standard dose.

7.3.2.3 When IL-2 is reinitiated after having been temporarily stopped in a previous cycle due to two DLTs, the dose will be reduced again by an increment of 1.5 MIU per injection (3 MIU/day).

7.3.2.4 If a subject develops a toxicity that is subjectively intolerable but does not meet the criteria for a DLT, the dose of IL-2 can be lowered by 1.5 MIU per injection. The cycle does not need to be interrupted to decrease the dose in this circumstance.

7.3.2.5 If a subject cannot tolerate an IL-2 dose of 1.5 MIU twice daily, then IL-2 therapy will be permanently discontinued. The subject may continue on study following the same evaluations as scheduled for Arm B subjects after approval from the chair or vice chairs (see section 8.2).

7.3.3 Dose Increase of IL-2

7.3.3.1 If after a dose reduction a subject completes a treatment cycle without significant toxicity, then the IL-2 dose may be increased at the next cycle by an increment of 1.5 MIU/dose (3 MIU/day). The decision to increase the dose will be left to the discretion of the principal investigator at the site.

7.3.3.2 After the first two cycles of IL-2 in Steps 1, 4, and 7, subjects who do not have a CD4+ T-cell response at week 12 (week +12 of Steps 4 and 7) or at the visit following the completion of second IL-2 cycle, will be allowed to have their dose increased by 1.5 MIU/dose (3 MIU/day) during the same step contingent on the subject’s tolerance as defined above in sections 7.3.1.4 and 7.3.2.

A CD4+ T-cell response is defined as an increase of at least 20% in the CD4+ T-cell count above the step baseline and an increase of at least 50 cells/mL. The baseline visits for Steps 1, 4, and 7 are considered to be study entry, registration to Step 4, and registration to Step 7, respectively. The study entry/baseline CD4+ T-cell count is the average of the count from the preentry and entry visit.

7.3.3.3 Subjects’ IL-2 dose can be increased to 7.5 MIU BID (the maximum possible dose of as per section 5.1.2) if they have received at least two consecutive cycles of 6 MIU BID during the same step and still do not have a CD4+ T-cell response (as per section 7.3.3.2). This dose increase is also contingent upon subject tolerability.

## 8.0 CRITERIA FOR STUDY AND TREATMENT DISCONTINUATION

### 8.1 Premature and Permanent Study Discontinuation

**NOTE: Subjects who are no longer following the study mandated steps may continue on study for observation in Step 8 (see section 4.3.7).**

- Drug-related toxicity to the potent ART regimen requiring that potent ART be stopped completely during Steps 1, 3, 4, 6, or 7 (see section 7.0, Toxicity Management).
- **Development of any of the exclusionary conditions outlined in sections 4.2.5, 4.2.6, 4.2.9, 4.2.10, and 4.2.11 for study subjects assigned to Arm A (IL-2) only. Please note: These exclusionary conditions do not apply to subjects in Arm B.**
- Pregnancy during any step.
- Use of prohibited concomitant medications (see section 5.4).
- Failure to maintain a CD4+ cell count ≥ 500 cells/mm3 at the conclusion of Step 1 **without registration to Step 8**.
- Failure to achieve a CD4+ count ≥ 500 cells/mm3 at the conclusion of Step 4 **without registration to Step 8**.
- Reinitiation of potent ART prior to achieving a CD4+ T-cell count < 350 cells/mm3 on two consecutive visits during Steps 2 and 5 **without registration to Step 8**.
- Failure to achieve an HIV-1 RNA count < 200 copies/mL at week +24 of Steps 3 and 6 **without registration to Step 8**.
- Failure by the subject to attend two consecutive clinic visits (unless approved by the chair).
- Failure by the subject to register to each successive step **(Steps 2 through 7)** within 6 weeks from completing the evaluations on the last visit of the previous step.
- Request by the subject to withdraw.
- Request of the primary care provider if s/he thinks the study is no longer in the best interest of the subject.
- Clinical reasons believed life threatening by the physician, even if not addressed in the toxicity management section of the protocol.
- Subject is judged by the investigator to be at significant risk of failing to comply with the provisions of the protocol so as to cause harm to self or seriously interfere with the validity of the study results.
- At the discretion of the AACTG, the FDA, the National Institute of Allergy and Infections Diseases (NIAID), the investigator, or the pharmaceutical sponsor.
- Completion of Step 7 **or Step 8**.

### 8.2 Premature and Permanent IL-2 Discontinuation (Arm A Only)

Subjects who discontinue IL-2 **injections for any reason** but who wish to remain on study may do so with the approval of the study chair. Those subjects can remain on study, off treatment for the remainder of the study following the same evaluations as scheduled for Arm B subjects.

## 9.0 STATISTICAL CONSIDERATIONS

### 9.1 General Design Issues

A5102 is an open-label, pilot, multicenter, multistep study randomizing subjects to +/- IL-2. The study is designed to evaluate the intercept and slope of CD4+ T-cell counts during Step 2 in HIV-infected individuals who have been treated with stable potent ART for at least 3 months prior to enrollment and have CD4+ T-cell counts ≥ 500 cells/mm3 and HIV-1 RNA < 400 copies/mL at screening and plasma HIV-1 RNA < 200 copies/mL at preentry. Potent ART will be initiated and interrupted, based on CD4+ T-cell counts, as an alternative approach to the chronic management of HIV infection.

Eighty eligible subjects will be randomized with equal probability to two treatment groups (with and without IL-2).

Subjects also will be stratified based on their nadir CD4+ T-cell count (no nadir count,  200 cells/mm3 and > 200 cells/mm3) to ensure that a balance between persons with low nadir CD4+ T-cell counts exists between study arms. The A5102 team would like to explore further published data that suggest response to ART is worse for persons with a CD4+ T-cell count < 200 cells/mm3.

### 9.2 Endpoints

9.2.1 Primary Endpoint

9.2.1.1 Slope and intercept of the CD4+ T-cell count during Step 2.

9.2.2 Secondary Endpoints

9.2.2.1 Time from antiretroviral treatment interruption (start of Step 2) to the first of two consecutive CD4+ T-cell count determinations < 350 cells /mm3.

9.2.2.2 Whether or not a subject has an HIV-1 RNA level < 200 copies/mL at the end of Step 3/4 and Step 6/7.

9.2.2.3 Whether or not a subject has a CD4+ T-cell count ≥ 500 cells/mm3 at the end of Step 1, Step 3/4 and Step 6/7.

9.2.2.4 Slope of HIV-1 RNA levels during Steps 2 and 5 (treatment interruption steps).

9.2.2.5 Whether or not a subject has a CD4+ T cell count < 350 cells/mm3 after +24 weeks in Step 2 or Step 5.

9.2.2.6 Slope of CD4+ T-cell counts from Steps 2 and 5 (treatment interruption).

9.2.2.7 Change **in the following metabolic testing markers for each step:**

**a. Lipid Parameters**

**1. total cholesterol**

**2. LDL/HDL cholesterol**

**3. triglycerides**

**4. C-reactive protein**

**b. Glucose Parameters**

**1. glucose**

**2. insulin**

**9.2.2.8 Virologic and Resistance Testing Markers**

**a. Level of proviral HIV-1 DNA from Step 1 at weeks 0, 8, and 16.**

b. Genotypic markers of resistance at **the first HIV-1 RNA > 500 copies/mL, at the plateau or maximal value of HIV-1 RNA on** Steps 2 and 5 and study completion, as well as at Steps 3 and 6 for those subjects who failed to successfully complete Steps 3 or 6.

c. Fitness testing marker

**9.2.2.9 Immune activation markers**

**a. Soluble markers of activation: plasma TNFRII and ICAM-1 at Step 2 baseline and week +24**

**b. Cellular markers of immune activation at Step 2 baseline and week +24:**

- - **CD38+CD70+CD4+**
  - **CD38+CD70+CD8+**
  - **Ki-67+CD4+**
  - **Ki-67+CD8+**

9.2.2.10 Effect of treatment interruption on the proportion of naive and memory CD4+ cells.

9.2.2.11 Safety and tolerability.

### 9.3 Substudy Endpoints

9.3.1 Primary Endpoint

9.3.1.1 Change in LPA (measured by net CPM) after stimulation with CMV, and HIV antigens from the substudy entry to week +8 of Step 2.

9.3.2 Secondary Endpoint

9.3.2.1 Effect of treatment interruption on the proportion of naive and memory CD4+ cells, change in the proportion of cells expressing CD38, and change in the proportion of CD4+ and CD8+ lymphocytes.

9.3.2.2 Intracellular cytokines marker: INF-gamma responses by flow cytometry after stimulation with CMV and HIV antigens.

Change in the proportion of cells in S phase of cell cycle from the substudy entry evaluation to week +8 of Step 2.

### 9.4 Sample Size and Accrual

Forty subjects will be enrolled in each arm. Subjects who do not start Step 2 will not be included in the primary analysis and will be considered off study.

**9.4.1 Sample Size Consideration for Primary Endpoint**

We assume 10% of the subjects will be lost to follow-up or off study before they enter into Step 2. Given 36 evaluable subjects per arm, this study will be able to estimate the slope of CD4+ T-cell in Step 2 with a 95% confidence interval width of 0.65 standard deviation of the distribution of slope in each arm. Accrual rate will be approximately 14 subjects per month, thus this study will be fully enrolled in 6 months. **Due to slower than expected accrual into the study, the team decided to close the study to further accrual effective January 19, 2003.**

**9.4.2 Sample Size Consideration for Step 2 Week +24 Metabolic Endpoint Change from Baseline**

**According to the current database, the A5102 frozen specimen tracking form indicates that 35 subjects had Step 2 baseline and week +24 of treatment interruption fasting blood samples stored. In addition, 3 subjects had baseline fasting blood samples but they have not yet reached week +24, so there are a total of 38 subjects expected to have both baseline and week +24 data available.**

**With 19 subjects within each group, the study has 80% power to detect a difference between Step 2 baseline and week +24 C-reactive protein at a two- sided 5% significance level if the true difference is 0.75 of the standard deviation.**

**All other metabolic endpoints have already been collected at the study evaluations. For example, fasting total cholesterol exists in the database at Step 2 baseline and Step 2 week +24 for 35 subjects, plus there are 3 subjects who have not yet reached week +24 evaluations. There are a total of 38 subjects expected to have baseline and week +24 total cholesterol data available.**

**With 19 subjects within each group, the study has 80% power to detect a difference between Step 2 baseline and week +24 total cholesterol level at a two-sided 5% significance level if the true difference is 4.87% of the baseline. This is based on the assumption that the standard deviation of the difference is 6.5% of the baseline (85). If the standard deviation is twice the size of that in Dybul's paper (SD=13%), the study has 80% power to detect a difference between Step 2 baseline and week +24 total cholesterol level if the true difference is 9.7% of the baseline.**

**9.4.3 Sample Size Consideration for Step 2 Baseline Proviral DNA**

**The study has 80% power to detect a correlation between Step 2 CD4+ slope and baseline proviral DNA with 19 subjects in each arm. This is based on a two-sided 5% significance level, if the true correlation coefficient is less than 0.618 (negative correlation) or greater than 0.618 (positive correlation).**

**NOTE: Only summary statistics will be performed on resistance and fitness testing.**

**9.4.4 Sample Size Consideration for Step 2 Baseline Immune Activation Marker**

**The study has 80% power to detect a correlation between Step 2 CD4+ slope and baseline immune activation marker with 19 subjects in each arm. This is based on a two-sided 5% significance level, if the true correlation coefficient is less than -0.618 (negative correlation) or greater than 0.618 (positive correlation).**

### 9.5 Substudy Sample Size and Accrual

Sixteen subjects, approximately 8 from each arm, will be enrolled into the substudy. Data will be summarized by treatment group. An attempt will be made to substitute subjects who drop out of the study to have at least 7 evaluable subjects in each arm. **Due to the slower than expected accrual the substudy was closed to further accrual effective January 19, 2003 (11 of 16 planned subjects were enrolled).**

### 9.6 Analysis

9.6.1 Interim Safety Analysis

An independent committee will review safety and accrual results of A5102 at six and 12 months after the study opens, at 12 months after the last subject enrolls in Step 2, and yearly until the study closes. The committee will consist of the protocol statistician, protocol DAIDS clinical representative, an immunologist, and two members of the AACTG Immunology Research Agenda Committee (IRAC) who are not connected to this study. The committee will be given data on plasma RNA levels, accrual, safety laboratory results, and adverse events. **At the time A5102 Version 2.0 was written, two interim safety analyses had been completed with no recommendations to change the study.**

The A5102 team, independent of the review committee, will close an arm of the study if ≥ 50% of subjects enrolled in that arm (i.e. 20 subjects in an arm) experience either a decline in their CD4+ T-cell count to < 350 cells/mm3 or an HIV-1 RNA rebound to > 100,000 copies/mL within 6 months of entering Step 2 or 5.

9.6.2 Primary Analysis

**There are a total of 46 subjects registered to Step 2. The last subject started Step 2 six months after the 45th subject entered Step 2. Therefore the team will initiate the primary analysis 6 months after the 45th subject has entered into Step 2, which is approximately July 6, 2003. The team plans to include more follow-up data from the last subject in subsequent analyses.**

**The primary analysis will use all subjects’ Step 2 data to determine the CD4+ Tukey ladder power transformation that best results in a linear relationship between the transformed CD4+ count and time and also normality of CD4+ errors.**

**For each subject, we will use the CD4+ transformation obtained above and fit a linear model to assess the subject’s CD4+ decline. The least square estimate of slope and intercept of transformed CD4+ count on Step 2 will be calculated and the average transformed CD4+ slope and intercept will be summarized by treatment group. We will explore some nonlinear models if no transformation of CD4+ count will fit a linear model.**

**In order to explore the associations between other covariates of interest (proviral DNA copies during Step 1, slope or other appropriate parameters for RNA trajectory in Step 2, HIV-1 RNA resistance, immune markers and other laboratory markers at entry and during Step 2) and the Step 2 CD4+ trajectory, the correlation between covariates of interest and transformed CD4+ slope will be estimated and evaluated with a Spearman rank correlation estimate and test.**

**Based on the selected CD4+ transformation, we will estimate the time from baseline of Step 2 to CD4+ count decline below 350 cells/mm3 for each subject and provide the median and 95% confidence interval on this time for each group.**

9.6.2.1 Virologic/Resistance Studies

9.6.2.1.1 Baseline DNA Quantification

**To estimate the level of HIV-1 proviral DNA present in PBMC during suppressive treatment, PBMC samples from Step 1 weeks 0, 8, and 16 will be used. Wilcoxon signed rank test on change from weeks 0, 8, and 16 DNA level by treatment group will be performed. If there is no significance between weeks 0, 8, and 16 HIV-1 proviral DNA for both arms, the average of the measurements will be used as the baseline DNA value. Otherwise, the week 16 data on Step 1 will be considered as baseline DNA value. The correlation between baseline DNA data and Step 2 CD4+ trajectory will be evaluated.**

**9.6.2.1.2 Resistance Testing**

**The frequency of resistance mutations in plasma RNA at the first HIV plasma sample with HIV-1 RNA > 500 copies/mL on Step 2 and the consensus RNA genotype data will be summarized by treatment group, ART category, and by each antiretroviral drug if necessary. The sequences obtained at the earliest detection of RNA will be compared to those in the “final” plasma sample obtained at the plateau or maximal value of HIV-1 RNA on Step 2 (off drug). Among individuals with resistance mutations at early escape RNA (Step 2 week +2, +4) resistance data at intervening samples will be analyzed to estimate the rate of reversion to wild type.**

**9.6.2.1.3 HIV-1 RNA Trajectories in Step 2**

**The rate of re-emergence of viremia, and measures of in vivo fitness of the emergent HIV plasma RNA will be assessed by analysis of HIV-1 RNA measures in Step 2. The level of plasma RNA during Step 2 week +2, +4, +8, +16, and +24 (6 months on Step 2) will provide four measures:**

**1) Time from interruption to detection > 500 copies/mL. Kaplan-Meier plots will evaluate time to detectable plasma RNA by treatment arm, baseline proviral DNA level, and pre-treatment plateau RNA levels.**

**2) A slope of HIV-1 RNA.**

**3) Absolute magnitude of “plateau” defined as two repeated RNA measure within 0.3 log/copies/mL, and if known, comparison to pre-treatment baseline.**

**4) The area under the curve of virus replication during the first 6 months of Step 2.**

**9.6.2.2 Metabolic Studies**

**For the purpose of this preliminary analysis, samples from Step 1 week 0 and 16 will be analyzed to determine baseline values. Wilcoxon signed rank test by treatment arm will be performed to test the hypothesis that there is no difference between week 0 and week 16 marker values on Step 1. If the differences between Step 1 week 0 and week 16 values are not significant in both arms, then the average of the two measurements will be used as baseline value for Step 2. Otherwise, if significant difference between Step 1 week 0 and week 16 is found in any treatment arm, week 0 value of Step 1 will be used as the baseline for Step 2 for both treatment groups.**

**Data from baseline and all time points during Step 2 will be analyzed (week + 2, + 4, + 8, + 16, +24, and q8 weeks on Step 2) to evaluate the rate and overall magnitude of changes in metabolic parameters. Changes from baseline at each time point on Step 2 will be summarized and analyzed using Wilcoxon signed rank test by treatment arm. Longitudinal data analysis for Step 2 metabolic markers will be explored.**

**9.6.2.3 Immune Activation Marker**

**Change from Step 2 baseline to week +24 for immune activation markers will be tabulated by treatment group. The correlation between Step 2 baseline immune activation marker and Step 2 CD4+ trajectory will be evaluated.**

- - 1. Secondary Analysis

Secondary analysis will be conducted after all subjects have completed the study and will include the following:

- Kaplan-Meier estimate of median time to first of the two consecutive CD4+ cell count determinations < 350 cells/mm3 by treatment group;
- Summary of the least square estimate of CD4+ slope in Step 5;
- Treatment comparison on slope of CD4+ cell count in Step 2 and Step 5 by a two-sided Wilcoxon test;
- Frequency table and two-sided Fisher’s exact test on proportion of subjects with CD4+ T-cell count > 500 cells/mm3 at the end of Step 1, Step 3 / 4, and Step 6/7;
- Frequency table and two-sided Fisher’s exact test on proportion of subjects with CD4+ T-cell counts < 350 cells /mm3 after +24 weeks in Step 2 and Step 5;
- Summary of the least square estimate of slope of log10 HIV-1 RNA level in Step 2 and Step 5 by treatment group;
- Treatment comparison on slope of log10 HIV-1 RNA level in Step 2 and Step 5 by two-sided Wilcoxon test;
- Frequency table and two-sided Fisher’s exact test on proportion of subjects with HIV-1 RNA level below 200 copies/mL at the end of Step 3/4 and Step 6/7;
- Summary statistics and two-sided Wilcoxon test on change of glucose and insulin level in each step;
- Summary statistics and two-sided Wilcoxon test on change of cholesterol and tryglyceride level in each step;
- Frequency table and two-sided Fisher’s exact test on proportion of subjects with Grade ≥ 3 toxicities;
- Summary statistics of the predictors (nadir CD4+ count, baseline and peak viral load, length of ART, type of ART, duration of undetectability while on treatment, and demographic variables) of CD4+ decline**;**
- **Summary statistics and two-sided Wilcoxon test on change of C- reactive protein level in each step.**

## 10.0 COLLECTION AND MONITORING AND ADVERSE EXPERIENCE REPORTING

### 10.1 Records to Be Kept

Case report forms (CRFs) will be provided for each subject. Subjects must not be identified by name on any CRF. Subjects will be identified by the Patient Identification Number (PID) and Study Identification Number (SID) provided by the ACTG Data Management Center upon randomization. New SIDs will be assigned to subjects at each step registration.

### 10.2 Role of Data Management

10.2.1 Instructions concerning the recording of study data on CRFs will be provided by the ACTG Data Management Center. Each ACTU is responsible for keying the data in a timely fashion.

10.2.2 It is the responsibility of the ACTG Data Management Center to assure the quality of computerized data for each ACTG study. This role extends from protocol development to the generation of the final study databases.

### 10.3 Clinical Site Monitoring and Record Availability

10.3.1 Site monitors under contract to the NIAID will visit participating clinical research sites to review the individual subject records, including consent forms, CRFs, supporting data, laboratory specimen records, and medical records (physicians’ progress notes, nurses’ notes, individuals’ hospital charts), to ensure protection of subjects, compliance with the protocol, and accuracy and completeness of records. The monitors also will inspect sites’ regulatory files to ensure that regulatory requirements are being followed and sites’ pharmacies to review product storage and management.

10.3.2 The investigator will make study documents (e.g., consent forms, drug distribution forms, CRFs) and pertinent hospital or clinic records readily available for inspection by the local IRB, the site monitors, the FDA, the NIAID, the Office for Human Research Protections (OHRP, formerly OPRR), the pharmaceutical sponsor, or the sponsor’s designee for confirmation of the study data.

### 10.4 Serious Adverse Experience (SAE) Reporting

This protocol follows standard reporting requirements.

Serious adverse experiences associated with any antiretroviral drugs or to IL-2 must be documented on the Division of AIDS Serious Adverse Experience (SAE) reporting form and submitted to the Regulatory **Compliance Center** **Safety** Office. Serious adverse events are defined in the current Division of AIDS (DAIDS) *Serious Adverse Experience (SAE) Reporting Manual.* The PID should be used in all SAE reporting.

The Division of AIDS Table for Grading Adult Adverse Experiences is located in the Protocol Management Handbook **or at the RCC Website:** [**http://rcc.tech-res-intl.com**](http://rcc.tech-res-intl.com/)**.**

## 11.0 HUMAN SUBJECTS

### 11.1 Institutional Review Board (IRB) Review and Informed Consent

This protocol, the informed consent document (Appendix VI**I**) and any subsequent modifications will be reviewed and approved by the IRB or ethics committee responsible for oversight of the study. A signed consent form will be obtained from the subject (or parent, legal guardian, or person with power of attorney for subjects who cannot consent for themselves). The subject's assent must also be obtained if he or she is able to understand the nature, significance, and risks of the study. The consent form will describe the purpose of the study, the procedures to be followed, and the risks and benefits of participation. A copy of the consent form will be given to the subject, parent, or legal guardian, and this fact will be documented in the subject’s record.

### 11.2 Subject Confidentiality

All laboratory specimens, evaluation forms, reports, and other records that leave the site will be identified by coded number only to maintain subject confidentiality. All records will be kept locked. All computer entry and networking programs will be done with coded numbers only. Clinical information will not be released without written permission of the subject, except as necessary for monitoring by IRB, the FDA, the NIAID, the pharmaceutical sponsor, or the sponsor’s designee.

### 11.3 Study Discontinuation

The study may be discontinued at any time by the IRB, the NIAID, the pharmaceutical sponsor, the FDA, or other government agencies as part of their duties to ensure that research subjects are protected.

## 12.0 PUBLICATION OF RESEARCH FINDINGS

Publication of the results of this trial will be governed by ACTG policies. Any presentation, abstract, or manuscript will be made available for review by the pharmaceutical sponsors prior to submission.

## 13.0 BIOHAZARD CONTAINMENT

As the transmission of HIV and other blood-borne pathogens can occur through contact with contaminated needles, blood, and blood products, appropriate blood and secretion precautions will be used by all personnel in the drawing of blood and shipping and handling of all specimens for this study, as currently recommended by the Centers for Disease Control and Prevention and the National Institutes of Health (NIH).

All infectious specimens will be transported using packaging mandated in the Code of Federal Regulations, CDC 42 CFR Part 727. Please refer to individual carrier guidelines, e.g., FedEx, Airborne, for specific instructions.

## 14.0 REFERENCES

1. Palella FJ, Jr., Delaney KM, Moorman AC, et al. Declining morbidity and mortality among patients with advanced human immunodeficiency virus infection. HIV Outpatient Study Investigators. N Engl J Med 1998;338(13):853-60.

2. Anonymous. Update: trends in AIDS incidence -- United States, 1996. Morbidity & Mortality Weekly Report 1997;46(37):861-7.

3. Furrer H, Egger M, Opravil M, et al. Discontinuation of primary prophylaxis against Pneumocystis carinii pneumonia in HIV-1-infected adults treated with combination antiretroviral therapy. N Engl J Med 1999;340:1301-6.

4. Weverling GJ, Mocroft A, Ledergerber B, et al. Discontinuation of Pneumocystis carinii pneumonia prophylaxis after start of highly active antiretroviral therapy in HIV-1 infection. Lancet 1999;353:1293-8.

5. Schneider MME, Borleffs JCC, Stolk RP, Jaspers CAJJ, Hoepelman AIM. Discontinuation of Pneumocystis carinii pneumonia prophylaxis in HIV-1 infected patients treated with highly active antiretroviral therapy. Lancet 1999;353:201-3.

6. Dworkin M, Hanson D, Jones J, Kaplan J, and the Adult/Adolescent Spectrum of HIV Disease Project (ASD). The risk for Pneumocystis carinii pneumonia (PCP) and disseminated non-tuberculous Mycobacteriosis (Dmb) after an antiretroviral therapy (ART) associated increase in the CD4+ T-lymphocyte count [Abstract 692]. In: Program and Abstracts: 6th Conference on Retroviruses and Opportunistic Infections. Alexandria, VA: Foundation for Retrovirology and Human Health, 1999.

7. Lopez JC, Pena JM, Miro JM, Podzamczer D, and the GESIDA 04/98 Study Group. Discontinuation of PCP prophylaxis (PRO) is safe in HIV-infected patients (PTS) with immunological recovery with HAART. Preliminary results of an open, randomized and multicenter clinical trial (GESIDA 04/98) [Abstract LB7]. In: Program and Abstracts: 6th Conference on Retroviruses and Opportunistic Infections. Alexandria, VA: Foundation for Retrovirology and Human Health, 1999.

8. MacDonald JC, Torriani FJ, Morse LS, Karavellas MP, Reed JB, Freeman WR. Lack of reactivation of cytomegalovirus (CMV) retinitis after stopping CMV maintenance therapy in AIDS patients with sustained elevations in CD4 T cells in response to highly active antiretroviral therapy. J Infect Dis 1998;177:1182-7.

9. Tural C, Romeu J, Sicrera G, et al. Long-lasting remission of cytomegalovirus retinitis without maintenance therapy in human immunodeficiency virus-infected patients. J Infect Dis 1998;177:1080-3.

10. Vrabec TR, Baldassano VF, Whitcup SM. Discontinuation of maintenance therapy in patients with quiescent cytomegalovirus retinitis and elevated CD4+ counts. Ophthalmology 1998; 105:1259-64.

11. Lucas GM, Chaisson RE and Moore RD. Highly active antiretroviral therapy in a large urban clinic: risk factors for virologic failure and adverse drug reactions. Ann Intern Med 1999; 131:81-7.

12. Visnegarwala F, Krause KL, Musher DM. Severe diabetes associated with protease inhibitor therapy. Ann Intern Med 1997;127(10):947.

13. Eastone JA, Decker CF. New-onset diabetes mellitus associated with use of protease inhibitor. Ann Intern Med 1997;127(10):948.

14. Lo JC, Mulligan K, Tai VW, Algren H, Schambelan M. "Buffalo hump" in men with HIV-1 infection. Lancet 1998;351(9106):867-70.

15. Deeks SG, Smith M, Holodniy M, Kahn JO. HIV-1 protease inhibitors. A review for clinicians. JAMA 1997;277(2):145-53.

16. Brinkman K, ter Hofstede HJ, Burger DM, Smeitink JA, Koopmans PP. Adverse effects of reverse transcriptase inhibitors: mitochondrial toxicity as common pathway. AIDS 1998;12(14):1735-44.

17. Carr A, Samaras K, Thorisdottir A, Kaufmann GR, Chisholm DJ, Cooper DA. Diagnosis, prediction, and natural course of HIV-1 protease-inhibitor-associated lipodystrophy, hyperlipidaemia, and diabetes mellitus: a cohort study. Lancet 1999;353(9170):2093-9.

18. Behrens G, Dejam A, Schmidt H, et al. Impaired glucose tolerance, beta cell function and lipid metabolism in HIV patients under treatment with protease inhibitors. AIDS 1999;13(10):F63-70.

19. Henry K, Melroe H, Huebsch J, et al. Severe premature coronary artery disease with protease inhibitors. Lancet 1998;351(9112):1328.

20. Yarasheski KE, Tebas P, Sigmund C, et al. Insulin resistance in HIV protease inhibitor-associated diabetes. JAIDS 1999;21(3):209-16.

21. [Guidelines for the Use of Antiretroviral Agents in HIV-Infected Adults and Adolescents](http://hivinsite.ucsf.edu//medical/tx_guidelines/2098.3a62.html). Department of Health and Human Services, updated **November 10, 2003**. ([www.**aidsinfo.nih.gov**](http://www.aidsinfo.nih.gov/))

22. Carpenter CC, Cooper D, Fischl MA, et al. Updated recommendations of the International AIDS Society-USA Panel. JAMA 2000;283:381-90.

23. Finzi D, Hermankova M, Pierson T, et al. Identification of a reservoir for HIV-1 in patients on highly active antiretroviral therapy. Science 1997;278:1295
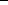
300.

24. Kovacs JA, Baseler M, Dewar RJ, et al. Increases in CD4 T lymphocytes with intermittent courses of interleukin-2 in patients with human immunodeficiency virus infection. A preliminary study. N Engl J Med 1995; 332:567
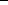
75.

25. Kovacs JA, Vogel S, Albert JM, et al. Sustained increases in CD4 counts with intermittent interleukin-2 therapy in HIV-infected patients with greater than 200 CD4 cells/mm3: results of a randomized, controlled trial. N Engl J Med 1996; 335:1350
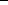
6.

26. Carr A, Lloyd A, Emery S, et al. A randomized, controlled trial of rhIL-2 (IL-2) or polyethylene glycol rhIL-2 (PEG-IL-2) plus antiretroviral therapy (ART) versus ART alone in HIV-infected patients with 200
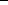
500 CD4+ cells. [Abstract We.B.292] In: XI International Conference on AIDS, Vancouver, BC, 1996.

27. Deresinski S, Israelski D, Frascino R, et al. Randomized multicenter comparison of treatment of HIV infected patients: activated CD8+ cells plus rhIL-2 plus antiretroviral therapy (ART) v rrhIL-2 plus ART v ART alone [Abstract 206]. In: Second National Conference on Human Retroviruses and Related Infections, Washington, DC, January 29-February 2, 1995.

28. Davey RT, Jr., Chaitt DG, Piscitelli SC, et al. Subcutaneous administration of interleukin-2 in human immunodeficiency type-1 infected persons. J Infect Dis 1997;175:781-89.

29. Davey RT, Murphy RL, Graziano FM, et al. Immunologic and virologic effects of subcutaneous interleukin-2 in combination with antiretroviral therapy. JAMA 2000;284: 183-9.

30. Fauci A. Host factors in HIV disease. In: Programs and Abstracts of the XIII International AIDS Conference; July 9-14, 2000; Durban, South Africa.

31. Montaner J, Hogg R, Yip B, Chab K, Craib K, O'Shaughnessy M. To start or not to start? Diminished effectiveness of anti-retroviral therapy among patients initiating therapy with CD4+ cell counts below 200/mm3. [Abstract LbPeB7050] In: Program and Abstracts of the XIII International AIDS Conference; Durban, South Africa, July 9-14, 2000.

32. Tebas P, Henry K, Nease R, Murphy R, Phair J, Powderly WG. Timing of antiretroviral therapy. Use of Markovian modeling and decision analysis to evaluate the long-term implications of antiretroviral therapy. [Abstract 523] In: Programs and Abstracts of the 7th Conference on Retroviruses and Opportunistic Infections, San Francisco, CA, January 30-February 2, 2000.

33. Henry K. The case for more cautious, patient focused antiretroviral therapy. Ann Intern Med 2000;132:306-11.

34. **Recommendations of the panel on clinical practices for treatment of HIV. Guideline for using antiretroviral agents among HIV-infected adults and adolescents. MMWR 2002; 51: RR-7; 1-55.**

35. **Yeni PG, Hammer SM, Carpenter CCJ, et al. Antiretroviral treatment for adult HIV infection in 2002: updated recommendations of the International AIDS Society-USA panel. JAMA 2002; 288: 222-235.**

36. Pantaleo, G. How immune based interventions can change HIV therapy. Nat Med 1999;5:483-6.

37. Ortiz GM, Nixon DF, Trkola A, et al. HIV-1 specific immune responses in subjects who temporarily contain virus replication after discontinuation of highly active antiretroviral therapy. J Clin Invest 1999;104:R13-18.

38. Clotet B. Surrogate marker outcomes with repeated treatment interruptions. Presented at the 27th AIDS Clinical Trials Group Meeting, Washington, DC, July 24-27, 1999.

39. Rosenberg E, Billingsley J, Caliendo A, et al. Vigorous HIV-1-specific CD4 T cell responses associated with control of viremia. Science 1997;278:1447-50.

40. R, Safrit J, Cao Y, et al. Temporal associations of cellular immune responses with the initial control of viremia in primary human immunodeficiency virus type 1 syndrome. J Virol 1994;68:4650-55.

41. Liu Z, Cumberland W, Hultin L, et al. Elevated CD38 antigen expression on CD8+ T cells is a stronger marker for the risk of chronic HIV disease progression to AIDS and death in the Multicenter AIDS Cohort Study than CD4+ T cell count, soluble immune activation markers, or combinations of HLA-DR and CD38 expression. J Acquired Immune Defic Syndr 1997;16:83-92.

42. Fahey J, Taylor J, Manna B, et al. Prognostic significance of plasma markers of immune activation, HIV viral load and CD4 T cell measurements. AIDS 1998;12:1581-90.

43. Salaszar-Gonzalez J, Martinez-Maza O, Nishanian P, et al. Increased immune activation precedes the inflection point of CD4 T cells and the increased serum virus load in human immunodeficiency virus infection. J Infect Dis 1998;178:423-30.

**44. Grossmann Z, Meier-Schellersheim M, Sousa AE, et al. CD4+ T-cell depletion in HIV infection: Are we closer to understanding the cause? Nat Med 2002;8:319-23.**

**45. Hazenberg MD, Hamann D, Schuitemaker H, et al. T cell depletion in HIV-1 infection: how CD4+ T cells go out of stock. Nat Immunol 2000;1:285-89.**

**46. Mohri H, Perelson AS, Tung K, et al. Increased turnover of T lymphocytes in HIV-1 infection and its reduction by antiretroviral therapy. J Exp Med 2001; 194:1277-87.**

**47. Kovacs JA, Lempicki RA, Sidorov IA, et al. Identification of dynamically distinct subpopulations of T lymphocytes that are differently affected by HIV. J Exp Med 2001; 194:1731-41.**

**48.** [**Broussard SR, Staprans SI, White R, Whitehead EM, Feinberg MB, Allan JS.**](http://www.ncbi.nlm.nih.gov:80/entrez/query.fcgi?cmd=Retrieve&db=PubMed&list_uids=11160730&dopt=Abstract) **Simian immunodeficiency virus replicates to high levels in naturally infected African green monkeys without inducing immunologic or neurologic disease. J. Virol. 2001; 75:2262-75.**

**49.** [**Kaur A, Grant RM, Means RE, McClure H, Feinberg M, Johnson RP.**](http://www.ncbi.nlm.nih.gov:80/entrez/query.fcgi?cmd=Retrieve&db=PubMed&list_uids=9811693&dopt=Abstract) **Diverse host responses and outcomes following simian immunodeficiency virus SIVmac239 infection in sooty mangabeys and rhesus macaques. J. Virol. 1998; 72:9597-611.**

**50. Deeks SG, Hoh R, Grant RM, Wrin T, Barbour JD, Narvaez A, Cesar D, Abe K, Hanley MB, Hellmann NS, Petropoulos CJ, McCune JM, Hellerstein MK.. CD4+ T cell kinetics and activation in human immunodeficiency virus-infected patients who remain viremic despite long-term treatment with protease inhibitor-based therapy. J. Inf. Dis. 2002; 185:315-23.**

**51.** [**Sousa AE, Carneiro J, Meier-Schellersheim M, Grossman Z, Victorino RM**](http://www.ncbi.nlm.nih.gov:80/entrez/query.fcgi?cmd=Retrieve&db=PubMed&list_uids=12218162&dopt=Abstract)**. CD4 T cell depletion is linked directly to immune activation in the pathogenesis of HIV-1 and HIV-2 but only indirectly to the viral load. J. Immunol. 2002; 169:3400-6.**

**52. Hazenberg M, Otto S, van Benthem B, Roos M, Coutinho R, Lange J, Hamann D, Prins M, Miedema F. Persistent Immune Activation in HIV-1 Infection is Associated with Naïve T-cell Depletion and Progression to AIDS. (Abstract 336) In: Program and Abstracts of the Tenth Conference on Retroviruses and Opportunistic Infections Feb 10-14, 2003, Boston, MA .**

**53. Wolthers KC, Otto SA, Lens SM, et al. Increased expression of CD80, CD86 and CD70 on T cells from HIV-infected individuals upon activation in vitro: regulation by CD4+ T cells. Eur J Immunol 1996; 26:1700-6.**

**54. Tesselaar K, Arens R, van Schijindel GM, et al. Lethal T cell immunodeficiency induced by chronic costimulation via CD27-CD70 interactions. Nat Immunol 2003; 4:49-54.**

**55. Mostroianni CM, Lichtner M, Mengoni F, et al. Changes in circulating levels of soluble cell adhesion molecules following highly active antiretroviral treatment on HIV-1-infected patients. Clin Immunol 2000; 95:212-17.**

**56. Hazenberg MD, Stuart JW, Otto SA, et al. T-cell division in human immunodeficiency virus (HIV)-1 infection is mainly due to immune activation: a longitudinal analysis in patients before and during highly active antiretroviral therapy (HAART). Blood 2000; 95:249-55.**

**57. Douek DC, Betts MR, Hill BJ, et al. Evidence for increased T cell turnover and decreased thymic output in HIV infection. J Immunol 2001; 167:6663-8.**

**58**. Arno A, Ruiz L, Juan M, et al. Efficacy of low-dose subcutaneous interleukin-2 to treat advanced human immunodeficiency virus type 1 in persons with <250/μl CD4 T cells and undetectable plasma virus load. J Infect Dis 1999;180:56-60.

**59**. Simonelli C, Zanussi S, Comar M, Vaccher E, Giacca M, De Paoli P, Tirelli U. Interleukin-2 in combination with zidovudine and didanosine is able to maintain high levels of CD4 cells and undetectable HIV viremia. AIDS 1998;12:112-3.

**60**. Dean M, Carrington M, Winkler C, et al. Genetic restriction of HIV-1 infection and progression to AIDS by a deletion allele of the CKR5 structural gene. Hemophilia Growth and Development Study, Multicenter AIDS Cohort Study, Multicenter Hemophilia Cohort Study, San Francisco City Cohort, ALIVE Study. Science 1996;273:1856-62.

**61.** McDermott DH, Zimmerman PA, Guignard F, Kleeberger CA, Leitman SF, Murphy PM. The Multicenter AIDS Cohort Study (MACS) Lancet 1998;352:866-870.

**62**. de Roda Husman A, Koot M, Cornelissen M, et al. Association between CCR5 genotype and the clinical course of HIV-1 infection. Ann Intern Med 1997;127:882-90.

**63**. Easterbrook PJ, Rostron T, Ives N, et al. Chemokine receptor polymorphisms and human immunodeficiency virus disease progression. J Infect Dis 1999;180:1096-1105.

**64**. de Roda Husman A, Blaak H, Brouwer M, Schuitemaker H. CC chemokine receptor 5 cell-surface expression in relation to CC chemokine receptor 5 genotype and the clinical course of HIV-1 infection. J Immunol 1999;163:4597-603.

**65. Wood R, Dong H, Katzenstein DA, Merigan TC. Quantification and comparison of HIV-1 proviral load in peripheral blood mononuclear cells and isolated CD4+ T lymphocytes. J Acquired Immune Deficiency Syndromes 1993;6:237-40.**

**66. Birk M, Aleman S, Visco-Comandini U, Sonnerborg A. Proviral HIV-1 dynamics and evolution in patients receiving efficient long-term antiretroviral combination therapy. HIV Med 2000; 1:205-11.**

**67. Lafeuillade A, Poggi C, Chadapaud S, Hittinger G, Khiri H, Halfon P. Impact of immune interventions on proviral HIV-1 DNA decay in patients receiving highly active antiretroviral therapy. HIV Med 2001; 2:189-94.**

**68. Russell RR, Bowmer MI, Nguyen C, Grant MD HIV-1 DNA burden in peripheral**

**blood CD4+ cells influences disease progression, antiretroviral efficacy, and CD4+ T-cell restoration. Viral Immunol 2001;14:379-89.**

**69. Chun TW, Justement JS, Pandya P, Hallahan CW, McLaughlin M, Liu S, Ehler LA, Kovacs C, Fauci AS. Relationship between the size of the human immunodeficiency virus type 1 (HIV-1) reservoir in peripheral blood CD4+ T**

**cells and CD4+:CD8+ T cell ratios in aviremic HIV-1-infected individuals receiving long-term highly active antiretroviral therapy. J Infect Dis 2002; 185:1672-6.**

**70. Gougeon ML, Rouzioux C, Liberman I, Burgard M, Taoufik Y, Viard JP,**

**Bouchenafa K, Capitant C, Delfraissy JF, Levy Y; ANRS 048 Study Group.**

**Immunological and virological effects of long term IL-2 therapy in HIV-1-infected patients. AIDS 2001; 15:1729-31.**

**71. Christopher C, Kindane Y, Conway B, et al. PCR-based assay to quantitate human immunodeficiency virus in peripheral blood mononuclear cells. J Clin Micro 2000; 38: 630-4.**

**72. Zhao Y, Yu M, Miller JW, Chen M, Bremer EG, Kabat W, Yogev R. Quantification of human immunodeficiency virus type 1 proviral DNA by using TaqMan technology. J Clin Microbiol 2002; 40:675-8.**

**73. Desire N, Dehee A, Schneider V, Jacomet C, Goujon C, Girard PM, Rozenbaum W, Nicolas JC.Quantification of human immunodeficiency virus type 1 proviral load by a TaqMan real-time PCR assay. J Clin Microbiol 2001; 39:1303-10.**

**74. Ngo-Giang-Huong N, Deveau C, Da Silva I, Pellegrin I, Venet A, Harzic M, Sinet M, Delfraissy JF, Meyer L, Goujard C, Rouzioux C. French PRIMO Cohort Study Group. Proviral HIV-1 DNA in subjects followed since primary HIV-1 infection who suppress plasma viral load after one year of highly active antiretroviral therapy. AIDS 2001; 15:665-73.**

**75. Brussel A, Mathez D, Broche-Pierre S, Lancar R, Calvez T, Sonigo P, Leibowitch J. Longitudinal monitoring of 2-long terminal repeat circles in peripheral blood mononuclear cells from patients with chronic HIV-1 infection. AIDS 2003; 17:645-52.**

**76. Chun TW, Justement JS, Lempicki RA, Yang J, Dennis G Jr, Hallahan CW, Sanford C, Pandya P, Liu S, McLaughlin M, Ehler LA, Moir S, Fauci AS. Gene expression and viral prodution in latently infected, resting CD4+ T cells in viremic versus aviremic HIV-infected individuals. Proc Natl Acad Sci 2003; 100:1908-13.**

**77. Tierney C, Lathey JL, Christopherson C, Bettendorf DM, D'Aquila RT, Hammer SM, Katzenstein DA, et al. Prognostic Value of Baseline HIV-1 DNA Measurement for HIV Disease Progression in Patients Receiving Nucleoside Therapy. J Infect Disease 2003; 187:1448.**

**78.** Katsikis PD, Garcia-Ojeda ME, Torres-Roca JF, Greenwald DR, Herzenberg LA. HIV type 1 Tat protein enhances activation-but not Fas (CD95)-induced peripheral blood T cell apoptosis in healthy individuals. Int Immunol 1997;9:835-41.

**79.** Westendorp MO, Frank R, Ochsenbauer C, et al. Sensitization of T cells to CD95-mediated apoptosis by HIV-1 Tat and gp120. Nature 1995;375:497-500.

**80.** Bartz SR, Emerman M. Human immunodeficiency virus type 1 Tat induces apoptosis and increases sensitivity to apoptotic signals by up-regulating FLICE/caspase-8. J Virol 1999;73:1956-63.

**81.** Kaneko H, Hishikawa T, Sekigawa I, Hashimoto H, Okumura K, Kaneko Y. Role of tumor necrosis factor-alpha (TNF-alpha) in the induction of HIV-1 gp120-mediated CD4+ T cell anergy. Clin Exp Immunol 1997;109:41-6.

**82.** Wu MX, Schlossman SF. Decreased ability of HIV-1 tat protein-treated accessory cells to organize cellular clusters is associated with partial activation of T cells. Proc Natl Acad Sci U S A 1997;94:13832-7.

**83.** McNeil A SW, Davey RT, Connors M. HIV-1 viremia suppresses antigen-specific proliferation despite maintenance of antigen-specific CD4+ T cells. In: Program and Abstracts of the XIII International AIDS Conference, Durban, South Africa, July 9-14, 2000.

**84.** Valdez H, Smith KY, Landay A, et al. Response to immunization with recall and neoantigens after prolonged administration of an HIV-1 protease inhibitor-containing regimen. ACTG 375 team. AIDS Clinical Trials Group. AIDS 2000;14:11-21.

**85. Dybul M, Chun T, Yoder C, et al. Short-cycle structured intermittent treatment of chronic HIV infection with highly active antiretroviral therapy: Effects on virologic, immunologic, and toxicity parameters. PNAS 2001; 98: 15161-6.**

#### APPENDIX I: IMMUNOLOGIC TESTING

1. Advanced Flow and CCR5 Specimen Collection Time Points:

| Test | Step 1 | | Step 2 | | | Step 4 | |
| --- | --- | --- | --- | --- | --- | --- | --- |
|  | Entry | Week 16 | Week  +2 | Week +4 | Week +8 | Registration to Step 4 | Week +16 |
| Advanced Flow | X | X | X | X | X | X | X |
| CCR5 | X | X |  |  |  |  | X |

1. Advanced Flow Collection

Collect one 3-mL pediatric EDTA (purple-top) tube for the advanced flow cytometry in addition to one 3-mL pediatric EDTA (purple-top) tube for a CD4+ and CD8+ subset determination. No processing of the samples is required. Keep all blood at room temperature. Advanced flow cytometry samples should be sent by overnight carrier to an **IQA**-approved **Immunology Support Laboratory**.

All blood samples must be processed within 30 hours after the draw.

1. CCR5 Quantitation

Collect an additional 3-mL pediatric EDTA (purple-top) tube for the CCR5 Quantitation.

CCR5 Quantitation samples should be sent by overnight carrier to the protocol designated laboratories. Contacts and shipping addresses of the laboratories can be found [in the protocol immunology information sheet](http://aactg.s-3.com/atlimm.htm). Enclose a copy of the Immunology Tracking Form with the sample and fax a copy of the ACTG Specimen Shipment Notice. Prepare and ship specimens in accordance with the International Air Transport Association (IATA) regulations for Infectious Substances. The AACTG Shipping Checklist and Guidelines as well as the AACTG Specimen Shipment Notice can be found at <http://aactg.s-3.com/specship.htm>.

All blood samples must be processed within 30 hours after the draw.

IV. Advanced Flow Panel

Three-color flow cytometry should be run with the following reagents according to the Advanced Flow Method described at:

<http://aactg.s-3.com/pub/download/imm/Imadvflo.doc>

Components:

| Tube | FITC | PE | PerCP |
| --- | --- | --- | --- |
| 1 | IgG1 | IgG1 | IgG1 |
| 2 | IgG1 | IgG1 | CD4 |
| 3 | IgG1 | IgG1 | CD8 |
| 4 | CD4 | CD28 | CD8 |
| 5 | HLA-DR | CD38 | CD8 |
| 6 | CD45RA | CD45RO | CD4 |
| 7 | CD45RA | CD62L | CD4 |
| 8 | CD8 |  |  |
| 9 |  | CD8 |  |
| 10 |  |  | CD8 |

Advanced Flow reagents are available at:

PharMingen

Customer Service: 1-800-848-6227

Contact: Nicole Johnson, 619-597-6630 x4209

Catalog # of advanced flow panel: BP80118Z

NOTE: Further instructions for advanced flow and CCR5 specimen collection and processing can be found in the protocol immunology information sheet located on the protocol specific Web page.

1. Soluble Markers of Activation

**Testing will be done using viably cryopreserved PBMCs and stored plasma samples per section 6.4.11. Samples collected during Step 2 Registration week, week +24, and at time of CD4+ count <350 cells/mm3 on two consecutive visits will be assessed in the following assays:**

**sTNFRII by ELISA on plasma samples**

**ICAM-1 by ELISA on plasma samples**

1. Flow Cytometric Determination of Activation, Cell Turnover and Apoptosis

**Testing will be done using viably cryopreserved PBMCs and stored plasma samples**

**per section 6.4.11. Samples collected during Step 2 Registration week, week +24, and at time of CD4+ count <350 cells/mm3 on two consecutive visits will be assessed in the following assays:**

**CD70-FITC/CD38-PE/CD8-PerCP/CD4-APC**

**IgG1-FITC/IgG1-PE/CD8-PerCP/CD4-APC**

**Ki-67-FITC/CD8-PerCP/CD4-APC**

**BrdU-FITC/CD8-PerCP/CD4-APC(NOTE: This flow marker will only be used when sufficient cells remain after the other antibodies are used.)**

#### APPENDIX II: SPECIMEN PROCESSING OF PLASMA FOR HIV-1 RNA AND FOR FUTURE VIROLOGIC DETERMINATIONS

Collect blood in one 5-mL K3 EDTA tube. **At timepoints listed in Section 6.1, Schedule of Events (Steps 3-7), collect a second 5-mL K4 EDTA tube when plasma is also to be stored for future virologic determinations.**  Centrifuge blood at 800xg for 10 minutes at room temperature. EDTA plasma should be carefully removed from the tube, placed in a sterile conical centrifuge tube, and centrifuged again (at 800xg) for 10 minutes to completely remove platelets and cell debris. Store TWO 1.0-mL aliquots of plasma **for EACH 5-mL EDTA tube collected. Store aliquots of plasma at -**70ºC. Label each aliquot using BLD/**EDT/PL2** as the LDMS specimen code.

**For HIV-1 RNA determinations, ship 2 x 1.0-mL aliquots to a CLIA and VQA-certified virology laboratory. A listing of approved virology laboratories is available at** [**http://aactg.s-3.com/atlvir.htm**](http://aactg.s-3.com/atlvir.htm) and their contact information can be found at <http://aactg.s-3.com/specship.htm>. Ship the sample Monday-Thursday for overnight delivery to arrive by 10:00 am EST the following day. Enclose a copy of the Virology Tacking Form with the sample. In addition, fax a copy of the ACTG Specimen Shipment Notice available at: <http://aactg.s-3.com/specship.htm>. **For plasma aliquots stored for future virologic determinations, ship monthly to the central repository.** Prepare and ship specimens in accordance with the International Air Transport Association (IATA) regulations for Infectious Substances. The ACTG Shipping Checklist and Guidelines also can be found at: <http://aactg.s-3.com/specship.htm>.

#### APPENDIX III: VIROLOGIC AND RESISTANCE TESTING

**I. Resistance Testing**

**To identify the frequency of resistance mutations in plasma RNA following treatment interruption, the first HIV plasma sample with HIV-1 RNA > 500 copies/mL will be amplified, sequenced, and the consensus RNA genotype analyzed to identify drug resistance mutations. To determine whether these are fixed or transient, the sequences obtained at the earliest detection of RNA will be compared to those in the “final” plasma sample obtained at the plateau or maximal value of HIV-1 RNA on Step 2 (off drug). Among individuals with resistance mutations at early escape RNA (during Step 2 at weeks +2 and +4) with reversion to wild type at plateau/zenith RNA, it may be of interest to sequence intervening samples and estimate the rate of reversion through the application of developing minority quasispecies quantification methods.**

A. Specimen Collection Time Points

**Samples will be used from the available specimens collected in section 6.4.11.**

**B. Specimen Type and Handling**

**Available EDTA plasma samples for all subjects during Step 2 at weeks +2, +4, +8, +16, and +24 will be batch shipped at a time designated by the team from the central repository to the Stanford VSL.**

**C. Specimen Testing**

**Testing will be done on the HIV plasma sample with HIV-1 RNA > 500 copies/mL. Testing may also be done on the final plasma sample obtained at the plateau or maximal value of HIV-1 RNA.**

**Additional time points during Step 2 will be analyzed if it is found that there is resistance during week +2 or if there is a difference between the two time points tested.**

**II. Baseline DNA quantification**

**DNA extracted from baseline PBMC may also be sequenced to obtain a consensus PBMC (DNA) RT and PR genotype during Step 1 at weeks 0, 8 and 16 for comparison to the emergent HIV-1 plasma RNA.**

**A. Specimen Collection Time Points**

**Samples will be used from the available specimens collected only by Case Western Reserve University in section 6.4.11.**

**B. Specimen Type and Handling**

**One aliquot of heparanized PBMCs during Step 1 weeks 0 and 16 will be batch shipped at a time designated by the team from the Case Western Reserve University to the Stanford VSL.**

**C. Specimen Testing**

**PBMCs will be tested to measure the amount of proviral DNA per microgram and to measure the amount of resistance in proviral DNA.**

**III. Fitness Testing**

**The University of Minnesota VSL has developed a whole virus fitness assay that is applicable to determine the role and evolution of fitness in circumstances where there is no active selective pressure from protease inhibitors or RT inhibitors. The assay is culture-based with a real-time RT-PCR readout, thus is adaptable only to relatively small numbers of specimens at a time. The assay will be applied to specimens from all positive cell cultures during Step 2.**

**A. Specimen Collection Time Points**

**Samples will be used from the available specimens collected in section 6.4.11.**

**B. Specimen Type and Handling**

**One aliquot from all positive cell cultures during Step 2 week +2 will be batch shipped at a time designated by the team from the Stanford University VSL to the University of Minnesota.**

**C. Specimen Testing**

**Microculture isolates will be analyzed to determine if viral fitness near the initiation of treatment interruption correlates with CD4+ trajectory over the course of Step 2.**

**Determining if fitness shifts from the beginning to the end of Step 2 and whether there is evolution of fitness across interruption steps (e.g., the beginning of Step 2 versus the beginning of Step 4) will be analyzed once a sufficient pool of subjects have completed Step 2 and begun Step 4, respectively, and would also eventually necessitate culturing aliquots of viable frozen PBMCs to obtain viral isolates.**

#### APPENDIX IV: VIRAL CULTURE USING THE QUANTITATIVE PBMC MICRO CULTURE ASSAY

I. PRINCIPLE

The quantitative PBMC micrococulture assay estimates the number of infectious units of HIV per million mononuclear cells (IUPM) in PBMCs. The greater the number of cells needed to produce a positive result, the lower the virus load in the PBMCs. The assay, as described in detail below, is performed in duplicate in a 24-well tissue culture plate using six 5-fold dilutions, beginning with one million patient PBMC. Each sample of patient cells is cocultured with PHA-stimulated normal donor PBMCs for 14 days. The supernatant from each individual well is assayed for viral expression of HIV-l p24 antigen by the standard HIV p24 EIA assay.

II. SPECIMEN REQUIREMENTS

One 8.5-mL ACD (yellow-top) tube will be required for the quantitative PBMC micrococulture assay. The blood must be kept at room temperature until processing and should be processed within 30 hours of collection.

Prior to enrolling subjects in A5102, sites should make processing arrangements with the VSL that they are currently utilizing for other AACTG studies. Sites should send notification of the VSL that they will be using to the team. A listing of VSLs is available at [**http://aactg.s-3.com/atlvir.htm**](http://aactg.s-3.com/atlvir.htm) and their contact information can be found at <http://aactg.s-3.com/specship.htm>. Sites should ship samples Monday - Thursday for overnight delivery to arrive by 10:00 am EST the following day. Enclose a copy of the Virology Tracking Form with the sample. In addition, fax a copy of the ACTG Specimen Shipment Notice available at: <http://aactg.s-3.com/specship.htm>. Prepare and ship specimens in accordance with the International Air Transport Association (IATA) regulations for Infectious Substances. The ACTG Shipping Checklist and Guidelines also can be found at: <http://aactg.s-3.com/specship.htm>.

VSLs should perform the quantitative PBMC micrococulture assay following the consensus assay listed in the Virology Manual at <http://www.niaid.nih.gov/daids/vir_manual>.

#### APPENDIX V: SPECIMEN PROCESSING FOR PBMCs, PLASMA, SERUM AND URINE

Specimen collection time points:

Step 1: Day 0 and weeks 8 and 16

Step 2: Step registration and weeks +2, +4, +8, +16, +24, and Q 8 weeks

Step 3: Step registration and weeks +8, +16, +24

Step 4: Step registration and weeks +8, +16

Step 5: Step registration and weeks +2, +4, +8, +16, +24, and Q 8 weeks

Step 6: Step registration and weeks +8, +16, +24

Step 7: Step registration and week +8, +16, and study termination

I. PBMCs

A. Specimen Type and Handling

Use three 10-mL heparin (green-top) tubes. Specimens should be delivered to the processing laboratory within 4 hours of blood draw. PBMCs must be processed and frozen on the same day as the blood draw. They cannot be shipped overnight to be processed by an off-site laboratory.

For subjects not participating in the immunology substudy, all PBMCs will be viably cryopreserved. For subjects participating in the substudy, at least 3 mL of whole blood must be reserved for intracellular cytokine staining prior to PBMC preparation. In addition, PBMCs prepared from substudy subjects will also be used in real time LPAs and PI apoptosis assays. Sufficient PBMC's must be reserved for the LPA and PI apoptosis assay. All remaining PBMCs are to be cryopreserved.

B. Procedures

1. The heparin tubes should be centrifuged at 400 x g for 10 minutes to separate the cells and plasma. Remove plasma carefully to avoid disturbing the cell layer. Transfer the plasma to a sterile centrifuge tube.

2. After removal of plasma, separate viable PBMCs following the ACTG PBMC Cryopreservation Guidelines available at http:/aactg.s-com/immeth.htm. Freeze six 0.5-mL aliquots of cells in 2-ml cryovials at a concentration of 10 x 106 PBMCs/mL. Store frozen viable cells at -70oC. Label each aliquot as noted below using BLD/HEP/CEL/DMS as the LDMS specimen code. Sites preparing PBMCs must participate in the AACTG Cryopreservation QC Program (<http://aactg.s-3.com/cryo.htm>).

1. Frozen Plasma

Plasma separated from the heparizined tubes after the first centrifugation should be centrifuged again at 800xg for 10 minutes to remove any contaminating cells and platelets. Store ten 1.0-mL aliquots of plasma in 2-mL cryovials and freeze at -70°C. Label each aliquot as noted below using BLD/ HEP/PL2 as the LDMS specimen code.

A sample of the stored frozen plasma also will be used for substudy proinflammatory cytokine determinations (see Appendix **VI**).

1. Serum Freezing

Collect one 10-mL tiger top tube of blood at room temperature. After coagulation, centrifuge the blood at 1200xg for 10 minutes. Store four 1.0-mL aliquots of serum in 2-mL cryovials and freeze at -70°C. Label each aliquot as noted below using BLD/ SST/SER as the LDMS specimen code.

1. Urine Freezing

Collect urine in a sterile cup, aliquot a total of five 1.5-mL of urine using 2-mL cryovials and freeze at -70°C. Label each aliquot as noted below using URN/NON/URN/ as the LDMS specimen code.

1. Specimen Tracking, Labeling, and Shipping

All specimens should be logged into the LDMS by the processing laboratory, and computer labels should be generated that have the LDMS specimen number, Patient Identification Number (PID), Protocol Number, Visit Identification Number (VID), date of draw, **time of collection**, and the LDMS specimen code.

All frozen specimens should be shipped to the central repository for storage. Refer to http://aactg:cure@aactg.s-3.com/specrepos.htm for guidelines and shipping instructions.

#### APPENDIX VI: A5109s ASSAYS

All substudy laboratory assays will be done only at Case Western Reserve University, **Duke University Medical Center, Northwestern University,** orUniversity of North Carolina at Chapel Hill. Substudy assays will be performed at registration to Step 2 and at weeks +2 and +8 of Step 2 (See section 6.2 of the protocol).

1. Modified Advanced Flow

The same 3-mL EDTA tube that was used for the main study advanced flow panel can be used for the additional tubes in the modified advanced flow panel. **However for the registration visit to Step 2, a separate 3-mL EDTA tube will be used to collect a sample for the substudy modified advanced flow.**

For substudy participants only, the following monoclonal antibody combination will be run **real-time** in addition to the ALLRT panel:

HLA-DR-FITC/CD38-PE/CD4-PerCP

1. ProInflammatory Cytokines

An aliquot of heparinized plasma stored for the main study will be utilized for the pro-inflammatory cytokine assays. At the end of the study, aliquots will be requested and shipped by the Central Repository to the laboratory performing the assays.

1. Intracellular Cytokines

A portion of the heparinized whole blood (prior to **the** separation of plasma **for storage**) stored for the main study will be **cultured real-time** for cytokine assays. Therefore, no additional samples are required.

Intracellular cytokine production by CD4+ T-lymphocytes will be determined using the following kit:

BD FastImmune

CD4 Intracellular Cytokine Detection Kit

Anti-HU-IFN-.

Kits are available at: BD Biosciences

Customer Service: 1-800-223-8226

Catalog # 340970

Immunology support laboratories (ISLs) participating in the substudy will activate cells with **unstimulated (no stimulant), SEB, CMV, CMV control, p24, p24 control, HIV-1 MN, and HIV-1 MN control** and then freeze activated cells in FACS Lysing solution at -70°C for later batch staining for intracellular IFN-γ.

Following is the procedure for activation of cells:

1. Label (**8**) 15-mL polypropylene tubes (**unstimulated (no stimulant), SEB, CMV, CMV control, p24, p24 control, HIV-1 MN, and HIV-1 MN control**) and add 0.5 mL of whole blood, an appropriate concentration of stimulant, and 5 μL of CD28/CD49d cocktail. Vortex and incubate 2 hours at 37° C.

2. Add 10 μL of 1 x brefeldin A to each tube, vortex, and incubate 4 hours at 37° C.

3. Add 50 μL of EDTA solution to each tube. Vortex vigorously and incubate 15 minutes at room temperature. Vortex on high setting for 10 seconds.

4. Add 5 mL of 1 x BD FACS lysing solution to each tube, incubate 10 minutes at room temperature and then place tubes in -80°C freezer for batch staining and analysis.

Further information details will be clarified in the Protocol Immunology Information Sheet (PIIS) and directly with the participating ISLs as needed.

D. Apoptosis by Propidium Iodide

A portion of PBMCs separated from heparinized blood from the main study will be utilized for the apoptosis assay. PBMCs will be processed for cell cycle analysis following the spontaneous apoptosis method posted on the AACTG Web site.

Please refer to <http://aactg.s-3.com/pub/download/imm/imapopto.doc>.

E. Lymphocyte Proliferation Assay

A portion of PBMCs separated from heparinized blood from the main study will be utilized for the LPA. LPA will be performed following the consensus method, found on the AACTG Web site (<http://aactg.s-3.com/immmeth.htm)>. The core LPA stimulants (PWM, CMV, CMV control, and CASTA™) will be used.

The sources for antigen supply will be specified in the PIIS and will be available on the A5102 protocol specific Web page.

APPENDIX VII

SAMPLE INFORMED CONSENT TEMPLATE

ADULT ACTG TRIALS

Division of AIDS, NIAID, NIH

TITLE OF CLINICAL TRIAL:

A5102: An Open Label, Pilot Study Utilizing CD4+ T-Cell Counts < 350 Cells/mm3 as the Threshold for Restarting Therapy with Potent Antiretroviral Therapy +/- Interleukin-2 to Determine the Effect of Pulse Therapy on the Characteristics of Treatment Interruptions

PRINCIPAL INVESTIGATOR: PHONE:

INFORMED CONSENT

You are being asked to take part in the research study named above because you are infected with HIV, the virus that causes AIDS. This is a clinical study of an investigational treatment. Before you decide whether or not to take part in this study, we would like to explain the purpose of the study, how it may help you, any risks to you, and what is expected of you.

YOUR PARTICIPATION IS VOLUNTARY

This consent form gives you information about the study, which will be discussed with you. Once you understand the study, and if you agree to take part, you will be asked to sign this consent form. You will be given a copy to keep.

Before you learn about the study, it is important that you know the following:

- Your participation is entirely voluntary;
- You may decide not to take part or to withdraw from the study at any time without losing the benefits of your routine medical care

PURPOSE OF STUDY

The main purpose of this study is to compare the duration of treatment interruption following the use of IL-2 versus no IL-2. Your CD4+ cell count (a CD4+ cell is a type of white blood cell that fights against infections) will be used to determine when your anti-HIV treatment will be stopped and started. It is thought that stopping anti-HIV drugs for periods of time might work like a vaccine and might help to improve the immune system’s reaction to HIV. However, there have been only a few studies looking at the effects of stopping anti-HIV drugs, so it is not known what affects stopping drugs might have. Therefore, this study will also look at the safety and effectiveness of stopping anti-HIV drugs for periods of time.

About half of the people in this study will receive IL-2 and about half will not.

IL-2 is a medication that has been approved by the Food and Drug Administration (FDA) for use in some types of cancer but is not yet approved for use in HIV-1 infection.

You will need to provide your own anti-HIV therapy (a combination of at least three anti-HIV drugs)*.* You must have been on the same anti-HIV drugs for at least 3 months before entering the study, and you must agree to continue therapy during this study. If you change your anti-HIV drugs during the study, you must first discuss it with your study doctor or study nurse.

This study will enroll 80 people and will last approximately 4 years.

PROCEDURES

Screening (within 90 days prior to study entry)

If you decide to enroll in this study and sign this consent form, you will have about 1-2 tablespoons of blood drawn from a vein for laboratory tests including a test to find out the amount of HIV in your blood.

Preentry (within 30 days prior to study entry)

You will be asked about your medical history including HIV-related and non-HIV-related conditions. You will also have a physical exam, and about 1-2 tablespoons of blood will be drawn from a vein for laboratory tests including a test to find out the amount of HIV in your blood (viral load). If you are a woman able to become pregnant, you will also have a pregnancy test. These tests will help to find out if you qualify for the study and if it is safe for you to participate.

You will be told the level of HIV in your blood before you enter the study.

Entry

If you qualify, you will be enrolled in the study. You will be randomized (assigned by chance, as if by the flip of a coin) to one of the following two treatment groups. You will have an equal (1 out of 2) chance of being assigned to either of the two groups.

Treatment Group A: Potent anti-HIV drugs with IL-2

If you are in Group A, you will have three periods of anti-HIV drugs and IL-2 treatment, two periods of anti-HIV drugs only and two periods when your anti-HIV treatment will be stopped.

IL-2 will be given as an injection under the skin twice a day for 5 days. Each 5 day period is called a cycle. You will have three cycles during each IL-2 treatment period. If you complete the study, you will have a maximum of nine IL-2 cycles.

Treatment Group B: Potent anti-HIV drugs without IL-2

If you are in Group B, you will have five periods of anti-HIV treatment and two periods when your anti-HIV treatment will be stopped.

Visit Schedule

You will remain on the same schedule (anti-HIV drugs and IL-2 versus anti-HIV drugs alone) throughout the study. Everyone will stop his or her anti-HIV drugs during the first and third years of the study*.* This period of stopping treatment will be different for each person but it is estimated to be from 32-96 weeks.

This study has **eight** treatment periods or steps. You will need to qualify for each step by having laboratory tests and clinical evaluations done including a physical exam. Most of your visits will occur every 4 weeks for the length of this study. However, there will be times, particularly at the beginning or end of each step, when you will need to have clinic visits more often. At these study visits, 1 to 8 tablespoons of blood will be drawn so that your viral load, CD4+ cell counts and other blood tests can be carefully monitored. For many of your visits, you will not be allowed to eat or drink (except for water and medications) for at least 8 hours before having your blood draws.

If you are in treatment group A, your first injection of IL-2 will be given to you in the clinic, and you will be watched for 30 minutes to make sure you tolerate the injection. If you choose, you will be taught how to give yourself your own injection so that you do not have to return to the clinic for each injection. You will be given a special diary for recording your daily doses of IL-2. Your study doctor or nurse will review these diaries with you after you have completed each IL-2 cycle.

At certain times in the study, safety decisions will be made concerning whether to stop your study treatment based on your viral load staying low and your CD4+ cell levels staying high.If your viral load and CD4+ count do not remain within certain levels, it may not be safe to temporarily stop your anti-HIV drugs. These safety decisions will be discussed with you at the time they are made.

If you have side effects from the study drug or you develop complications of HIV disease, you may be asked to return to the clinic for additional study visits that may require blood to be drawn for laboratory tests.

Early/Premature Discontinuation

If you are in group A and you permanently stop taking IL-2 for any reason, you will be asked to continue in the study and follow the same visit schedule as people in group B. You must return any unused IL-2 to your study doctor or nurse.

If you are in either group and you permanently stop participating in the study, you will be asked to come back for a final visit for safety laboratory tests and clinical evaluations. You will have about 5-6 tablespoons of blood drawn and be asked to give a urine sample at this visit.

**If you are in either group and you no longer wish to follow the study steps, you may be eligible to participate in the observation step. If you are eligible and agree, you will be required to come in to the clinic once every 6 months until the study ends to have blood drawn for your CD4+ count and HIV-1 RNA viral load. You will also have a physical exam and be asked to update your medical information.**

OTHER INFORMATION

Some of your blood that is left over after all required study testing is done may be stored (with usual protectors of identity) and used for ACTG-approved HIV-related research.

**Some of your blood will be stored (with usual protectors of identity) and used for immunologic, metabolic, and virologic testing that is required for this study.**

In addition, if you agree, about 2 1/2 tablespoons of extra blood will be drawn for storage and will be used for future ACTG-approved HIV-related research.

_____Yes _____No

IMMUNOLOGY SUBSTUDY: A5109s

About 16 volunteers who are already enrolled in the main study (A5102) will take part in a substudy (A5109s), which is designed to find out how well white blood cells function during periods when anti-HIV therapy is stopped. White blood cells are cells that help fight infection and are important to the immune system.

**There will be about one teaspoon of blood drawn at your first visit for Step 2. Additional l**aboratory tests will be done on the blood that was already collected as part of A5102. You will be asked to sign a separate consent form.

You may be in the main study without taking part in the substudy.

RISKS and/or DISCOMFORTS

The drugs used in this study may have side effects, some of which are listed below. Please note that these lists do not include all the side effects seen with these drugs. These lists include the more serious or common side effects with a known, or possible relationship. If you have questions concerning the additional study drug side effects please ask the medical staff at your site.

There is a risk of serious and/or life threatening side effects when non-study medications are taken with the study drugs. For your safety, you must tell the study doctor or nurse about all medications you are taking before you start the study and also before starting any new medications while on the study. Also, you must tell the study doctor or nurse before enrolling in any other clinical trials while on this study.

**Immune Modulator**

**Interleukin-2 or** IL-2 **(Proleukin)**

**The most common side effects include:**

- Flu-like symptoms such as fever, chills, **feeling tired**
- **Nausea**
- **Rash**
- **Redness or hard lumps in the areas where IL-2 is injected; the lumps may take weeks to months to resolve.**

Other side effects include:

- **Vomiting, loose or watery stools, stomach pain, loss of appetite**
- **Joint or muscle aches, sweats**
- **Abnormal liver tests, which may mean liver damage**
- **Skin peeling or itching**
- **Sores in the mouth**
- **Flushing**
- **Abnormal function of the thyroid gland**
- **High blood sugar**
- **Anxiety, sleeping problems, change in mood, headache, dizziness**
- **Abnormal kidney function**
- **Swelling, which is caused by leakage of fluid from the blood vessels into the surrounding tissues**
- Low blood pressure and fast heartbeat
- **Decreases in the amount of certain elements in the blood (calcium, magnesium, phosphate, sodium)**
- **A decrease in the number of red blood cells, which may cause weakness, dizziness, and fatigue**
- **A decrease in the number of platelets, which help the blood to clot**
- **Shortness of breath, cough, wheezing and nasal/sinus stuffiness**
- **Inflammation of the gallbladder**

The following side effects have occurred infrequently among people who have received IL-2 for HIV infection:

- Severe allergic reactions, with breathing/swallowing difficulties and hives
- **Serious heart problems, which may include** angina (chest pain)**,** abnormal heart rhythm**,** heart attacks **or decreased** pumping ability
- Development or worsening of certain auto-immune/inflammatory diseases
- **Serious neurological or psychiatric side effects (for example, delerium, attempted suicide, loss of consciousness,** convulsions**, memory loss,** swelling of the brain**, or nerve disorders,** such as disease of the nerve supplying the eye**)**
- **Damage to an area of the brain or to other organs, caused by low blood supply**
- **Kidney failure (which may be fatal)**
- **Worsening of viral hepatitis and liver failure**
- **Blood clot formation in a vein or artery**
- **Pancreatitis**
- **Development or worsening of Kaposi’s sarcoma**
- **Significant bacterial infections**
- Death

**There is a risk of serious and/or life threatening side effects when other medications are taken with the study drugs. It is possible that IL-2 may increase the risk of side effects of certain drugs. Il-2 may lead to a temporary increase in the amount of HIV in your blood.**

**Sometimes, when people who have received IL-2 are given a certain type of dye for X-ray examinations, a reaction may occur that includes symptoms such as fever, chills, nausea, vomiting, itchiness rash, diarrhea, low blood pressure, swelling, and low urine output.**

**IL-2 has not been studied adequately in pregnant women. Animal studies do not suggest**

**that IL-2 causes birth defects, but it is not known whether or not this is true in humans.**

Risks of Anti-HIV Treatment Interruption

The long-term effects of temporarily stopping anti-HIV medications are not yet known. The following are some of the possible risks associated with stopping treatment.

- The HIV viral load may come back to detectable levels (virus rebound) or there may be drug resistance. Drug resistance means that an anti-HIV medication no longer works against HIV.
- Drug resistance is the greatest risk of stopping and restarting medications several times. This may be of extra concern if you are currently taking any drugs called nonnucleoside reverse transcriptase inhibitors, or NNRTIs (for example, delavirdine, efavirenz, and nevirapine). Because NNRTI drugs tend to stay in the body longer than other drugs, this could increase the risk of making the virus resistant to that drug or other drugs. If you are taking nevirapine or efavirenz as part of your anti-HIV drug treatment, you will be asked to stop taking these drugs 24-48 hours prior to stopping all other drugs during the treatment interruption periods.
- There also is a possible additional risk of hypersensitivity associated with stopping and starting an anti-HIV drug regimen that contains abacavir (Ziagen). For this reason, persons on an abacavir-containing anti-HIV drug regimen should discuss all of their treatment options with their physicians before volunteering to enter this study.
- It is possible that your CD4+ cell levels may go down and that your HIV disease may progress more quickly after anti-HIV medications are stopped temporarily. It is also possible that the number of cells in which the virus hides may increase.

Risk of Injections Under Skin

- Pain, redness, or swelling at the injection site
- Damage to skin or the blood vessels, nerves, or fat under skin Infection

Risks of Transmitting Disease

The risk of giving an injection by needle at home is that there is a small chance of a needle stick injury, which can cause an infectious disease (such as HIV and/or hepatitis C virus), to non-infected people in your household. Clinic staff will review with you what to do if there is an accidental needle stick. You must dispose of used needles in a special container.

Risks of Drawing Blood

Taking blood may cause some discomfort, bleeding, or bruising where the needle enters the body and in rare cases, fainting or infection.

PREGNANCY

It is not known if the drug or drug combinations used in this study harm unborn babies. If you are having sex that could lead to pregnancy, you must agree not to become pregnant or make a woman pregnant.

Some of the drugs that you will take as part of this study may make some hormonal birth control drugs less effective. This type of birth control is given by pills, shots, or placed under the skin. This means that you cannot depend on hormonal methods of birth control alone. You must use another method or an additional method.

You and your partner must use reliable birth control that you discuss with the study staff. You must continue to use birth control until 30 days after stopping the study drug.

Because this study includes a period of anti-HIV treatment interruption, and not being treated for your HIV infection during pregnancy or breast-feeding may increase the risk of transmission of HIV to the baby, women who are pregnant or breast-feeding their babies may not join the study. If you can become pregnant, you must have a pregnancy test before you enter this study. The test must be negative.

If you think you may be pregnant at any time during the study, tell your study staff right away. If your pregnancy is confirmed and you are on Step 2 or 5 (the treatment interruption part of the study), you should begin anti-HIV treatment, as discussed between you and your doctor, right away. If you are on a treatment step, you and your doctor will review your anti-HIV treatment and make any necessary changes required by the pregnancy. Some anti-HIV drugs are not recommended during pregnancy. The study staff will talk to you about your choices. However, you will be required to discontinue your participation in this study.

Breast-feeding

Women who are breast-feeding their babies may not join the study. Taking IL-2 does not guarantee that you will decrease the risk of passing HIV through your breast milk to your baby. In addition, it is unknown whether IL-2 passes through the breast milk and produces bad effects in the infant.

BENEFITS

Taking part in this study may lower your viral load, improve your immune system, and/or allow you to have longer periods off anti-HIV therapy. You may also have fewer side effects from anti-HIV therapy, since you would be using anti-HIV medications less frequently. No guarantee can be made for any of the potential benefits listed. You may receive no benefit from this study. However, knowledge gained from this study may in the future help others with HIV or AIDS.

NEW FINDINGS

You will be told of any new information learned during the course of the study that might cause you to change your mind about staying in the study. At the end of the study, you will be told when study results may be available and how to learn about them.

REASONS WHY YOU MAY BE WITHDRAWN FROM THE STUDY WITHOUT YOUR CONSENT:

- the investigator decides that continuing in the study would be harmful to you;
- you need a treatment not allowed on this study;
- you are unable to keep appointments or take study drugs as instructed;
- you have a bad side effect to study treatments;
- you do not meet the eligibility criteria for each step
- you become pregnant;
- the study is cancelled by the FDA, the National Institute of Allergy and Infectious Diseases (NIAID), or the pharmaceutical company supplying study treatment; and/or
- other administrative reasons.

ALTERNATIVES TO PARTICIPATION

Alternatives to being in this study include taking one or more of the FDA-approved drugs for HIV infection available by prescription from your health care provider, or you may volunteer for another study if you qualify. Before you decide to take part in this study, your doctor will give you information about the benefits and risks of the treatments available at your site.

COSTS TO YOU

There is no cost to you for the study treatment (IL-2) or study-related visits, examinations, or laboratory tests in this study. Medical costs of anti-HIV drugs and treatment outside of this study will be charged to you or your health insurance company.

CONFIDENTIALITY

Your research records will be confidential to the extent permitted by law. You will be identified by a code, and personal information from your records will not be released without your written permission. You will not be personally identified in any publication about this study. However, your records may be reviewed, under guidelines of the Federal Privacy Act, by the FDA, the Division of AIDS at NIH, study monitors and the company supplying IL-2 for this study.

RESEARCH-RELATED INJURY

If you are injured as a result of being in this study, the (name of the clinic) will give you immediate necessary treatment for your injuries. The cost for this treatment will be charged to you or your insurance company. You will then be told where you may receive additional treatment for injuries. There is no program for monetary compensation or other forms of compensation for such injuries.

PROBLEMS OR QUESTIONS

If you ever have questions about this study or in case of research-related injuries, you should contact (name of investigator) at (telephone number), or if you have questions about research subjects’ rights you can call (name and title of IRB member) at (telephone number)

SIGNATURE PAGE

This page is only a suggested signature page. Sites may use their own signature page.

If you have read the informed consent (or if you have had it explained to you) and understand the information, and you voluntarily agree to join this study, please sign your name below.

Volunteer's Name Volunteer's Signature Date

(typed or printed)

OR OR

Volunteer's Legal Guardian Legal Guardian's Date

or Representative’s Name Signature

(typed or printed)

Witness's Name Witness's Signature Date

(typed or printed)

NOTE: This consent form with the original signatures MUST be retained on file by the principal investigator. A copy must be given to the volunteer. A copy should be placed in the volunteer's medical record, if applicable.

The Division of AIDS strongly encourages a witness for the person’s signature.

Subjects participating in A5102 may also volunteer to participate in A5109s.

If you choose to take part in the A5109s substudy, please sign below:

Volunteer's Name Volunteer's Signature Date

(typed or printed)

OR OR

Volunteer's Legal Guardian Legal Guardian's Date

or Representative’s Name Signature

(typed or printed)

Witness's Name Witness's Signature Date

(typed or printed)

NOTE: This consent form with the original signatures MUST be retained on file by the principal investigator. A copy must be given to the volunteer. A copy should be placed in the volunteer's medical record, if applicable.

The Division of AIDS strongly encourages a witness for the person’s signature
